# Modeling Parasite Dynamics on Farmed Salmon for Precautionary Conservation Management of Wild Salmon

**DOI:** 10.1371/journal.pone.0060096

**Published:** 2013-04-05

**Authors:** Luke A. Rogers, Stephanie J. Peacock, Peter McKenzie, Sharon DeDominicis, Simon R. M. Jones, Peter Chandler, Michael G. G. Foreman, Crawford W. Revie, Martin Krkošek

**Affiliations:** 1 Department of Zoology, University of Otago, Dunedin, Otago, New Zealand; 2 Department of Biological Sciences, University of Alberta, Edmonton, Alberta, Canada; 3 Mainstream Canada, Campbell River, British Columbia, Canada; 4 Marine Harvest Canada, Campbell River, British Columbia, Canada; 5 Pacific Biological Station, Fisheries and Oceans Canada, Nanaimo, British Columbia, Canada; 6 Institute of Ocean Sciences, Fisheries and Oceans Canada, Sidney, British Columbia, Canada; 7 Atlantic Veterinary College, University of PEI, Charlottetown, Prince Edward Island, Canada; 8 Department of Ecology and Evolutionary Biology, University of Toronto, Toronto, Ontario, Canada; Institute of Marine Research, Norway

## Abstract

Conservation management of wild fish may include fish health management in sympatric populations of domesticated fish in aquaculture. We developed a mathematical model for the population dynamics of parasitic sea lice (*Lepeophtheirus salmonis*) on domesticated populations of Atlantic salmon (*Salmo salar*) in the Broughton Archipelago region of British Columbia. The model was fit to a seven-year dataset of monthly sea louse counts on farms in the area to estimate population growth rates in relation to abiotic factors (temperature and salinity), local host density (measured as cohort surface area), and the use of a parasiticide, emamectin benzoate, on farms. We then used the model to evaluate management scenarios in relation to policy guidelines that seek to keep motile louse abundance below an average three per farmed salmon during the March–June juvenile wild Pacific salmon (*Oncorhynchus* spp.) migration. Abiotic factors mediated the duration of effectiveness of parasiticide treatments, and results suggest treatment of farmed salmon conducted in January or early February minimized average louse abundance per farmed salmon during the juvenile wild salmon migration. Adapting the management of parasites on farmed salmon according to migrations of wild salmon may therefore provide a precautionary approach to conserving wild salmon populations in salmon farming regions.

## Introduction

Global finfish aquaculture production grew by 65% between 2000 and 2010 [1], [2], and likely will continue to grow [3], [4]. Management of parasites is a challenge for finfish farms and as the density of aquaculture production grows, the risk of infectious disease may increase [5]. In some cases, parasites on finfish farms may pose a greater threat to wild fish than to farmed fish [6], [7]. Sea lice (*Lepeophtheirus salmonis*) routinely infect farmed Atlantic salmon (*Salmo salar*) [8], but in British Columbia, Canada, sea lice are seldom a production or health concern for Atlantic salmon on farms [6]. Rather, wild Pacific salmon (*Oncorhynchus gorbuscha*, *O. keta, O. kisutch*) that migrate near to Atlantic salmon farms may face an elevated risk of sea louse infection [9], [10], [11], [12]. Sea louse infection may be associated with lethal or sub-lethal effects [13], [14], [15], and infestations have been linked to Pacific salmon population declines [7], [9], [16], [17], [18]. Some controlled laboratory experiments, however, show no evidence of increased mortality among juvenile pink salmon infected following artificial exposure [19], [20], and there is disagreement about the extent to which productivity in wild stocks is affected [21], [22], [23], [24], [25], [26]. Sea louse management in British Columbia has sought to limit the exposure of wild Pacific salmon to high densities of sea lice by limiting the average number of motile lice per fish on Atlantic salmon farms [27].

Methods to reduce the exposure of wild salmon to sea lice on farmed salmon include suppressing louse abundance on farmed salmon and separating salmon farms from wild salmon migration routes. Costello [13] reports industry best practices for sea louse control that include the fallowing of salmon farms between stocking cycles, the co-stocking of cleaner-fish (*Labridae* spp.) with farmed salmon to consume lice on infected hosts, the timely administration of paraciticides to prevent sea louse epidemics, and the careful selection of farm sites to reduce transmission among farms or between farmed salmon and wild populations. Fallowing between harvest and stocking, stocking only a single year class, and removal of unhealthy fish are practiced on salmon farms in British Columbia to promote farmed salmon health [28]. Although co-ordinated fallowing has drawbacks for commercial production, its practice may reduce infection pressure on wild salmon [29], [30], [31]. No known species of cleaner-fish is available in British Columbia, and importing cleaner-fish may threaten biosecurity [32]. Transmission of sea lice among farms and between farmed and wild salmon may be routine, despite siting precautions [33], [34]. Paraciticide treatment administered to farmed salmon remains the most common method to suppress louse abundance on salmon farms. In British Columbia, the only such paraciticide licenced for use on salmon farms is the chemotherapeutant SLICE®, administered in farmed salmon feed [35].

SLICE® is an aquaculture premix of 0.2% emamectin (4″-deoxy-4″epimethy-laminoavermectin B_1_) benzoate (EMB) administered orally to farmed salmon. Commercial field trials and use of SLICE® have demonstrated effective suppression of chalimus and motile life stages of sea lice on salmon farms in Norway [36], Scotland [37], Canada [35] and the United States [38]. A typical treatment dosage is 50 µg kg^−1^ fish biomass for seven consecutive days [36], [37], [39]. Average concentrations of EMB in the blood plasma of farmed salmon appear to vary widely with farm site and season, but show no association with individual fish mass [39]. Although reports of SLICE® efficacy suggest typical reductions in louse abundance of 89–100% compared to pre-treatment levels [36], [37], [38], [40], evidence of sea louse resistance to SLICE® and decreased efficacy of treatment is established or growing in Scotland [40], Atlantic Canada [41], [42], Ireland and Norway [35]. In British Columbia, SLICE® has remained effective for louse suppression on salmon farms [35], [43], and efforts are being made to tailor the timing of its use to forestall sea louse resistance [44].

The timing of SLICE® treatments on salmon farms is also important to reduce louse abundances along wild salmon migration routes during the annual juvenile wild salmon migration. Juvenile pink (*O. gorbuscha*) and chum (*O. keta*) salmon hatch in rivers and migrate immediately from freshwater to open marine waters during March–June each year [11], [29]. During the annual migration, these juvenile wild salmon migrate through inshore marine waters in the Broughton Archipelago region of British Columbia and pass near to salmon farms [11], [29]. Juvenile pink and chum salmon are at their most vulnerable to negative effects of sea louse infection during the migration due to their small size and recent marine transition [45], [46]. Juvenile coho salmon (*O. kisutch*) are generally larger than migrating juvenile pink and chum salmon because they spend an additional year in freshwater before entering the marine environment, but juvenile coho salmon can face increased sea louse exposure through trophic transmission of sea lice during predation on infected juvenile pink and chum salmon [14]. The timing of treatment to suppress sea louse abundance on salmon farms, therefore, is important to the protection of juvenile Pacific salmon during the annual March–June migration.

Winter treatment may prove effective both to reduce louse abundance on migration routes in advance of the March–June juvenile wild salmon migration [47], and to minimize average annual sea louse abundance on farms [48]. In a study of two salmon farms in the Broughton Archipelago, Krkošek et al. [47] found that maximum reductions in louse abundance on farms lagged SLICE® treatment by 1–3 months, suggesting that treatment to suppress louse abundance prior to the migration ought to take place in January. Sea louse ecology and studies of louse suppression on farms suggest similar timing to utilize SLICE® most effectively. In his review of sea louse ecology, Costello [13] suggested that treatment during winter is important to reduce louse numbers on farms because female sea lice tend to grow larger and produce more eggs during the winter than during other seasons. Peacock et al. [31] found that an increase over time in the proportion of treatments taking place during October–March was associated with a corresponding decrease in average annual sea louse abundance on farmed salmon and wild juvenile salmon in the Broughton Archipelago. These findings suggest that winter treatment on salmon farms may be important for juvenile Pacific salmon. A framework to understand sea louse dynamics in relation to SLICE® treatment and abiotic factors is therefore desirable to inform the timing of treatment on farms for the conservation management of Pacific salmon.

Mathematical models for sea louse population dynamics on salmon farms can provide valuable insight for sea louse suppression and wild salmon conservation. Although louse population dynamics in the Broughton Archipelago may be sensitive to temperature, salinity, and host density both before and after treatment, previous models for louse population dynamics on farms have not included these effects [47]. Temperature and salinity can influence demographic rates for sea lice on Atlantic salmon [49], [50], but effects may be magnified by extremes. For example, effects of temperature on louse abundance are weak or absent in Scotland where winter temperatures rarely fall below 5°C [49], [51], [52], but are strong and negative in Norway where winter temperatures commonly fall below 2°C [49], [53]. Host-density effects, if present, may be local or regional in scale, but modeling louse transmission among farms can be complex. Jansen et al. [53] found a positive association between regional host density and louse abundance on individual farms in Norway, suggesting regional population dynamics, but Revie et al. [51] found no such association in Scotland, suggesting local dynamics only. Our understanding of louse population dynamics may be improved by a model that explicitly accounts for the effects of temperature, salinity, local host density, and treatment on the population growth rates of sea lice on Broughton Archipelago salmon farms.

In this paper we develop a model for sea louse population dynamics across 25 salmon farms in the Broughton Archipelago, British Columbia. We model sea louse population growth rates over time and account for the effects of sea surface temperature and salinity, host density, and SLICE® treatments. The model is applied to seven years of monthly sea louse abundance data spanning 2002–2008. We use the model to assess the optimal timing for SLICE® treatment to reduce motile sea louse abundance in the Broughton Archipelago during the juvenile wild salmon migration, and we use the regulatory limit of three motile sea lice per farmed salmon currently applied in British Columbia [35] as a guideline. The results provide empirical guidelines for the optimal timing of SLICE® treatment for precautionary conservation management of wild salmon within the Broughton Archipelago.

## Methods

### Data

**Figure 1:**
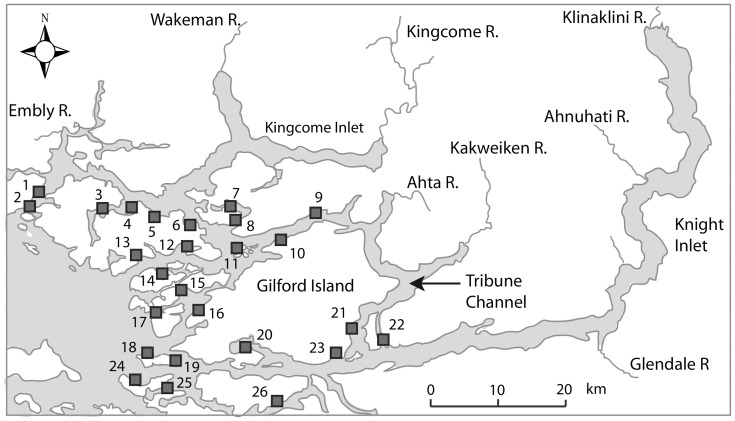
Location of salmon farms in the Broughton Archipelago, BC. Pre-treatment data come from farms 1–4, 6, 8, 9, 11, 13, 15, 17, 18, 20–26. Post-treatment data come from farms 1–4, 6, 9, 11, 13, 17–24. Average temperature and salinity (Table 3) correspond to data from farms 1–9, 11–26.

The data, published previously [25], were collected at 25 salmon farms during 2002–2008 in the Broughton Archipelago (Figure 1). Data were collected monthly at each farm following industry standards similar to those described in Krkošek et al. [47]. Farmed salmon were grouped by cohort, namely all fish at an individual farm during one stock–harvest cycle. At each farm, fish health technicians and aquaculture personnel collected monthly estimates of the average number of motile sea lice (*L. salmonis*) per farmed salmon, the number of farmed salmon per farm, the age of the cohort, the local sea surface temperature and salinity, and the presence or absence of treatment with SLICE®. From these data, we estimated for each month at each farm the growth rate of the mean abundance of motile sea lice per farmed salmon and the total cohort surface area of farmed salmon.

We chose cohort surface area as a proxy for host density because cohort surface area directly influences sea louse settlement opportunity. The findings of Tucker et al. [54] suggest that for high infection pressure, host surface area is a more accurate predictor of sea louse settlement and survival on Atlantic salmon than is host biomass. Calculations for monthly growth rates and cohort surface area were made as follows.

The monthly growth rates for the average abundance of motile sea lice per farmed salmon (*r_t_*) on a given farm in month *t* was calculated as 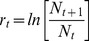 where *N_t_* is the average motile louse count per farmed salmon in month *t*. The growth rate (*r_t_*) was omitted whenever *N_t_* or *N_t_*_+1_ was missing.

Cohort surface area for each month at each farm was calculated using cohort age, estimated individual fish mass, estimated individual surface area by mass, and monthly number of fish per cohort. Individual mass (g) was estimated using age-class growth rates for farmed Atlantic salmon [55] at 8°C average sea temperature. Average individual surface area (cm^2^), was estimated from mass, using a surface area formula for hatchery-reared Atlantic salmon, AREA = 14.93(MASS)^0.59^ [56]. The cohort surface area in each month was the product of the estimated surface area per farmed salmon and the number of farmed salmon per cohort.

Cohort surface area, temperature, and salinity were each standardized to a mean of zero and a standard deviation of one over the dataset. This standardization allowed direct comparison among the effect sizes of the covariates. Under the standardization, the effect size of a covariate corresponded to the change in the response variable induced by a one-standard-deviation change in that covariate. Without standardization, the effect size of a covariate would have corresponded to the change in the response variable induced by a one-unit change in that covariate, making comparison among covariates measured in different units difficult.

Following standardization, months with missing values for the growth rate (*r_t_*), cohort surface area (*A_t_*), temperature (*T_t_*), or salinity (*S_t_*) were removed. The remaining data were then divided into two sets that were analyzed independently: 250 cohort-months of pre-SLICE®-treatment data representing 51 stock-harvest cycles (19 farms; 2003–2008) and 86 cohort-months of post-SLICE®-treatment data representing 32 stock-harvest cycles (16 farms; 2002–2008). The pre-treatment data covered each cohort on each farm from initial stocking up to but not including the month of first treatment. The post-treatment data covered the 3 months following but not including the month of first treatment for each cohort. The spatial distribution of SLICE® treatments over time specifying the data used in this study may be found as an animation in the supplemental information [Animation S1].

### Analysis

We modeled the sea louse population growth rate (*r_t_*) using a linear hierarchical model with random effects for the cohort and farm. The random effects controlled for 1) non-independence among repeated measurements of mean sea louse abundance per fish on a single cohort, and 2) individual farm characteristics, such as flow patterns, that may consistently affect growth rates at a particular site. We then evaluated the influence of cohort surface area, temperature, salinity, and number of months since treatment on sea louse population growth. For the analysis of pre-treatment data, the covariate for the number of months since treatment was excluded. The full linear mixed model was
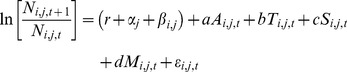
(1)where *N_i,j,t_* is the average abundance of motile sea lice per farmed salmon in cohort *i* on farm *j* during month *t*; *r* is the intrinsic rate of growth in sea louse abundance per fish; *α_j_* and *β_i,j_* are random effects for cohort nested within farm that are normally distributed with means of zero and variances that are estimated from the data; *A*, *T*, *S*, and *M* are the cohort surface area, sea-surface temperature, salinity, and number of months since treatment respectively; *a*, *b*, *c*, and *d* are parameters that were estimated from the data to determine the magnitude and direction of the effects of *A*, *T*, *S*, and *M* on the growth of sea louse abundance per fish, respectively; and ε*_i,j_* is the residual normally-distributed variation.

We fit the full model and models corresponding to all subsets of the covariates to the pre-treatment (8 models) and post-treatment (16 models) data using maximum likelihood [57] in the statistical programming environment *R* [58]. For each model, we calculated the Akaike Information Criterion (AIC), and ranked the models by their Akaike weights [59]. We used multi-model inference [60] over all models to generate model averaged parameter estimates and standard errors for the intrinsic rate of growth (*r*) and effect sizes (*a*, *b*, *c*, *d*) of the covariates [59]. We estimated parameters and standard errors for the pre-treatment and post-treatment data separately.

After estimating parameters and standard errors, we simulated sea louse population dynamics in relation to farm treatment. Simulations were chosen to inform sea louse management for adherence to a maximum average three motile sea lice per farmed salmon guideline [35], and for the minimization of sea louse abundance on salmon farms during the annual juvenile wild salmon migration. For these simulations, we converted equation (1) into a deterministic model for sea louse population dynamics
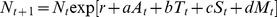
(2)that incorporates the model averaged estimates for the parameters in equation (1). We then extended equation (2) to a stochastic model for sea louse population dynamics
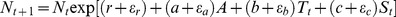
(3)by including normal random variables *ε_r_, ε_a_, ε_b_*, and *ε_c_* with means of zero and standard deviations equal to the estimated standard errors for the intrinsic rate of growth and effect sizes of cohort surface area, temperature, and salinity, respectively.

Using equation (3) and the pre-treatment parameter estimates and standard errors, we simulated scenarios of sea louse population growth in the absence of treatment. Simulations were conducted 10 000 times for different initial sea louse abundances and dates. We used initial conditions of 0.5, 1, and 2 motile sea lice per farmed salmon at dates between December 1^st^ and June 30^th^. We simulated monthly growth for average motile sea louse abundance until June 30^th^, the end of the juvenile salmon migration season. Cohort surface area was set to its standardized mean (*A* = 0) for each simulation. Values for temperature and salinity were chosen for each month in each simulation by random selection from the standardized observed values for the corresponding month in the combined pre-treatment and post-treatment data. By observing the proportion of simulations for each set of initial conditions that led to an average abundance of three or more motile sea lice per farmed salmon on June 30^th^, the simulations convey an estimate of the probability that a mean sea louse abundance of 0.5, one, or two motile sea lice per farmed salmon on a particular date will lead to growth that exceeds the three motile sea lice guideline during the juvenile wild salmon migration (March–June). These estimated probabilities may be useful to managers for understanding the probability of exceeding the three motile sea lice per farmed salmon guideline during the juvenile wild salmon migration.

Using equation (2) and the post-treatment parameter estimates, we simulated scenarios of sea louse population growth for four months following treatment. We used the initial condition of three motile sea lice per farmed salmon, corresponding to the regulatory guideline that triggers treatment or harvest [35]. Simulations were conducted with constant temperatures and salinities, and we evaluated the sensitivity of results to a range of 6–12°C and 15–30 PSU. Cohort surface area was set to its standardized mean (*A* = 0).

The effect of treatment ended when the post-treatment growth rate became equal to the pre-treatment growth rate, where pre- and post-treatment growth rates are calculated as
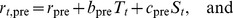
(4)

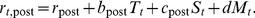
(5)


When the effect of treatment ended, the months since treatment effect (*dM_t_*) was excluded and the post-treatment parameters (*r*_post_, *b*_post_, *c*_post_) were replaced by the pre-treatment parameters (*r*_pre_, *b*_pre_, *c*_pre_) to ensure that the growth rate in each simulation did not exceed the pre-treatment growth rate.

In a second set of post-treatment simulations using an altered method and equation (2), we simulated the effects of treatment near the March–June juvenile salmon migration. We set the initial condition of three motile sea lice per farmed salmon in a particular month, December, January, February or March, and simulated forwards, varying the temperature and salinity according to the average monthly conditions across farms. As with previous simulations, when the post-treatment rate reached the pre-treatment growth rate, signaling an end of the treatment effect, we replaced the post-treatment parameters (*r*_post_, *b*_post_, *c*_post_) by the pre-treatment parameters (*r*_pre_, *b*_pre_, *c*_pre_) and excluded the months since treatment effect (*dM_t_*). Insight based on these simulations may be useful to aquaculture managers for timing the treatment of farmed salmon to reduce sea louse populations prior to and during the March–June juvenile wild salmon migration.

## Results

**Figure 2:**
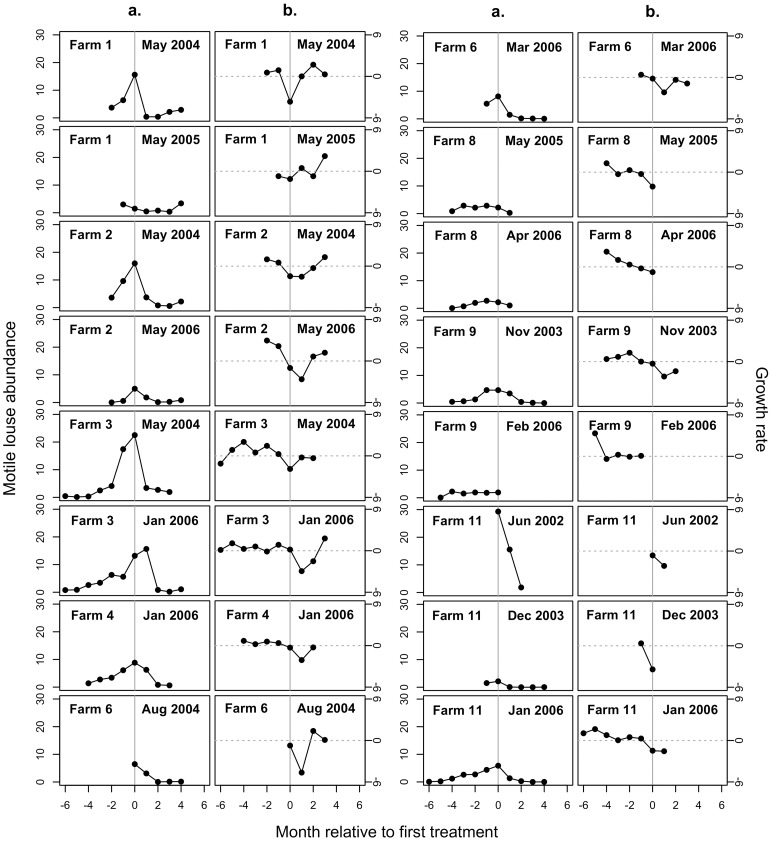
Examples of motile sea louse population dynamics on farms before and after treatment. Sea louse dynamics for 16 farmed salmon cohorts are shown as **a.** average motile louse abundance per farmed salmon, and **b.** growth rate of average motile sea louse abundance per farmed salmon, *r* = ln[*N_t_*_+1_/*N_t_*], where *N_t_* is average sea louse abundance per farmed salmon in month *t*. SLICE® treatment (grey line) was initiated in the month and year specified. Values for the growth rate (*r_t_*) are omitted whenever *r_t_* is undefined (i.e. when *N_t_* = 0 or *N_t +_*_1_ = 0) or missing. Cohorts shown are those with abundance and growth rate data in months immediately preceding or following the first treatment by SLICE® in the pre-treatment or post-treatment data.

**Figure 3:**
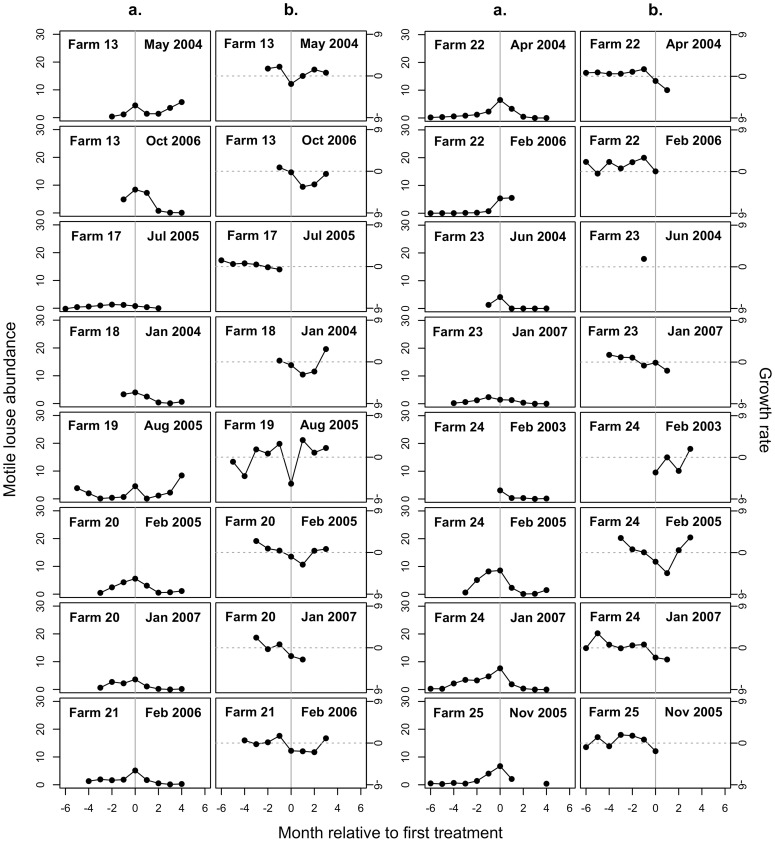
Examples of motile sea louse population dynamics on farms before and after treatment (continued). Sea louse dynamics for 16 farmed salmon cohorts are shown as **a.** average motile louse abundance per farmed salmon, and **b.** growth rate of average motile sea louse abundance per farmed salmon, *r* = ln[*N_t_*_+1_/*N_t_*], where *N_t_* is average sea louse abundance per farmed salmon in month *t*. SLICE® treatment (grey line) was initiated in the month and year specified. Values for the growth rate (*r_t_*) are omitted whenever *r_t_* is undefined (i.e. when *N_t_* = 0 or *N_t +_*_1_ = 0) or missing. Cohorts shown are those with abundance and growth rate data in months immediately preceding or following the first treatment by SLICE® in the pre-treatment or post-treatment data.

On salmon farms in the Broughton Archipelago between 2002 and 2008, the average abundance per farmed salmon of motile sea lice (*Lepeophtheirus salmonis*) typically increased over time until farmed salmon were treated with SLICE® (Figures 2 and 3). Following treatment, the motile sea louse growth rate (*r_t_*) tended to be suppressed below pre-treatment rates for two or three months after which the growth rate tended to return to pre-treatment levels. Following treatment, motile sea louse abundance tended to decline sharply and remained depressed for at least four months.

Comparison of models using AIC revealed model-selection uncertainty, particularly for pre-treatment data (Table 1). Model averaged parameter estimates (and standard errors) for the pre-treatment intrinsic rate of growth (*r*) and effect sizes (*a*, *b*, *c*) of cohort surface area, temperature, and salinity indicated clear positive sea louse population growth prior to treatment. Model-averaged parameter estimates (and standard errors) for the post-treatment intrinsic rate of growth (*r*) and effect sizes (*a*, *b*, *c*, *d*) of cohort surface area, temperature, salinity, and the number of months since treatment indicated clear negative sea louse population growth following treatment, but whose effect depended strongly on the time since treatment (Table 2). The effects of all other covariates on sea louse population growth were small and had large uncertainty, although the effects of temperature and salinity on sea louse population growth were better resolved in post-treatment than pre-treatment data (Table 2).

**Table 1:** Pre-treatment and post-treatment model selection statistics.

Data	Model	NLL	AIC	ΔAIC	Weight
Pre-treatment	r+S	321.4	652.8	0.00	0.200
	r	322.5	653.1	0.24	0.178
	r+T	321.8	653.6	0.80	0.135
	r+A	321.9	653.7	0.87	0.129
	r+A+S	320.9	653.9	1.03	0.120
	r+A+T	321.1	654.2	1.37	0.101
	r+S+T	321.3	654.6	1.75	0.084
	r+A+S+T	320.7	655.5	2.66	0.053
Post-treatment	r+S+T+M	132.7	279.3	0.00	0.309
	r+M	135.3	280.7	1.38	0.155
	r+A+S+T+M	132.5	281.1	1.77	0.127
	r+T+M	134.6	281.1	1.80	0.126
	r+S+M	134.8	281.5	2.23	0.102
	r+A+M	135.1	282.1	2.85	0.075
	r+A+T+M	134.3	282.5	3.23	0.062

Models were ranked by Akaike weight, and the collection of best-supported models corresponding to a 95% cumulative weight is shown. Models were generated for the intrinsic sea louse population growth rate (*r*) and all subsets of the covariates cohort surface area (*A*), temperature (*T*), salinity (*S*), and number of months since treatment (*M*), with the exception that the covariate for months since treatment (*M*) is excluded from the pre-treatment models. Columns show the negative log likelihood values (NLL), Akaike Information Criterion (AIC), the AIC differences (ΔAIC), and the Akaike weights.

**Table 2:** Pre-treatment and post-treatment model averaged parameter estimates.

Data	Rate (*r*)	Area (*a*)	Temp. (*b*)	Salt (*c*)	Month (*d*)
Pre-treatment	0.35 (0.07)	0.09 (0.08)	−0.06 (0.06)	0.08 (0.06)	–
Post-treatment	−2.91 (0.31)	−0.09 (0.14)	0.22 (0.13)	0.22 (0.14)	1.25 (0.16)

Model-averaged estimates are given for the intrinsic rate of growth of average sea louse abundance per farmed salmon (*r*) and the effect sizes for cohort surface area (*a*), temperature (*b*), salinity (*c*), and number of months since treatment (*d*). Standard errors are given in parentheses. Pre-treatment data span from stocking to the month preceding the first SLICE® treatment; post-treatment data span the three months following the month in which treatment takes place.

**Figure 4:**
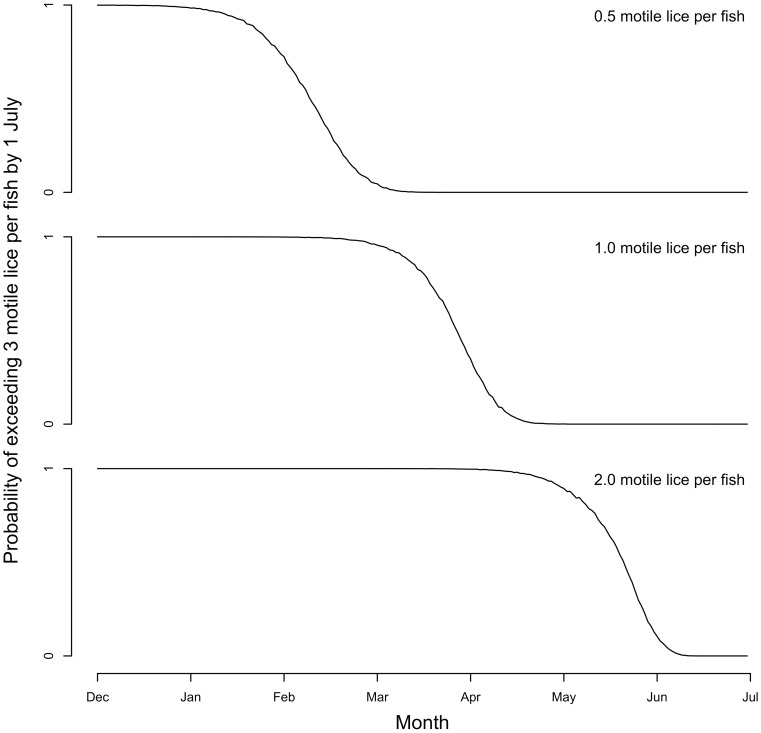
Simulated probability of exceeding three motile sea lice per farmed salmon during the juvenile wild salmon migration in the absence of SLICE® treatment. Probabilities are shown for exceeding a threshold of three motile sea lice per farmed salmon before the end of the juvenile wild salmon migration (June 30^th^) given average sea louse abundances of 0.5, 1, or 2 motile sea lice per fish in a particular month and in the absence of treatment. Probabilities for each month correspond to the proportion out of 10 000 simulations that met or exceeded three motile sea lice per fish by June 30^th^. Simulations are based on the stochastic model in equation (3) with random variation around the growth rate (*r*) and around the parameters for the effects of temperature (*b*) and salinity (*c*) Cohort surface area, *A*, is set to its standardized mean (*A* = 0) and the months since treatment (*M*) is excluded from the simulation. Values for temperature and salinity are chosen for each month in each simulation by random selection from standardized observed values for that month from the data.

Stochastic simulations using equation (3) and the pre-treatment parameter estimates and standard errors (Table 2) at standardized mean cohort surface area (*A* = 0) and with temperature and salinity sampled randomly by month indicated that a lack of treatment in the months preceding the March–July juvenile salmon migration could lead to abundances of sea lice on farmed salmon that exceed the guideline of three motile sea lice per farmed salmon during the juvenile salmon migration window (Figure 4). Our simulations suggest that abundances of 0.5 motile sea lice per farmed salmon prior to February 10^th^, one motile sea louse prior to March 27^th^, or two motile sea lice prior to May 21^st^ may grow to reach or exceed the three motile sea lice guideline during the juvenile wild salmon migration (March–June) with probability >0.50, assuming no treatment is initiated.

**Figure 5:**
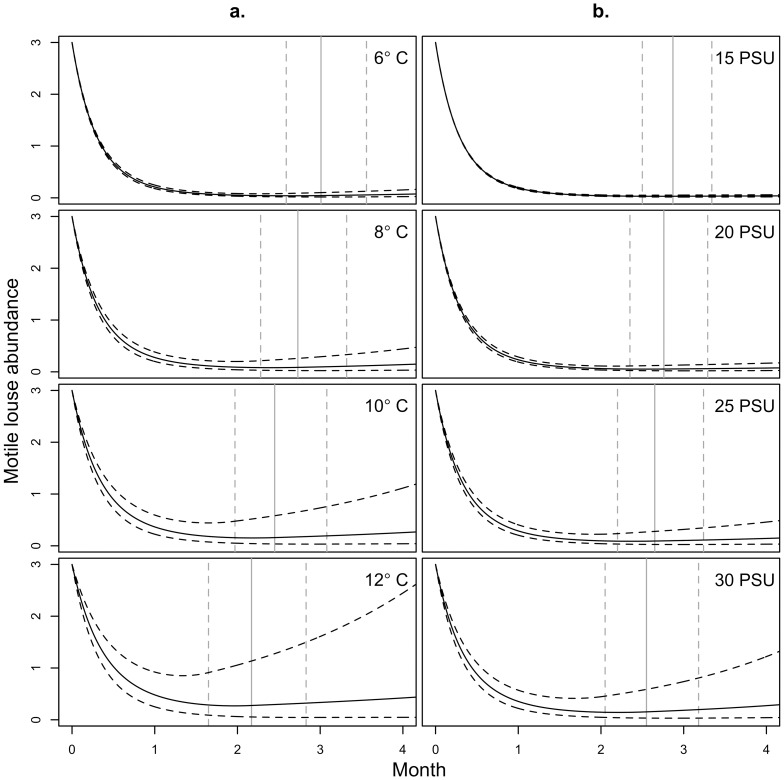
Simulated decline and recovery of sea louse populations at constant temperature and salinity following SLICE® treatment. Simulations are shown for a. salinity held constant at 26.9 PSU and b. temperature held constant at 8.8°C, the average salinity and temperature recorded for the Broughton Archipelago during the study period (Table 3). To generate the simulations, post-treatment estimates for *r*, *b*, *c*, and *d* (Table 2) were used until the effective rate of growth *r*_post_+*b*_post_ *T_t_*+*c*_post_ *S_t_*+*dM_t_* became equal to the pre-treatment (maximum) rate of growth *r*_pre_+*b*_pre_ *T_t_*+*c*_pre_ *S_t_* (Table 2). From this point on the pre-treatment parameters were used, and the effect for the number of months since treatment was omitted. Cohort surface area was set to its standardized mean (*A* = 0) for each simulation. Curved dashed lines correspond to simulations with parameters set one standard error on either side of the mean parameter estimates. Vertical grey lines correspond to the time of transition from post-treatment to pre-treatment parameters (i.e. *t* such that *r*_post_+*b*_post_ *T_t_*+*c*_post_ *S_t_*+*dM_t_* = *r*_pre_+*b*_pre_ *T_t_*+*c*_pre_ *S_t_*) for the mean (solid) and one-standard-error-removed (dashed) simulations.

Simulations for post-treatment sea louse population dynamics indicated that treatment effect, measured as growth rate suppression, lasted 2–3 months depending on ambient temperature and salinity (Figure 5). At average salinity (26.9 PSU) and temperatures within 6–12°C, our simulations suggest that the growth rate for sea louse abundance remained below pre-treatment rates for 2.2–3.0 months. At average temperature (8.8°C) and salinity within 15–30 PSU, the simulated growth rate for sea louse abundance remained below pre-treatment levels for 2.6–2.9 months (Figure 5). SLICE® treatment was predicted to depress mean sea louse abundance per farmed salmon below three motile sea lice for at least four months at average temperature (8.8°C) or average salinity (26.9 PSU) (Figure 5).

**Figure 6:**
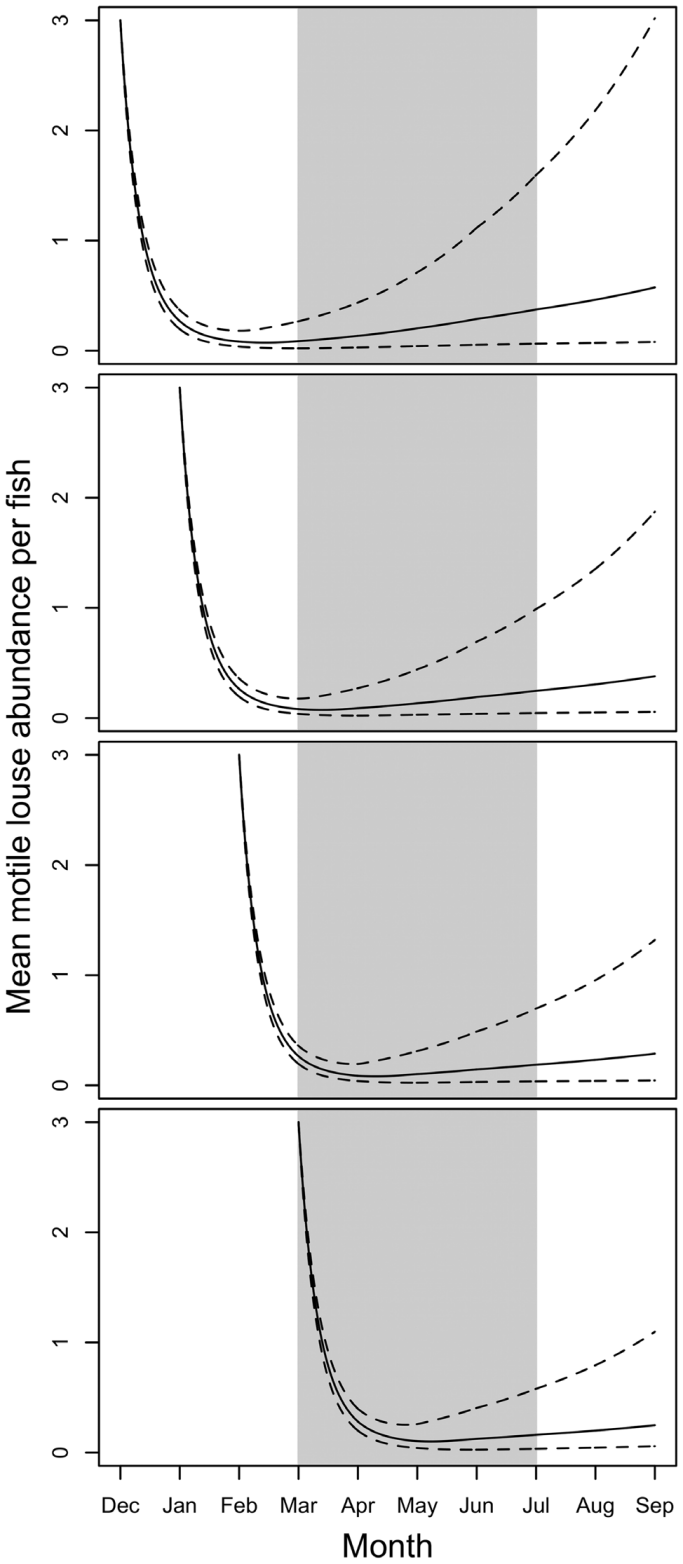
Simulated decline and recovery of sea louse populations at varying temperature and salinity following SLICE® treatment. Simulations correspond to SLICE® treatment initiated in December, January, February, and March in advance of the March–June juvenile wild salmon migration (grey shaded region). Simulations used a combination of post-treatment and pre-treatment parameter estimates as described for Figure 5. Temperature and salinity were varied by month to represent the average monthly conditions across salmon farms during December–September in the Broughton Archipelago. Dashed lines correspond to simulations with parameters set to one standard error on either side of the mean parameter estimates.

Seasonal variability in environmental conditions had implications for sea louse management (Table 2 and Table 3). We found that treatment of farmed salmon in January or early February minimized average sea louse abundance per farmed salmon during the entire March–July juvenile wild salmon migration (Figure 6). The effect of treatment before or during December was predicted to wear off and allow pre-treatment growth rates to resume before or during March, early in the juvenile wild salmon migration. Treatment after mid-February was predicted to delay the suppression of sea louse abundance and leave abundances greater than three times the suppression minimum on March 1^st^, the beginning of the juvenile wild salmon migration.

**Table 3:** Temperature and salinity on Broughton Archipelago salmon farms during 2000–2009.

	T (°C)			S (PSU)		
Month	Mean	SE	Range	Mean	SE	Range
Jan	7.1	(0.6)	6.0–8.0	29.1	(2.8)	21.0–33.0
Feb	7.2	(0.5)	6.5–9.0	28.8	(3.3)	17.0–34.5
Mar	7.4	(0.5)	6.0–9.0	29.5	(3.0)	18.0–34.8
Apr	8.3	(0.6)	7.0–9.8	29.4	(2.9)	18.0–33.5
May	9.3	(0.9)	7.0–11.9	28.0	(4.0)	17.0–34.0
Jun	10.3	(0.9)	8.0–12.5	24.5	(6.0)	10.0–33.0
Jul	10.9	(1.1)	8.7–13.2	22.9	(6.8)	7.3–33.0
Aug	10.9	(1.1)	9.0–14.8	23.3	(6.6)	8.2–33.0
Sep	10.1	(0.9)	8.8–12.3	25.4	(5.8)	7.7–33.0
Oct	9.1	(0.7)	6.7–11.0	25.6	(4.8)	12.3–32.7
Nov	8.1	(0.5)	7.0–10.6	26.8	(4.7)	13.0–33.0
Dec	7.3	(0.6)	5.1–9.8	28.7	(2.7)	21.0–33.0

Monthly averages, standard errors, and range (min–max) are given for recorded temperature and salinity on farms 1–9, 11–26 (Figure 1).

In all simulations (Figures 3, 4, 5), uncertainty in model predictions propagated as time increased, and long-term forecasting of sea louse population dynamics beyond several months based on our model and parameter estimates was less informative.

## Discussion

Precautionary management of parasites on Atlantic salmon farms in British Columbia that suppresses parasite abundance to coincide with the timing of juvenile wild salmon migrations may reduce the risk of infection for wild Pacific salmon. We used a model to understand sea louse population dynamics on salmon farms in the Broughton Archipelago, British Columbia. We found a positive rate of growth for sea louse abundance on farmed salmon in the absence of parasiticide treatment, and a temporarily negative rate of growth following farmed salmon treatment by SLICE®. We found that treatment of farmed salmon by SLICE® tended to depress sea louse abundances for periods commensurate with the duration of the juvenile wild salmon migration. Consequently, the judicious timing of SLICE® treatment on salmon farms may be a viable management option to reduce the transmission of sea lice to migrating juvenile wild salmon. Indeed, we found treatment during January or early February most likely to minimize sea louse abundance on Broughton Archipelago salmon farms during the March–June juvenile wild salmon migration. These findings are consistent with previous studies that identified exponential patterns of sea louse population growth in the absence of treatment [47], and effective sea louse suppression following SLICE® treatment in the Broughton Archipelago [35]. Our findings extend the results of Krkošek et al. [47] to suggest that winter treatment of sea lice, considered among the salmon aquaculture industry best practices [13], [48] for annual sea louse suppression on salmon farms [31], may be an effective strategy to reduce sea louse exposure for migrating juvenile wild salmon generally across salmon farms in the Broughton Archipelago.

Sea louse population dynamics in the Broughton Archipelago appear to be mediated by abiotic factors, specifically temperature and salinity. We detected weak effects for temperature and salinity on pre-treatment sea louse population growth over the ranges encountered in our study (Table 3). Sea lice are generally observed to exhibit higher levels of settlement on hosts, increased survival of larval stages, and higher rates of development at higher ambient temperatures and salinities [49], [50], [61], [62]. The data that informed our parameter estimates were monthly averages that did not capture seasonal or spatial extremes in temperature and salinity. These extremes may play an important role in limiting sea louse population growth. Application of the model to data at a finer temporal resolution that includes extreme abiotic events and sea louse population responses, which are likely to occur on time scales of days or weeks, could therefore be an informative future extension of this study.

Temperature was found to have a positive effect on sea louse population growth rates following SLICE® treatment. As a result, the temperature effect led to shorter periods of sea louse suppression in warmer waters. At least two mechanisms may be responsible for this effect. First, larval settlement and sea louse development are expected to increase with increased temperature [49], [50], leading potentially to shorter generation times and higher rates of population growth relative to populations in cooler waters [13]. Second, the depletion of EMB residue from the body tissue of farmed rainbow trout (*Oncorhynchus mykiss*) appears more rapid at higher temperatures [63]. If EMB depletion from host tissue follows a corresponding pattern in farmed Atlantic salmon, increased temperature could shorten the period during which sea lice populations are suppressed by SLICE®.

We also found that salinity had a positive effect on sea louse population growth rates following SLICE® treatment. As a result, we predicted longer periods of sea louse suppression in less saline waters. This effect may be explained by changes to sea louse demographic rates at low salinity. Hatching, survival, and development rates for sea lice have all been observed to decline at low salinity [61], [62]. Surface salinity in the Broughton Archipelago is strongly influenced by an annual runoff of freshwater, leading to reduced surface salinity starting in late spring (Table 3), although this may be less pronounced in the outer regions of the Broughton Archipelago.

Our simulations of sea louse dynamics for average monthly conditions in the Broughton Archipelago point to an optimal timing for SLICE® treatment to suppress sea louse abundance on farms during the juvenile wild salmon migration. Based on our simulations, the optimal time to administer SLICE® treatment is January or early February. Under this regime, sea louse populations on farms are most likely to be suppressed below three motile sea lice per farmed salmon, consistent with regulatory guidelines, for the duration of the March–June juvenile wild salmon migration. A January or early February treatment strategy could likely reduce infection pressure on wild salmon during the juvenile wild salmon migration.

Harm to wild stocks from exposure to sea lice during the juvenile wild salmon migration may change depending on the timing of exposure. Juvenile pink and chum salmon may be more vulnerable to the effects of sea lice early in their migration [64]. Early exposure can also allow time for reproduction of sea lice on juvenile hosts, further amplifying sea louse populations on wild juvenile salmon [33]. The early exposure of pink and chum salmon, two prey sources for juvenile coho salmon, may in turn increase the exposure of juvenile coho salmon to sea lice [14]. Consequently, efforts to reduce impacts to wild salmon should focus on minimizing sea louse exposure for juvenile Pacific salmon during March–April, the first half of the juvenile salmon migration.

Cohort surface area per farm, a proxy for host density, had no consistent effect on sea louse population growth rates. The lack of effect suggests that host surface area did not limit sea louse population growth, likely because sea louse numbers were kept low by SLICE® treatments. The highest average sea louse counts on farms in the Broughton Archipelago typically occurred immediately prior to treatment, and rarely exceeded 10 sea lice per farmed salmon. By contrast, sea louse counts involving *Lepeophtheirus salmonis* on untreated salmon farms in Scotland have experienced mean sea louse abundances in excess of 30 sea lice per farmed salmon [37]. The observed pattern of mean sea louse population growth truncated by treatment, together with evidence of the potential for much higher sea louse abundances under similar but untreated conditions suggests that sea lice on farmed salmon in British Columbia may be limited primarily by treatment. For this reason, sea louse populations may not approach a carrying capacity, and therefore not be limited by host surface area.

The absence of a strong host-density effect may appear to contradict the recent findings of Jansen et al. [53], who reported that monthly mean counts of sea lice were positively related to the average mass of farmed salmon and local biomass density on salmon farms in Norway. However, we do not expect the relationship that Jansen et al. [53] found for sea louse abundance to hold in our model for growth rates. For example, a positive growth rate that is constant over time will lead to an abundance that increases over time. Therefore, if sea louse population growth were positive on average during a farmed salmon production cycle, when farmed salmon increase in body size, then a positive correlation between fish biomass and sea louse abundance would result even if no effects of fish biomass on the sea louse population growth rate were present. Thus, it is not clear from the present study nor from Jansen et al. [53] that host density has a positive effect on sea louse transmission rates, as would be expected by theory [65].

Dispersal of sea louse larvae due to wind and ocean currents may give rise to sea louse population dynamics that act on a regional scale [34], [53], [66], [67], [68], and regional host density may be more relevant to sea louse population dynamics than host density at a single farm [53]. Mechanistic models of sea louse dispersal among farms can account explicitly for regional host-density effects. Wind and ocean currents in the Broughton Archipelago may lead to complex spatial and temporal patterns of larval sea louse dispersal among salmon farms [68] and make the estimation of regional host-density effects difficult. A hydrodynamic model describing the motion of currents in the Broughton Archipelago has been developed [69] and efforts to improve its realism are ongoing [70]. Further work is planned to couple the hydrodynamic and population-dynamic models in order to understand the effects of sea louse dispersal on sea louse dynamics in the Broughton Archipelago.

The present study advances the science of sea louse (*L. salmonis*) population dynamics in two ways: first, by accommodating explicitly the effects of temperature and salinity on sea louse population growth rates [47], and second, by increasing the spatial and temporal scope of data used to model sea louse population dynamics in the Broughton Archipelago by one order of magnitude over previous studies [47]. Nevertheless, our analysis is subject to several limitations. Data are monthly aggregates and offer only a coarse resolution of the sea louse population dynamics. Although the data represent seven years of sea louse abundance monitoring on 25 salmon farms and correspond to 122 stock-harvest cycles on farms, missing values reduce the usable data to 51 pre-treatment and 32 post-treatment stock-harvest cycles.

While suggestive of timing strategies that could be applied for wild fish conservation management in other regions, the results of the present study are not easily generalizable. British Columbia is atypical among salmon farming regions by virtue of the lack of evidence for sea louse resistance to SLICE® in British Columbia. In jurisdictions where sea louse resistance to SLICE® is established or growing, the timing of SLICE® treatment relative to wild fish migrations may be of reduced value or immaterial to wild fish conservation. Nevertheless, within British Columbia in regions with seasonal temperature and salinity profiles similar to the Broughton Archipelago, there is reason to believe that wild Pacific salmon may benefit generally from winter treatment of salmon farms by SLICE®.

In order to inform adequately the ongoing conservation management of wild Pacific salmon, additional information is required about the long-term efficacy of SLICE® in British Columbia. SLICE® is the only chemotherapeutant that is licenced for use on salmon farms in Pacific Canada [35], and reliance on a single paraciticide can lead to an evolved resistance in parasites and a reduced efficacy [71], as has been observed in Scotland [40], Atlantic Canada [41], [42], Ireland, and Norway [35]. Environmental concern over the trace presence of emamectin benzoate in sediment and the tissue of non-target crustaceans [72], [73] warrants further study. Nevertheless, our results show that judicious timing for the application of SLICE® on salmon farms in the Broughton Archipelago has the potential to reduce the exposure of juvenile wild pink, chum, and coho salmon to parasitic sea lice. Thus, parasite management on finfish farms to reduce infection pressure on wild fish can help to conserve biodiversity and support wild capture fisheries.

## Supporting Information

Animation S1 SLICE® treatments on Atlantic salmon farms in the Broughton Archipelago, British Columbia, Canada from November 1999 to December 2009 (Marty et al. 2010 PNAS). SLICE® treatment is indicated by a red circle at the salmon farm location. Untreated farms are shown by green circles, and farms fade from red to green over the four month efficacy period of SLICE® treatments (see main text). Fallowed farms (i.e., no Atlantic salmon in net pens) are indicated by beige circles with an ‘x’. Data that were used in the analysis of sea louse population dynamics before and after treatments (see main text) are circled in thick black. The period of juvenile wild salmon migration is indicated by purple arrows along approximate migration routes.
(ZIP)
