# Supplementary figures and images for: Modeling Parasite Dynamics on Farmed Salmon for Precautionary Conservation Management of Wild Salmon (part 1 of 2)
Source: PLoS One. 2013 Apr 5;8(4):e60096. doi: 10.1371/journal.pone.0060096 (PMC3618109; doi:10.1371/journal.pone.0060096)

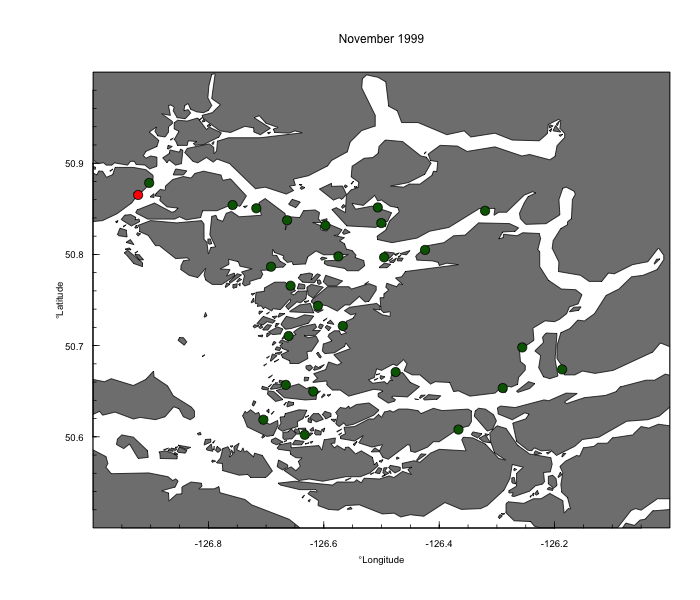

Supplement: Animation S1 — SLICE® treatments on Atlantic salmon farms in the Broughton Archipelago, British Columbia, Canada from November 1999 to December 2009 (Marty et al. 2010 PNAS). SLICE® treatment is indicated by a red circle at the salmon farm location. Untreated farms are shown by green circles, and farms fade from red to green over the four month efficacy period of SLICE® treatments (see main text). Fallowed farms (i.e., no Atlantic salmon in net pens) are indicated by beige circles with an ‘x’. Data that were used in the analysis of sea louse population dynamics before and after treatments (see main text) are circled in thick black. The period of juvenile wild salmon migration is indicated by purple arrows along approximate migration routes. (ZIP) [file pone.0060096.s001.zip › Animation S1/HTML_plot/BroughtonSLICE1.png]

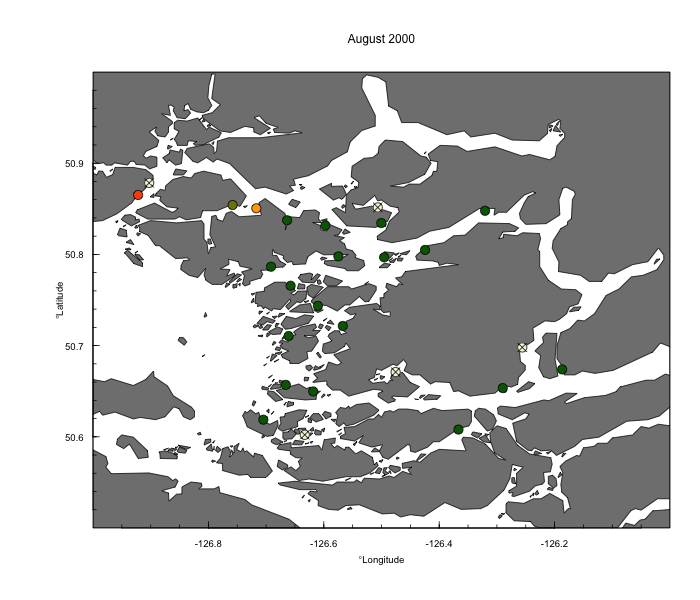

Supplement: Animation S1 — SLICE® treatments on Atlantic salmon farms in the Broughton Archipelago, British Columbia, Canada from November 1999 to December 2009 (Marty et al. 2010 PNAS). SLICE® treatment is indicated by a red circle at the salmon farm location. Untreated farms are shown by green circles, and farms fade from red to green over the four month efficacy period of SLICE® treatments (see main text). Fallowed farms (i.e., no Atlantic salmon in net pens) are indicated by beige circles with an ‘x’. Data that were used in the analysis of sea louse population dynamics before and after treatments (see main text) are circled in thick black. The period of juvenile wild salmon migration is indicated by purple arrows along approximate migration routes. (ZIP) [file pone.0060096.s001.zip › Animation S1/HTML_plot/BroughtonSLICE10.png]

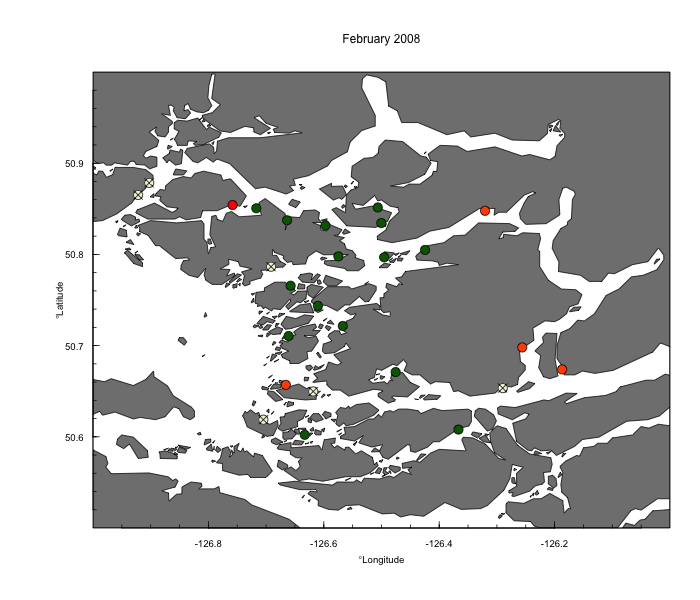

Supplement: Animation S1 — SLICE® treatments on Atlantic salmon farms in the Broughton Archipelago, British Columbia, Canada from November 1999 to December 2009 (Marty et al. 2010 PNAS). SLICE® treatment is indicated by a red circle at the salmon farm location. Untreated farms are shown by green circles, and farms fade from red to green over the four month efficacy period of SLICE® treatments (see main text). Fallowed farms (i.e., no Atlantic salmon in net pens) are indicated by beige circles with an ‘x’. Data that were used in the analysis of sea louse population dynamics before and after treatments (see main text) are circled in thick black. The period of juvenile wild salmon migration is indicated by purple arrows along approximate migration routes. (ZIP) [file pone.0060096.s001.zip › Animation S1/HTML_plot/BroughtonSLICE100.png]

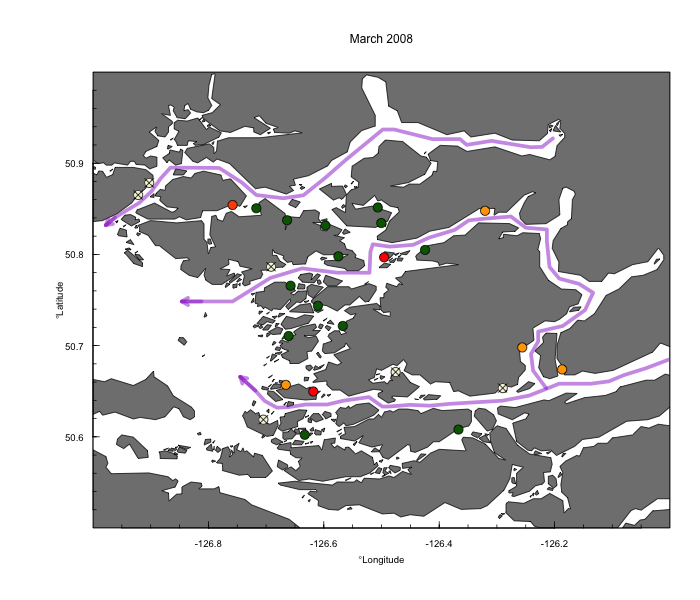

Supplement: Animation S1 — SLICE® treatments on Atlantic salmon farms in the Broughton Archipelago, British Columbia, Canada from November 1999 to December 2009 (Marty et al. 2010 PNAS). SLICE® treatment is indicated by a red circle at the salmon farm location. Untreated farms are shown by green circles, and farms fade from red to green over the four month efficacy period of SLICE® treatments (see main text). Fallowed farms (i.e., no Atlantic salmon in net pens) are indicated by beige circles with an ‘x’. Data that were used in the analysis of sea louse population dynamics before and after treatments (see main text) are circled in thick black. The period of juvenile wild salmon migration is indicated by purple arrows along approximate migration routes. (ZIP) [file pone.0060096.s001.zip › Animation S1/HTML_plot/BroughtonSLICE101.png]

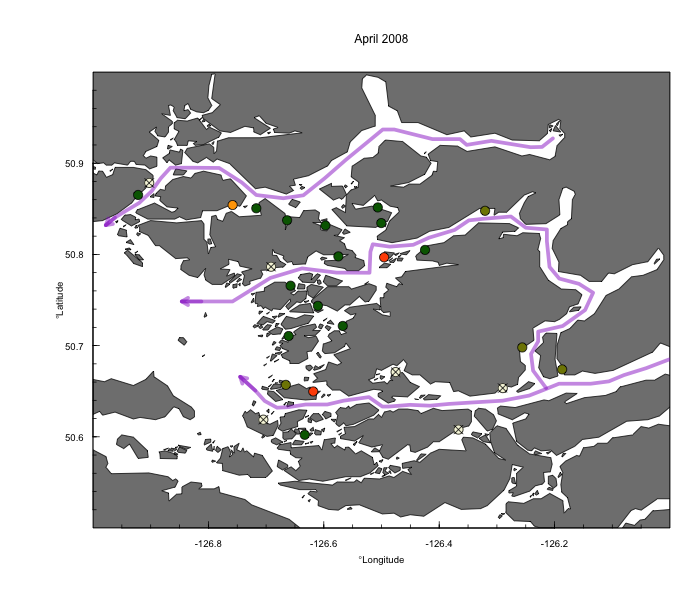

Supplement: Animation S1 — SLICE® treatments on Atlantic salmon farms in the Broughton Archipelago, British Columbia, Canada from November 1999 to December 2009 (Marty et al. 2010 PNAS). SLICE® treatment is indicated by a red circle at the salmon farm location. Untreated farms are shown by green circles, and farms fade from red to green over the four month efficacy period of SLICE® treatments (see main text). Fallowed farms (i.e., no Atlantic salmon in net pens) are indicated by beige circles with an ‘x’. Data that were used in the analysis of sea louse population dynamics before and after treatments (see main text) are circled in thick black. The period of juvenile wild salmon migration is indicated by purple arrows along approximate migration routes. (ZIP) [file pone.0060096.s001.zip › Animation S1/HTML_plot/BroughtonSLICE102.png]

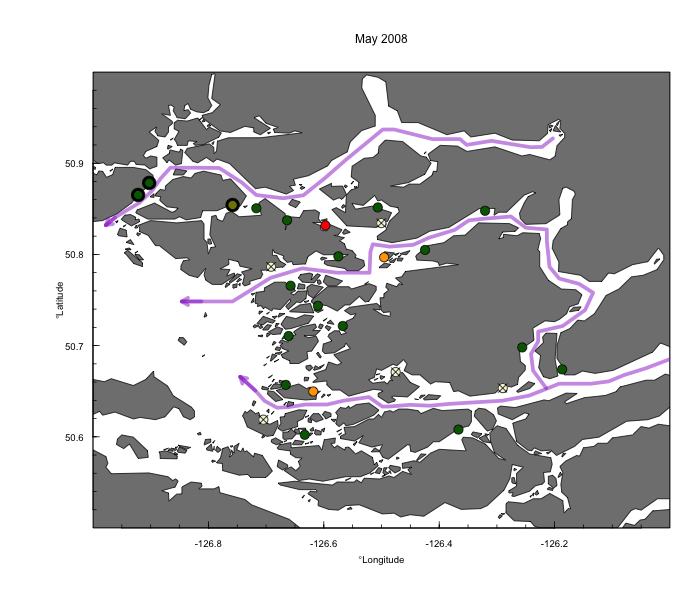

Supplement: Animation S1 — SLICE® treatments on Atlantic salmon farms in the Broughton Archipelago, British Columbia, Canada from November 1999 to December 2009 (Marty et al. 2010 PNAS). SLICE® treatment is indicated by a red circle at the salmon farm location. Untreated farms are shown by green circles, and farms fade from red to green over the four month efficacy period of SLICE® treatments (see main text). Fallowed farms (i.e., no Atlantic salmon in net pens) are indicated by beige circles with an ‘x’. Data that were used in the analysis of sea louse population dynamics before and after treatments (see main text) are circled in thick black. The period of juvenile wild salmon migration is indicated by purple arrows along approximate migration routes. (ZIP) [file pone.0060096.s001.zip › Animation S1/HTML_plot/BroughtonSLICE103.png]

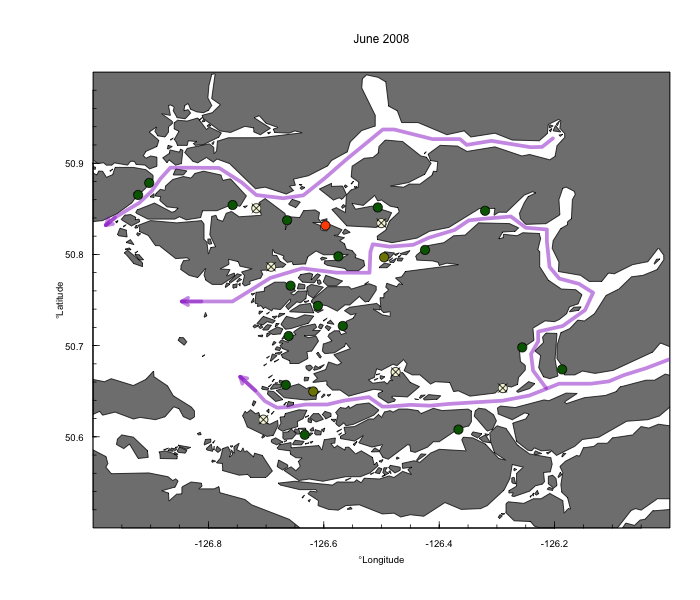

Supplement: Animation S1 — SLICE® treatments on Atlantic salmon farms in the Broughton Archipelago, British Columbia, Canada from November 1999 to December 2009 (Marty et al. 2010 PNAS). SLICE® treatment is indicated by a red circle at the salmon farm location. Untreated farms are shown by green circles, and farms fade from red to green over the four month efficacy period of SLICE® treatments (see main text). Fallowed farms (i.e., no Atlantic salmon in net pens) are indicated by beige circles with an ‘x’. Data that were used in the analysis of sea louse population dynamics before and after treatments (see main text) are circled in thick black. The period of juvenile wild salmon migration is indicated by purple arrows along approximate migration routes. (ZIP) [file pone.0060096.s001.zip › Animation S1/HTML_plot/BroughtonSLICE104.png]

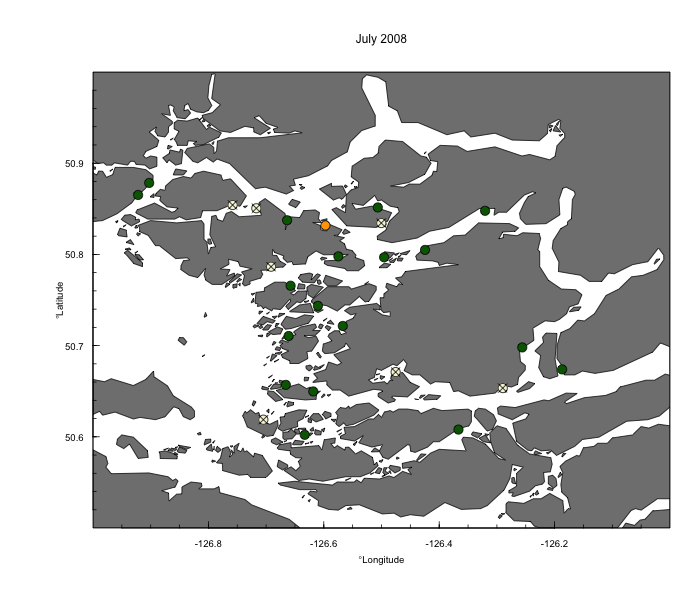

Supplement: Animation S1 — SLICE® treatments on Atlantic salmon farms in the Broughton Archipelago, British Columbia, Canada from November 1999 to December 2009 (Marty et al. 2010 PNAS). SLICE® treatment is indicated by a red circle at the salmon farm location. Untreated farms are shown by green circles, and farms fade from red to green over the four month efficacy period of SLICE® treatments (see main text). Fallowed farms (i.e., no Atlantic salmon in net pens) are indicated by beige circles with an ‘x’. Data that were used in the analysis of sea louse population dynamics before and after treatments (see main text) are circled in thick black. The period of juvenile wild salmon migration is indicated by purple arrows along approximate migration routes. (ZIP) [file pone.0060096.s001.zip › Animation S1/HTML_plot/BroughtonSLICE105.png]

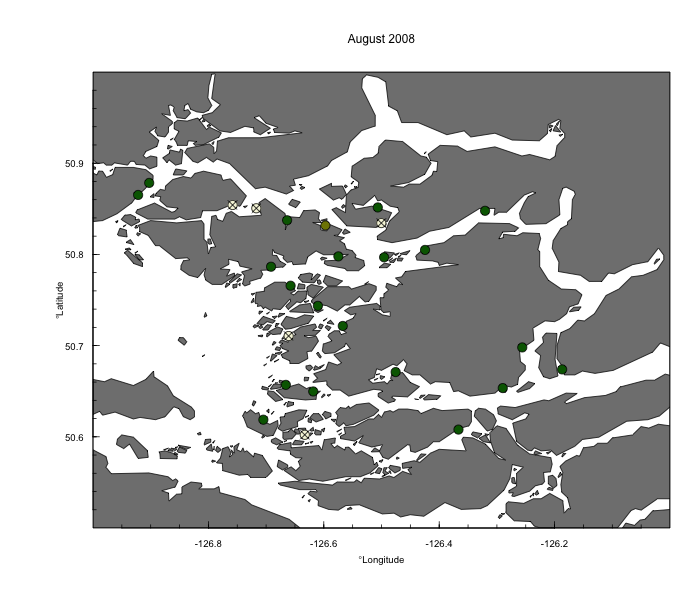

Supplement: Animation S1 — SLICE® treatments on Atlantic salmon farms in the Broughton Archipelago, British Columbia, Canada from November 1999 to December 2009 (Marty et al. 2010 PNAS). SLICE® treatment is indicated by a red circle at the salmon farm location. Untreated farms are shown by green circles, and farms fade from red to green over the four month efficacy period of SLICE® treatments (see main text). Fallowed farms (i.e., no Atlantic salmon in net pens) are indicated by beige circles with an ‘x’. Data that were used in the analysis of sea louse population dynamics before and after treatments (see main text) are circled in thick black. The period of juvenile wild salmon migration is indicated by purple arrows along approximate migration routes. (ZIP) [file pone.0060096.s001.zip › Animation S1/HTML_plot/BroughtonSLICE106.png]

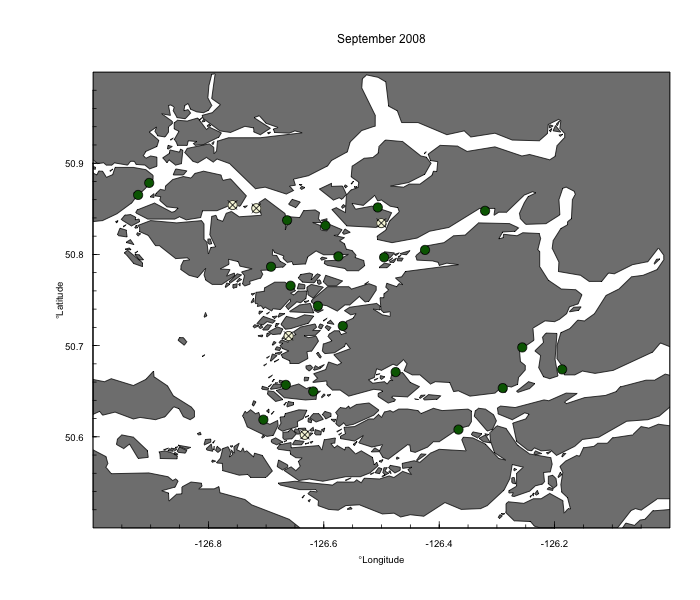

Supplement: Animation S1 — SLICE® treatments on Atlantic salmon farms in the Broughton Archipelago, British Columbia, Canada from November 1999 to December 2009 (Marty et al. 2010 PNAS). SLICE® treatment is indicated by a red circle at the salmon farm location. Untreated farms are shown by green circles, and farms fade from red to green over the four month efficacy period of SLICE® treatments (see main text). Fallowed farms (i.e., no Atlantic salmon in net pens) are indicated by beige circles with an ‘x’. Data that were used in the analysis of sea louse population dynamics before and after treatments (see main text) are circled in thick black. The period of juvenile wild salmon migration is indicated by purple arrows along approximate migration routes. (ZIP) [file pone.0060096.s001.zip › Animation S1/HTML_plot/BroughtonSLICE107.png]

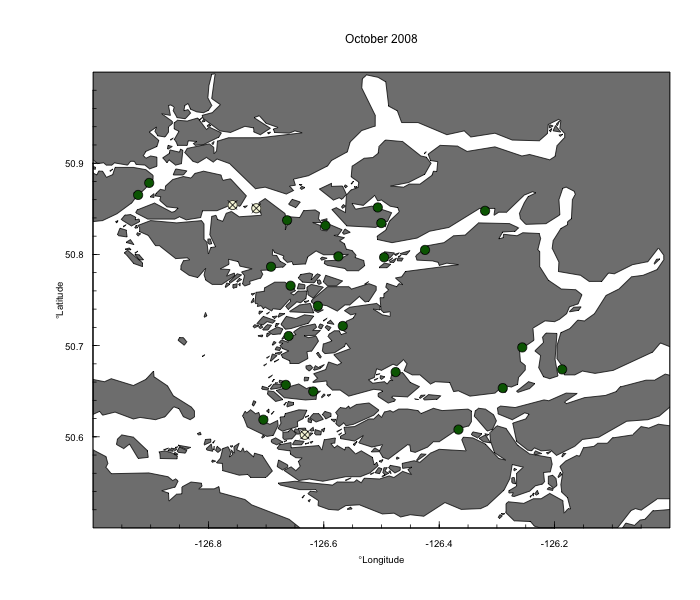

Supplement: Animation S1 — SLICE® treatments on Atlantic salmon farms in the Broughton Archipelago, British Columbia, Canada from November 1999 to December 2009 (Marty et al. 2010 PNAS). SLICE® treatment is indicated by a red circle at the salmon farm location. Untreated farms are shown by green circles, and farms fade from red to green over the four month efficacy period of SLICE® treatments (see main text). Fallowed farms (i.e., no Atlantic salmon in net pens) are indicated by beige circles with an ‘x’. Data that were used in the analysis of sea louse population dynamics before and after treatments (see main text) are circled in thick black. The period of juvenile wild salmon migration is indicated by purple arrows along approximate migration routes. (ZIP) [file pone.0060096.s001.zip › Animation S1/HTML_plot/BroughtonSLICE108.png]

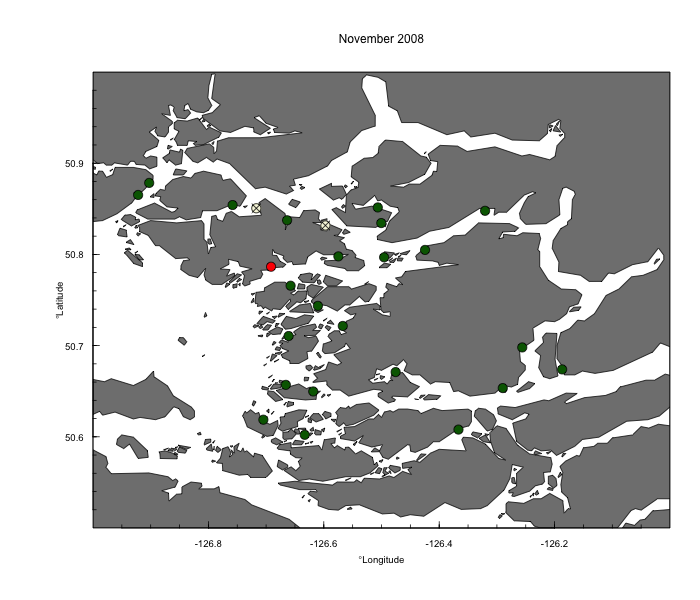

Supplement: Animation S1 — SLICE® treatments on Atlantic salmon farms in the Broughton Archipelago, British Columbia, Canada from November 1999 to December 2009 (Marty et al. 2010 PNAS). SLICE® treatment is indicated by a red circle at the salmon farm location. Untreated farms are shown by green circles, and farms fade from red to green over the four month efficacy period of SLICE® treatments (see main text). Fallowed farms (i.e., no Atlantic salmon in net pens) are indicated by beige circles with an ‘x’. Data that were used in the analysis of sea louse population dynamics before and after treatments (see main text) are circled in thick black. The period of juvenile wild salmon migration is indicated by purple arrows along approximate migration routes. (ZIP) [file pone.0060096.s001.zip › Animation S1/HTML_plot/BroughtonSLICE109.png]

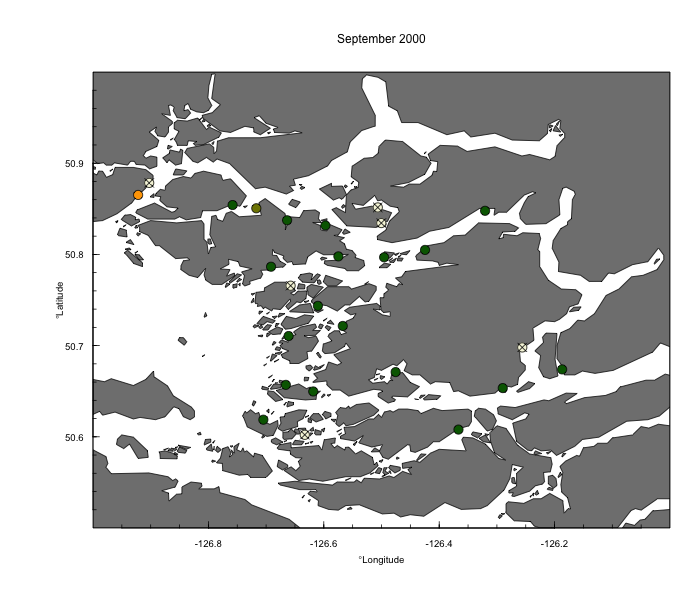

Supplement: Animation S1 — SLICE® treatments on Atlantic salmon farms in the Broughton Archipelago, British Columbia, Canada from November 1999 to December 2009 (Marty et al. 2010 PNAS). SLICE® treatment is indicated by a red circle at the salmon farm location. Untreated farms are shown by green circles, and farms fade from red to green over the four month efficacy period of SLICE® treatments (see main text). Fallowed farms (i.e., no Atlantic salmon in net pens) are indicated by beige circles with an ‘x’. Data that were used in the analysis of sea louse population dynamics before and after treatments (see main text) are circled in thick black. The period of juvenile wild salmon migration is indicated by purple arrows along approximate migration routes. (ZIP) [file pone.0060096.s001.zip › Animation S1/HTML_plot/BroughtonSLICE11.png]

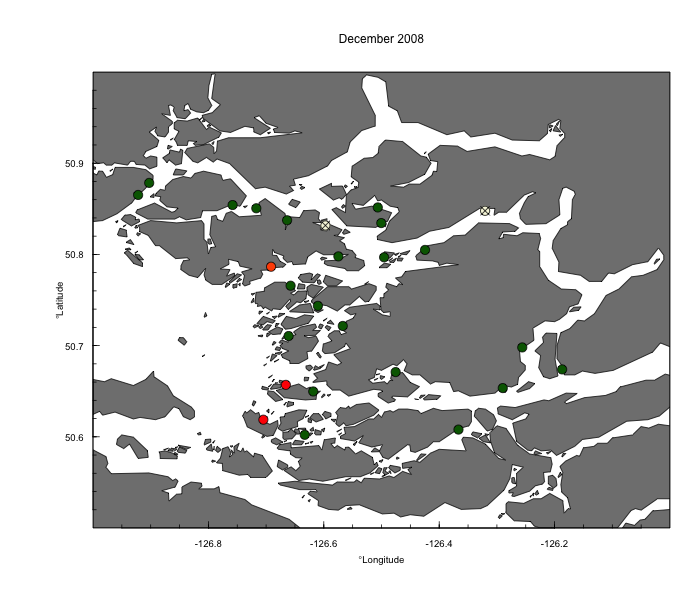

Supplement: Animation S1 — SLICE® treatments on Atlantic salmon farms in the Broughton Archipelago, British Columbia, Canada from November 1999 to December 2009 (Marty et al. 2010 PNAS). SLICE® treatment is indicated by a red circle at the salmon farm location. Untreated farms are shown by green circles, and farms fade from red to green over the four month efficacy period of SLICE® treatments (see main text). Fallowed farms (i.e., no Atlantic salmon in net pens) are indicated by beige circles with an ‘x’. Data that were used in the analysis of sea louse population dynamics before and after treatments (see main text) are circled in thick black. The period of juvenile wild salmon migration is indicated by purple arrows along approximate migration routes. (ZIP) [file pone.0060096.s001.zip › Animation S1/HTML_plot/BroughtonSLICE110.png]

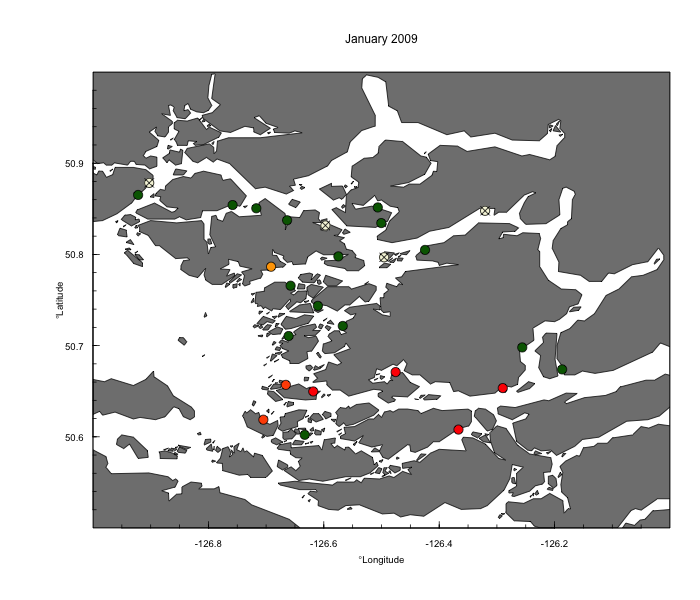

Supplement: Animation S1 — SLICE® treatments on Atlantic salmon farms in the Broughton Archipelago, British Columbia, Canada from November 1999 to December 2009 (Marty et al. 2010 PNAS). SLICE® treatment is indicated by a red circle at the salmon farm location. Untreated farms are shown by green circles, and farms fade from red to green over the four month efficacy period of SLICE® treatments (see main text). Fallowed farms (i.e., no Atlantic salmon in net pens) are indicated by beige circles with an ‘x’. Data that were used in the analysis of sea louse population dynamics before and after treatments (see main text) are circled in thick black. The period of juvenile wild salmon migration is indicated by purple arrows along approximate migration routes. (ZIP) [file pone.0060096.s001.zip › Animation S1/HTML_plot/BroughtonSLICE111.png]

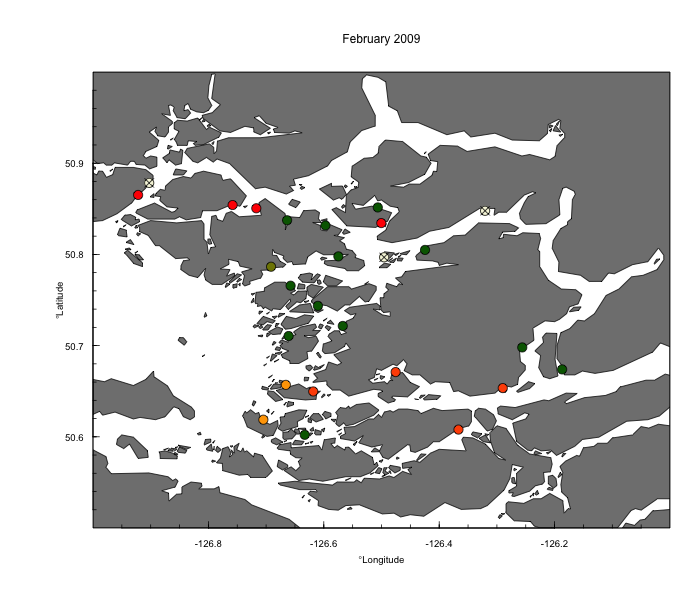

Supplement: Animation S1 — SLICE® treatments on Atlantic salmon farms in the Broughton Archipelago, British Columbia, Canada from November 1999 to December 2009 (Marty et al. 2010 PNAS). SLICE® treatment is indicated by a red circle at the salmon farm location. Untreated farms are shown by green circles, and farms fade from red to green over the four month efficacy period of SLICE® treatments (see main text). Fallowed farms (i.e., no Atlantic salmon in net pens) are indicated by beige circles with an ‘x’. Data that were used in the analysis of sea louse population dynamics before and after treatments (see main text) are circled in thick black. The period of juvenile wild salmon migration is indicated by purple arrows along approximate migration routes. (ZIP) [file pone.0060096.s001.zip › Animation S1/HTML_plot/BroughtonSLICE112.png]

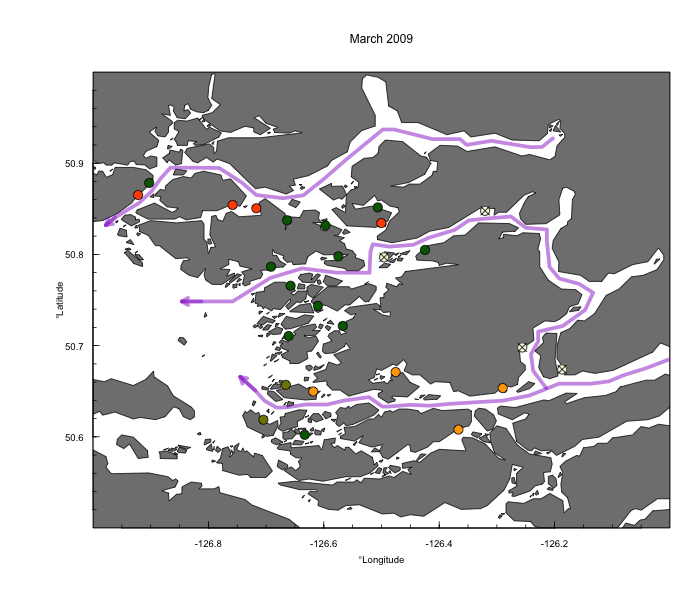

Supplement: Animation S1 — SLICE® treatments on Atlantic salmon farms in the Broughton Archipelago, British Columbia, Canada from November 1999 to December 2009 (Marty et al. 2010 PNAS). SLICE® treatment is indicated by a red circle at the salmon farm location. Untreated farms are shown by green circles, and farms fade from red to green over the four month efficacy period of SLICE® treatments (see main text). Fallowed farms (i.e., no Atlantic salmon in net pens) are indicated by beige circles with an ‘x’. Data that were used in the analysis of sea louse population dynamics before and after treatments (see main text) are circled in thick black. The period of juvenile wild salmon migration is indicated by purple arrows along approximate migration routes. (ZIP) [file pone.0060096.s001.zip › Animation S1/HTML_plot/BroughtonSLICE113.png]

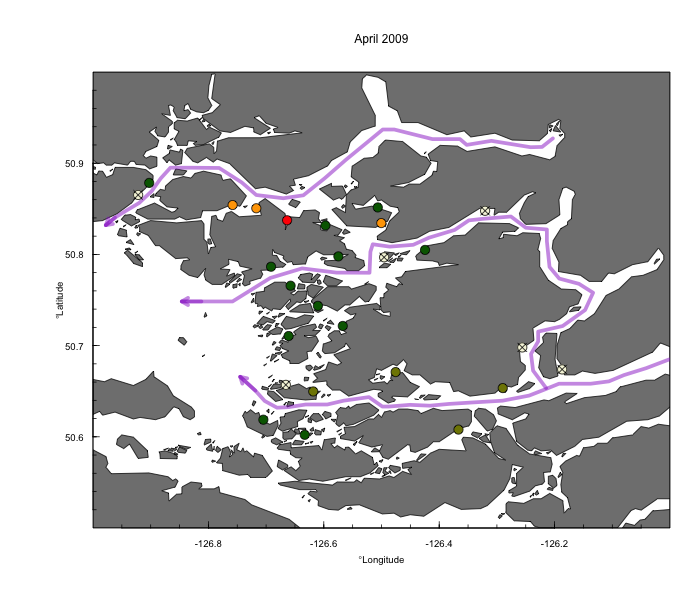

Supplement: Animation S1 — SLICE® treatments on Atlantic salmon farms in the Broughton Archipelago, British Columbia, Canada from November 1999 to December 2009 (Marty et al. 2010 PNAS). SLICE® treatment is indicated by a red circle at the salmon farm location. Untreated farms are shown by green circles, and farms fade from red to green over the four month efficacy period of SLICE® treatments (see main text). Fallowed farms (i.e., no Atlantic salmon in net pens) are indicated by beige circles with an ‘x’. Data that were used in the analysis of sea louse population dynamics before and after treatments (see main text) are circled in thick black. The period of juvenile wild salmon migration is indicated by purple arrows along approximate migration routes. (ZIP) [file pone.0060096.s001.zip › Animation S1/HTML_plot/BroughtonSLICE114.png]

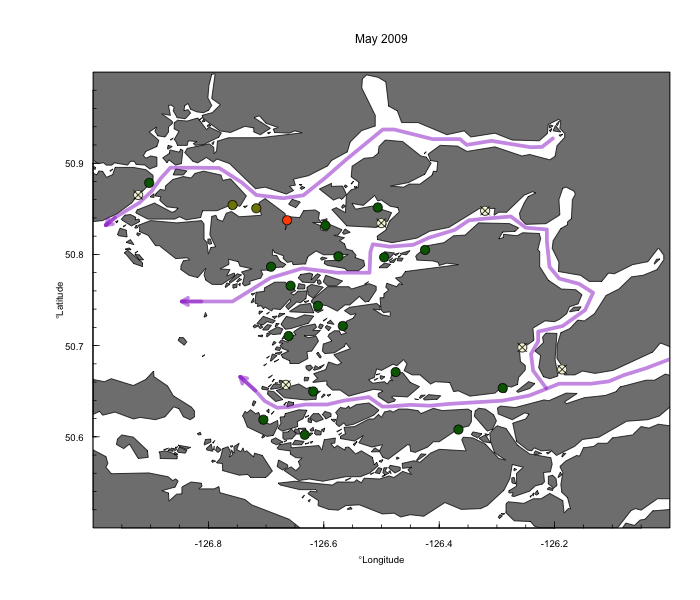

Supplement: Animation S1 — SLICE® treatments on Atlantic salmon farms in the Broughton Archipelago, British Columbia, Canada from November 1999 to December 2009 (Marty et al. 2010 PNAS). SLICE® treatment is indicated by a red circle at the salmon farm location. Untreated farms are shown by green circles, and farms fade from red to green over the four month efficacy period of SLICE® treatments (see main text). Fallowed farms (i.e., no Atlantic salmon in net pens) are indicated by beige circles with an ‘x’. Data that were used in the analysis of sea louse population dynamics before and after treatments (see main text) are circled in thick black. The period of juvenile wild salmon migration is indicated by purple arrows along approximate migration routes. (ZIP) [file pone.0060096.s001.zip › Animation S1/HTML_plot/BroughtonSLICE115.png]

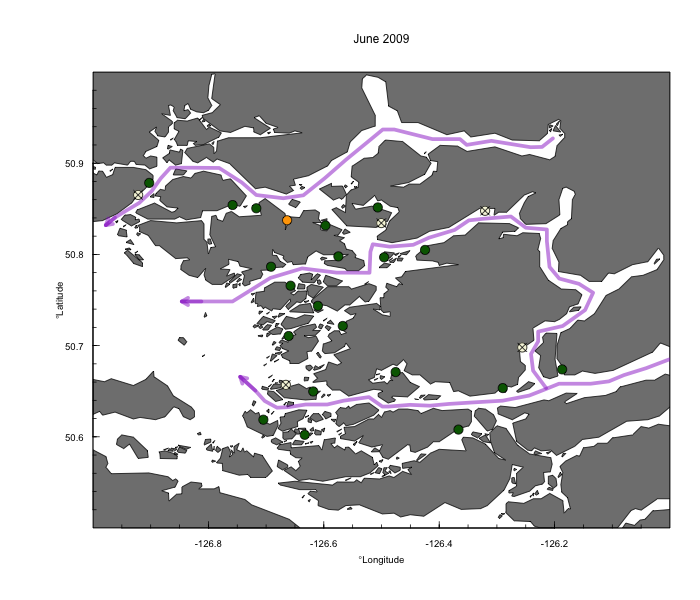

Supplement: Animation S1 — SLICE® treatments on Atlantic salmon farms in the Broughton Archipelago, British Columbia, Canada from November 1999 to December 2009 (Marty et al. 2010 PNAS). SLICE® treatment is indicated by a red circle at the salmon farm location. Untreated farms are shown by green circles, and farms fade from red to green over the four month efficacy period of SLICE® treatments (see main text). Fallowed farms (i.e., no Atlantic salmon in net pens) are indicated by beige circles with an ‘x’. Data that were used in the analysis of sea louse population dynamics before and after treatments (see main text) are circled in thick black. The period of juvenile wild salmon migration is indicated by purple arrows along approximate migration routes. (ZIP) [file pone.0060096.s001.zip › Animation S1/HTML_plot/BroughtonSLICE116.png]

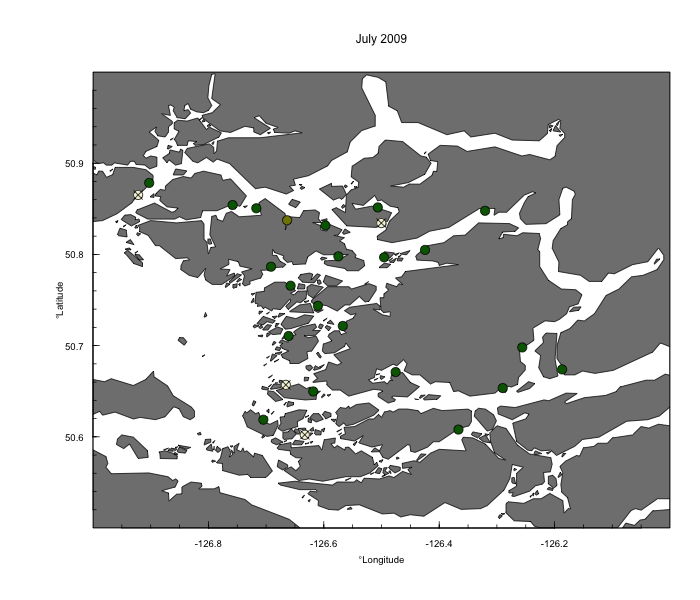

Supplement: Animation S1 — SLICE® treatments on Atlantic salmon farms in the Broughton Archipelago, British Columbia, Canada from November 1999 to December 2009 (Marty et al. 2010 PNAS). SLICE® treatment is indicated by a red circle at the salmon farm location. Untreated farms are shown by green circles, and farms fade from red to green over the four month efficacy period of SLICE® treatments (see main text). Fallowed farms (i.e., no Atlantic salmon in net pens) are indicated by beige circles with an ‘x’. Data that were used in the analysis of sea louse population dynamics before and after treatments (see main text) are circled in thick black. The period of juvenile wild salmon migration is indicated by purple arrows along approximate migration routes. (ZIP) [file pone.0060096.s001.zip › Animation S1/HTML_plot/BroughtonSLICE117.png]

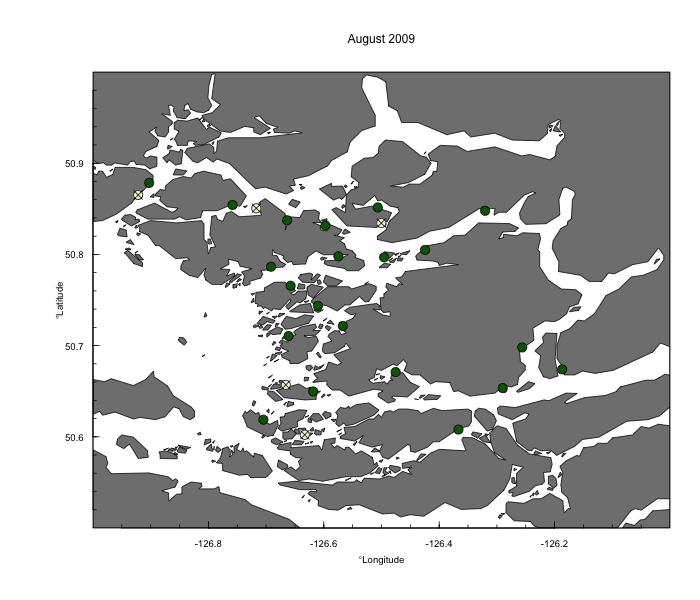

Supplement: Animation S1 — SLICE® treatments on Atlantic salmon farms in the Broughton Archipelago, British Columbia, Canada from November 1999 to December 2009 (Marty et al. 2010 PNAS). SLICE® treatment is indicated by a red circle at the salmon farm location. Untreated farms are shown by green circles, and farms fade from red to green over the four month efficacy period of SLICE® treatments (see main text). Fallowed farms (i.e., no Atlantic salmon in net pens) are indicated by beige circles with an ‘x’. Data that were used in the analysis of sea louse population dynamics before and after treatments (see main text) are circled in thick black. The period of juvenile wild salmon migration is indicated by purple arrows along approximate migration routes. (ZIP) [file pone.0060096.s001.zip › Animation S1/HTML_plot/BroughtonSLICE118.png]

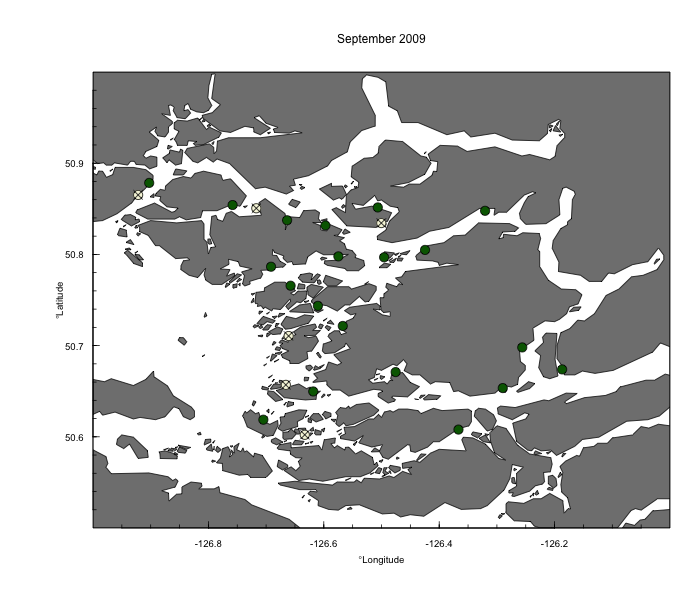

Supplement: Animation S1 — SLICE® treatments on Atlantic salmon farms in the Broughton Archipelago, British Columbia, Canada from November 1999 to December 2009 (Marty et al. 2010 PNAS). SLICE® treatment is indicated by a red circle at the salmon farm location. Untreated farms are shown by green circles, and farms fade from red to green over the four month efficacy period of SLICE® treatments (see main text). Fallowed farms (i.e., no Atlantic salmon in net pens) are indicated by beige circles with an ‘x’. Data that were used in the analysis of sea louse population dynamics before and after treatments (see main text) are circled in thick black. The period of juvenile wild salmon migration is indicated by purple arrows along approximate migration routes. (ZIP) [file pone.0060096.s001.zip › Animation S1/HTML_plot/BroughtonSLICE119.png]

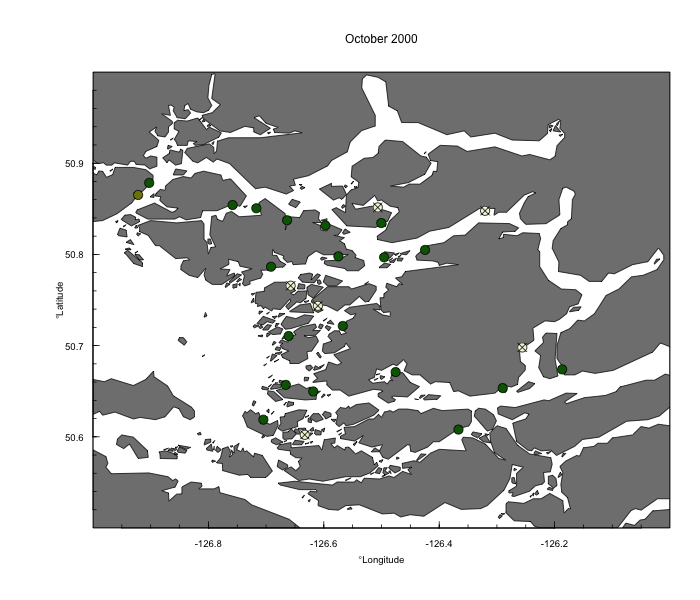

Supplement: Animation S1 — SLICE® treatments on Atlantic salmon farms in the Broughton Archipelago, British Columbia, Canada from November 1999 to December 2009 (Marty et al. 2010 PNAS). SLICE® treatment is indicated by a red circle at the salmon farm location. Untreated farms are shown by green circles, and farms fade from red to green over the four month efficacy period of SLICE® treatments (see main text). Fallowed farms (i.e., no Atlantic salmon in net pens) are indicated by beige circles with an ‘x’. Data that were used in the analysis of sea louse population dynamics before and after treatments (see main text) are circled in thick black. The period of juvenile wild salmon migration is indicated by purple arrows along approximate migration routes. (ZIP) [file pone.0060096.s001.zip › Animation S1/HTML_plot/BroughtonSLICE12.png]

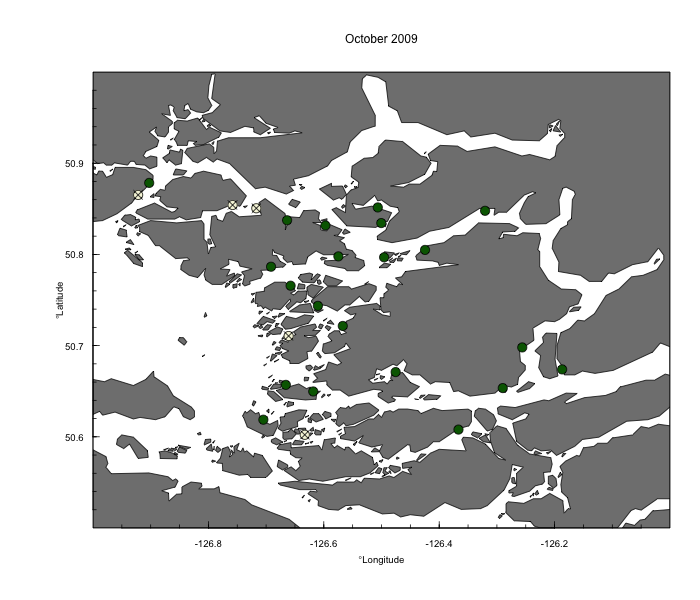

Supplement: Animation S1 — SLICE® treatments on Atlantic salmon farms in the Broughton Archipelago, British Columbia, Canada from November 1999 to December 2009 (Marty et al. 2010 PNAS). SLICE® treatment is indicated by a red circle at the salmon farm location. Untreated farms are shown by green circles, and farms fade from red to green over the four month efficacy period of SLICE® treatments (see main text). Fallowed farms (i.e., no Atlantic salmon in net pens) are indicated by beige circles with an ‘x’. Data that were used in the analysis of sea louse population dynamics before and after treatments (see main text) are circled in thick black. The period of juvenile wild salmon migration is indicated by purple arrows along approximate migration routes. (ZIP) [file pone.0060096.s001.zip › Animation S1/HTML_plot/BroughtonSLICE120.png]

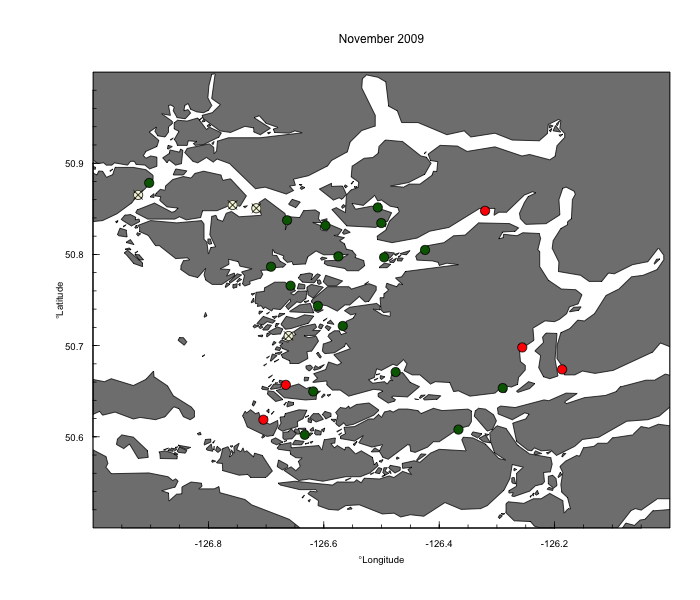

Supplement: Animation S1 — SLICE® treatments on Atlantic salmon farms in the Broughton Archipelago, British Columbia, Canada from November 1999 to December 2009 (Marty et al. 2010 PNAS). SLICE® treatment is indicated by a red circle at the salmon farm location. Untreated farms are shown by green circles, and farms fade from red to green over the four month efficacy period of SLICE® treatments (see main text). Fallowed farms (i.e., no Atlantic salmon in net pens) are indicated by beige circles with an ‘x’. Data that were used in the analysis of sea louse population dynamics before and after treatments (see main text) are circled in thick black. The period of juvenile wild salmon migration is indicated by purple arrows along approximate migration routes. (ZIP) [file pone.0060096.s001.zip › Animation S1/HTML_plot/BroughtonSLICE121.png]

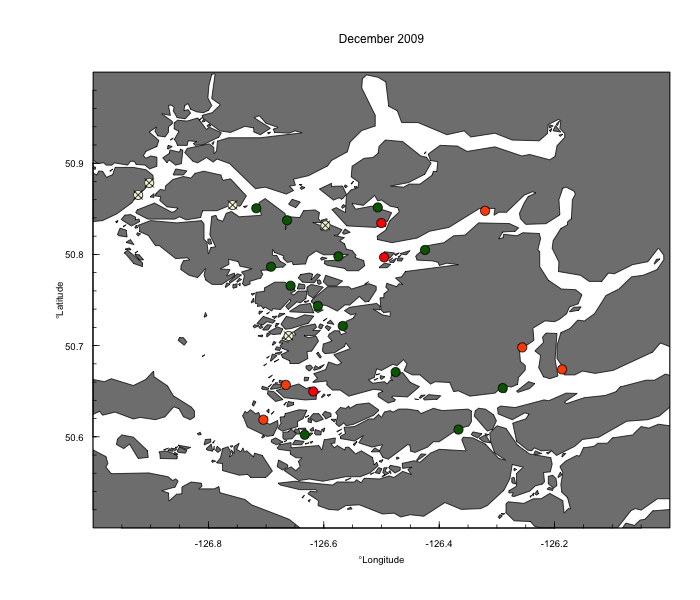

Supplement: Animation S1 — SLICE® treatments on Atlantic salmon farms in the Broughton Archipelago, British Columbia, Canada from November 1999 to December 2009 (Marty et al. 2010 PNAS). SLICE® treatment is indicated by a red circle at the salmon farm location. Untreated farms are shown by green circles, and farms fade from red to green over the four month efficacy period of SLICE® treatments (see main text). Fallowed farms (i.e., no Atlantic salmon in net pens) are indicated by beige circles with an ‘x’. Data that were used in the analysis of sea louse population dynamics before and after treatments (see main text) are circled in thick black. The period of juvenile wild salmon migration is indicated by purple arrows along approximate migration routes. (ZIP) [file pone.0060096.s001.zip › Animation S1/HTML_plot/BroughtonSLICE122.png]

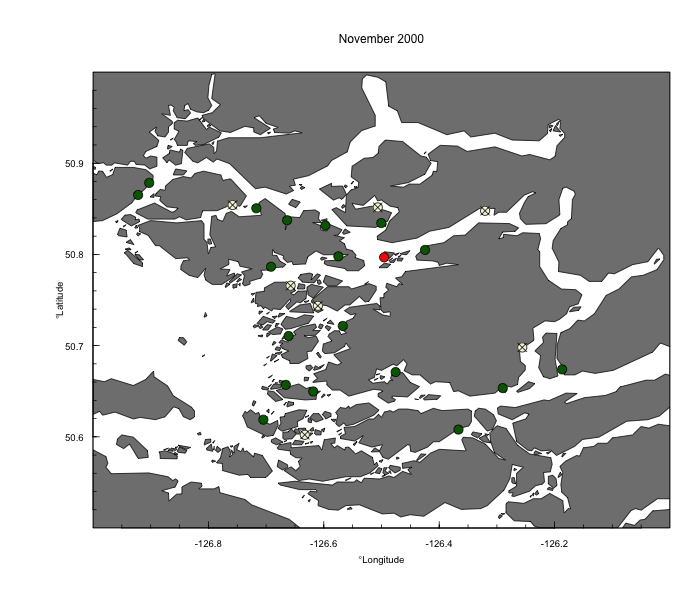

Supplement: Animation S1 — SLICE® treatments on Atlantic salmon farms in the Broughton Archipelago, British Columbia, Canada from November 1999 to December 2009 (Marty et al. 2010 PNAS). SLICE® treatment is indicated by a red circle at the salmon farm location. Untreated farms are shown by green circles, and farms fade from red to green over the four month efficacy period of SLICE® treatments (see main text). Fallowed farms (i.e., no Atlantic salmon in net pens) are indicated by beige circles with an ‘x’. Data that were used in the analysis of sea louse population dynamics before and after treatments (see main text) are circled in thick black. The period of juvenile wild salmon migration is indicated by purple arrows along approximate migration routes. (ZIP) [file pone.0060096.s001.zip › Animation S1/HTML_plot/BroughtonSLICE13.png]

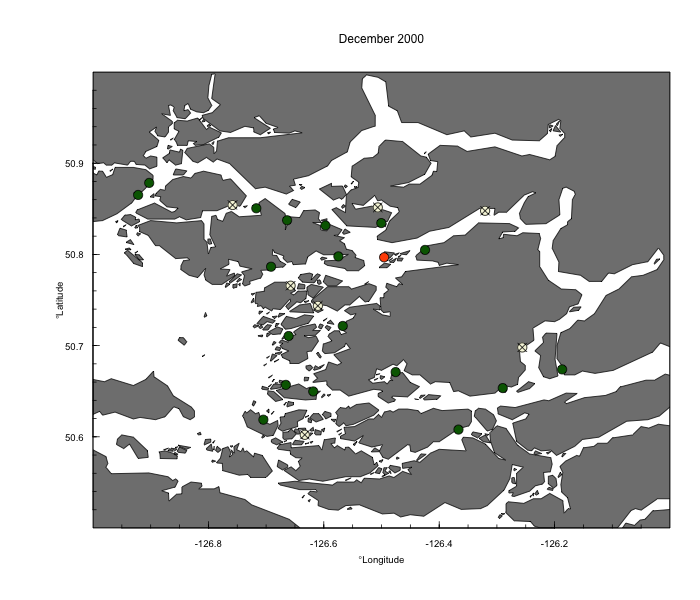

Supplement: Animation S1 — SLICE® treatments on Atlantic salmon farms in the Broughton Archipelago, British Columbia, Canada from November 1999 to December 2009 (Marty et al. 2010 PNAS). SLICE® treatment is indicated by a red circle at the salmon farm location. Untreated farms are shown by green circles, and farms fade from red to green over the four month efficacy period of SLICE® treatments (see main text). Fallowed farms (i.e., no Atlantic salmon in net pens) are indicated by beige circles with an ‘x’. Data that were used in the analysis of sea louse population dynamics before and after treatments (see main text) are circled in thick black. The period of juvenile wild salmon migration is indicated by purple arrows along approximate migration routes. (ZIP) [file pone.0060096.s001.zip › Animation S1/HTML_plot/BroughtonSLICE14.png]

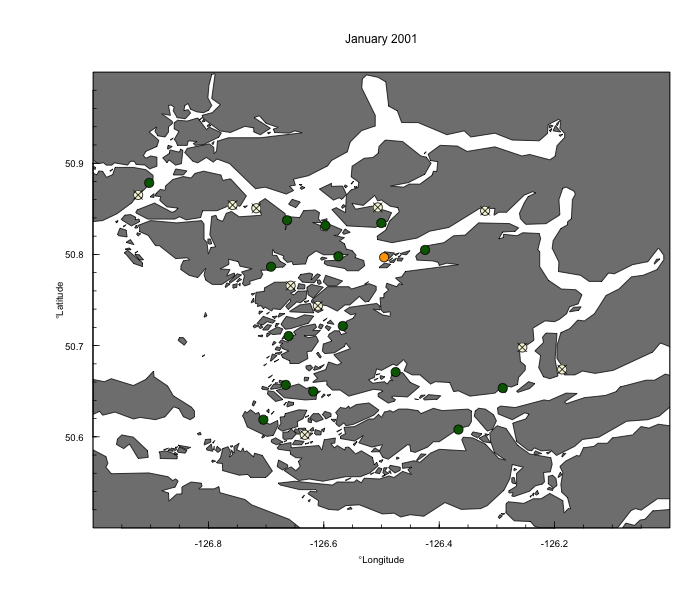

Supplement: Animation S1 — SLICE® treatments on Atlantic salmon farms in the Broughton Archipelago, British Columbia, Canada from November 1999 to December 2009 (Marty et al. 2010 PNAS). SLICE® treatment is indicated by a red circle at the salmon farm location. Untreated farms are shown by green circles, and farms fade from red to green over the four month efficacy period of SLICE® treatments (see main text). Fallowed farms (i.e., no Atlantic salmon in net pens) are indicated by beige circles with an ‘x’. Data that were used in the analysis of sea louse population dynamics before and after treatments (see main text) are circled in thick black. The period of juvenile wild salmon migration is indicated by purple arrows along approximate migration routes. (ZIP) [file pone.0060096.s001.zip › Animation S1/HTML_plot/BroughtonSLICE15.png]

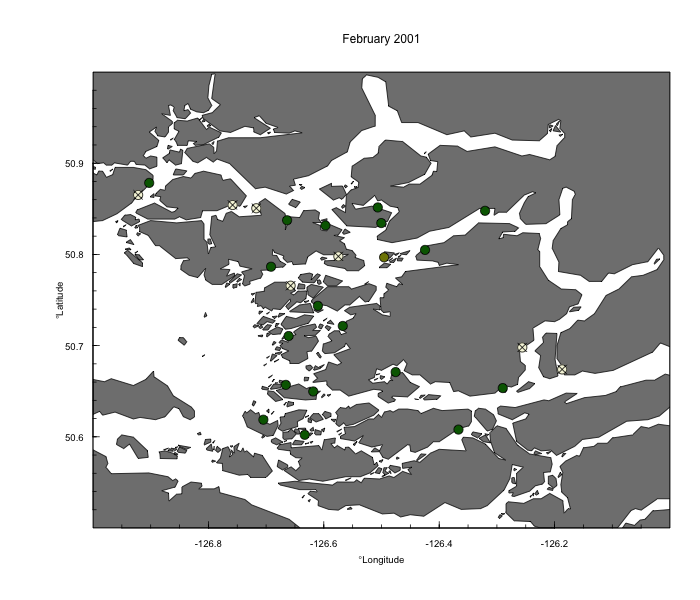

Supplement: Animation S1 — SLICE® treatments on Atlantic salmon farms in the Broughton Archipelago, British Columbia, Canada from November 1999 to December 2009 (Marty et al. 2010 PNAS). SLICE® treatment is indicated by a red circle at the salmon farm location. Untreated farms are shown by green circles, and farms fade from red to green over the four month efficacy period of SLICE® treatments (see main text). Fallowed farms (i.e., no Atlantic salmon in net pens) are indicated by beige circles with an ‘x’. Data that were used in the analysis of sea louse population dynamics before and after treatments (see main text) are circled in thick black. The period of juvenile wild salmon migration is indicated by purple arrows along approximate migration routes. (ZIP) [file pone.0060096.s001.zip › Animation S1/HTML_plot/BroughtonSLICE16.png]

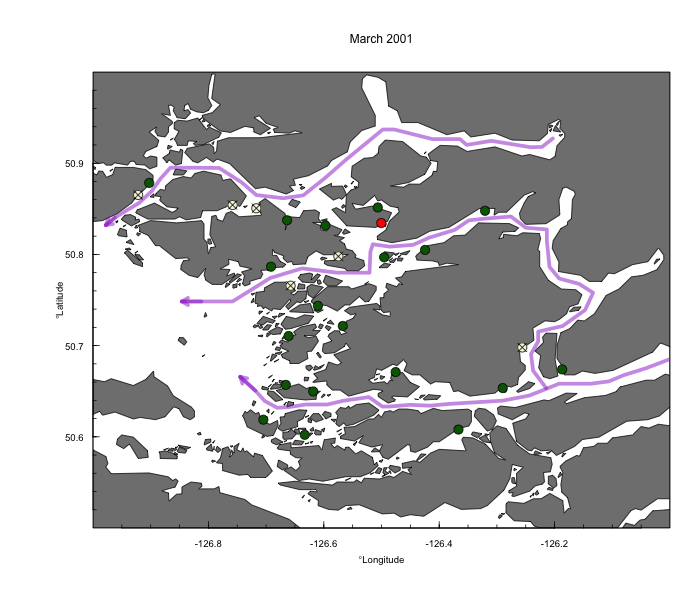

Supplement: Animation S1 — SLICE® treatments on Atlantic salmon farms in the Broughton Archipelago, British Columbia, Canada from November 1999 to December 2009 (Marty et al. 2010 PNAS). SLICE® treatment is indicated by a red circle at the salmon farm location. Untreated farms are shown by green circles, and farms fade from red to green over the four month efficacy period of SLICE® treatments (see main text). Fallowed farms (i.e., no Atlantic salmon in net pens) are indicated by beige circles with an ‘x’. Data that were used in the analysis of sea louse population dynamics before and after treatments (see main text) are circled in thick black. The period of juvenile wild salmon migration is indicated by purple arrows along approximate migration routes. (ZIP) [file pone.0060096.s001.zip › Animation S1/HTML_plot/BroughtonSLICE17.png]

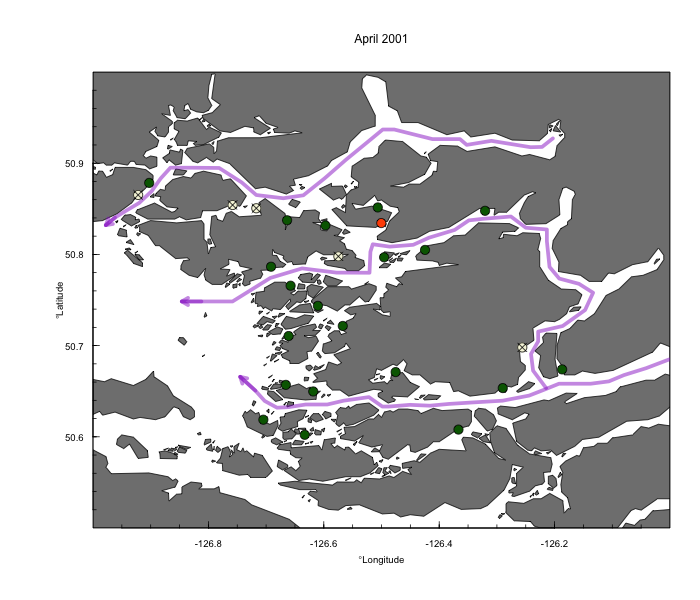

Supplement: Animation S1 — SLICE® treatments on Atlantic salmon farms in the Broughton Archipelago, British Columbia, Canada from November 1999 to December 2009 (Marty et al. 2010 PNAS). SLICE® treatment is indicated by a red circle at the salmon farm location. Untreated farms are shown by green circles, and farms fade from red to green over the four month efficacy period of SLICE® treatments (see main text). Fallowed farms (i.e., no Atlantic salmon in net pens) are indicated by beige circles with an ‘x’. Data that were used in the analysis of sea louse population dynamics before and after treatments (see main text) are circled in thick black. The period of juvenile wild salmon migration is indicated by purple arrows along approximate migration routes. (ZIP) [file pone.0060096.s001.zip › Animation S1/HTML_plot/BroughtonSLICE18.png]

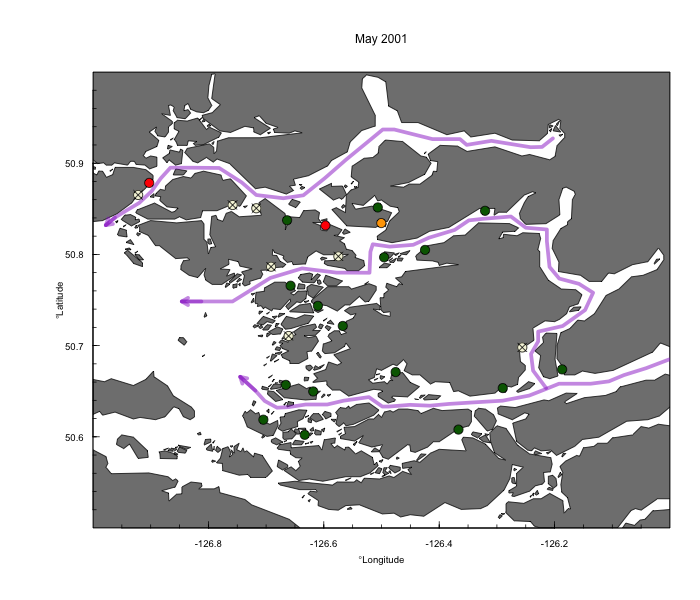

Supplement: Animation S1 — SLICE® treatments on Atlantic salmon farms in the Broughton Archipelago, British Columbia, Canada from November 1999 to December 2009 (Marty et al. 2010 PNAS). SLICE® treatment is indicated by a red circle at the salmon farm location. Untreated farms are shown by green circles, and farms fade from red to green over the four month efficacy period of SLICE® treatments (see main text). Fallowed farms (i.e., no Atlantic salmon in net pens) are indicated by beige circles with an ‘x’. Data that were used in the analysis of sea louse population dynamics before and after treatments (see main text) are circled in thick black. The period of juvenile wild salmon migration is indicated by purple arrows along approximate migration routes. (ZIP) [file pone.0060096.s001.zip › Animation S1/HTML_plot/BroughtonSLICE19.png]

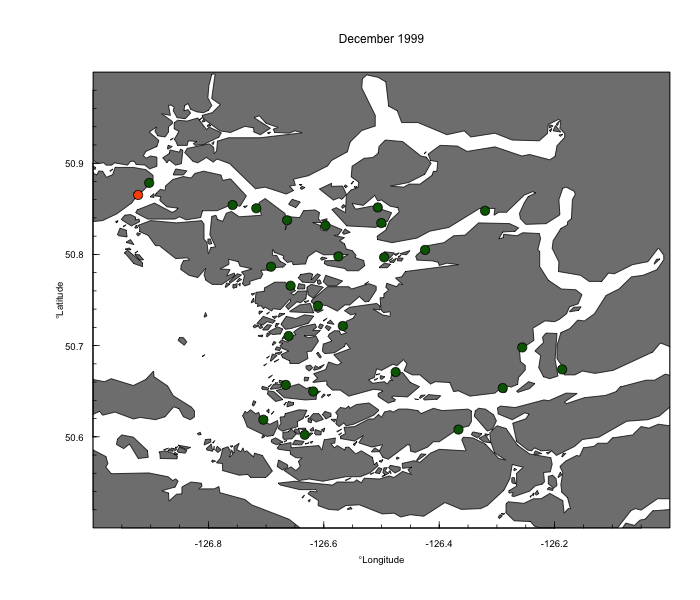

Supplement: Animation S1 — SLICE® treatments on Atlantic salmon farms in the Broughton Archipelago, British Columbia, Canada from November 1999 to December 2009 (Marty et al. 2010 PNAS). SLICE® treatment is indicated by a red circle at the salmon farm location. Untreated farms are shown by green circles, and farms fade from red to green over the four month efficacy period of SLICE® treatments (see main text). Fallowed farms (i.e., no Atlantic salmon in net pens) are indicated by beige circles with an ‘x’. Data that were used in the analysis of sea louse population dynamics before and after treatments (see main text) are circled in thick black. The period of juvenile wild salmon migration is indicated by purple arrows along approximate migration routes. (ZIP) [file pone.0060096.s001.zip › Animation S1/HTML_plot/BroughtonSLICE2.png]

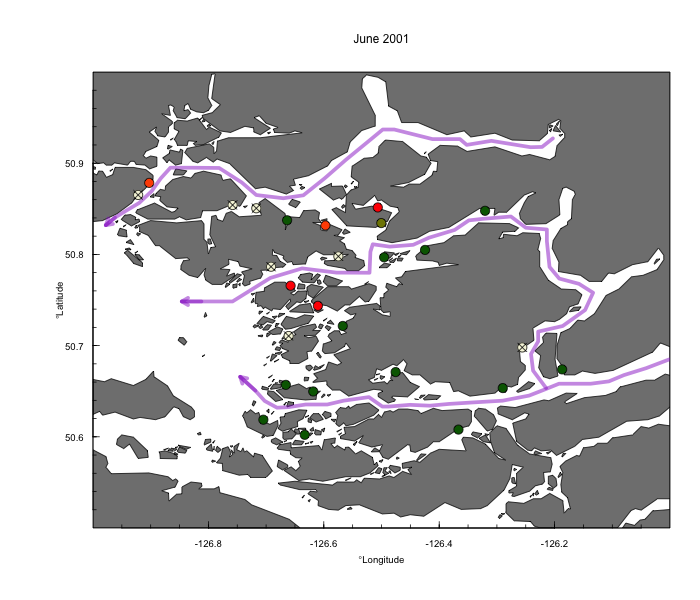

Supplement: Animation S1 — SLICE® treatments on Atlantic salmon farms in the Broughton Archipelago, British Columbia, Canada from November 1999 to December 2009 (Marty et al. 2010 PNAS). SLICE® treatment is indicated by a red circle at the salmon farm location. Untreated farms are shown by green circles, and farms fade from red to green over the four month efficacy period of SLICE® treatments (see main text). Fallowed farms (i.e., no Atlantic salmon in net pens) are indicated by beige circles with an ‘x’. Data that were used in the analysis of sea louse population dynamics before and after treatments (see main text) are circled in thick black. The period of juvenile wild salmon migration is indicated by purple arrows along approximate migration routes. (ZIP) [file pone.0060096.s001.zip › Animation S1/HTML_plot/BroughtonSLICE20.png]

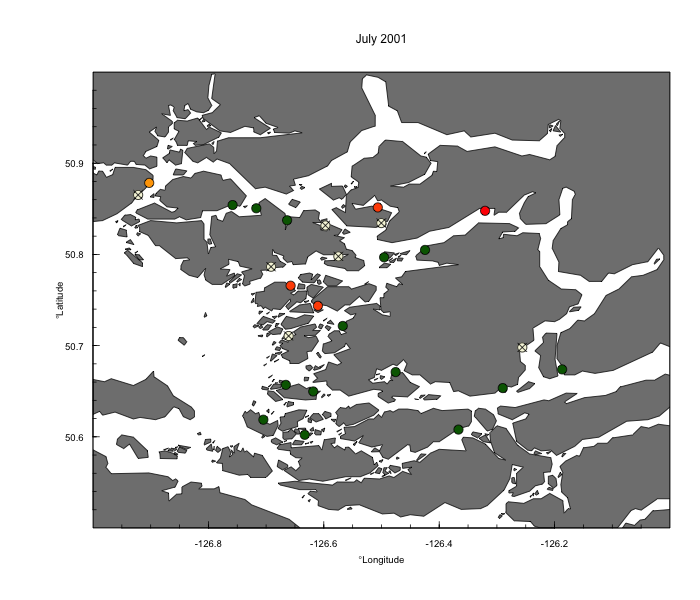

Supplement: Animation S1 — SLICE® treatments on Atlantic salmon farms in the Broughton Archipelago, British Columbia, Canada from November 1999 to December 2009 (Marty et al. 2010 PNAS). SLICE® treatment is indicated by a red circle at the salmon farm location. Untreated farms are shown by green circles, and farms fade from red to green over the four month efficacy period of SLICE® treatments (see main text). Fallowed farms (i.e., no Atlantic salmon in net pens) are indicated by beige circles with an ‘x’. Data that were used in the analysis of sea louse population dynamics before and after treatments (see main text) are circled in thick black. The period of juvenile wild salmon migration is indicated by purple arrows along approximate migration routes. (ZIP) [file pone.0060096.s001.zip › Animation S1/HTML_plot/BroughtonSLICE21.png]

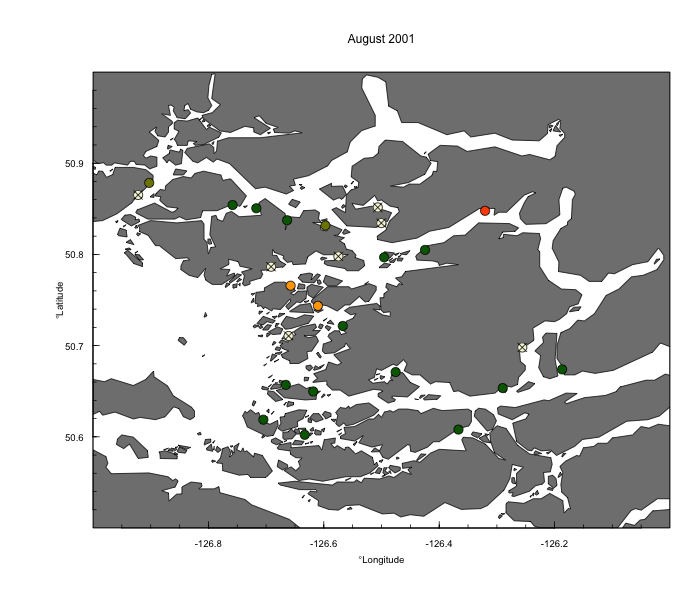

Supplement: Animation S1 — SLICE® treatments on Atlantic salmon farms in the Broughton Archipelago, British Columbia, Canada from November 1999 to December 2009 (Marty et al. 2010 PNAS). SLICE® treatment is indicated by a red circle at the salmon farm location. Untreated farms are shown by green circles, and farms fade from red to green over the four month efficacy period of SLICE® treatments (see main text). Fallowed farms (i.e., no Atlantic salmon in net pens) are indicated by beige circles with an ‘x’. Data that were used in the analysis of sea louse population dynamics before and after treatments (see main text) are circled in thick black. The period of juvenile wild salmon migration is indicated by purple arrows along approximate migration routes. (ZIP) [file pone.0060096.s001.zip › Animation S1/HTML_plot/BroughtonSLICE22.png]

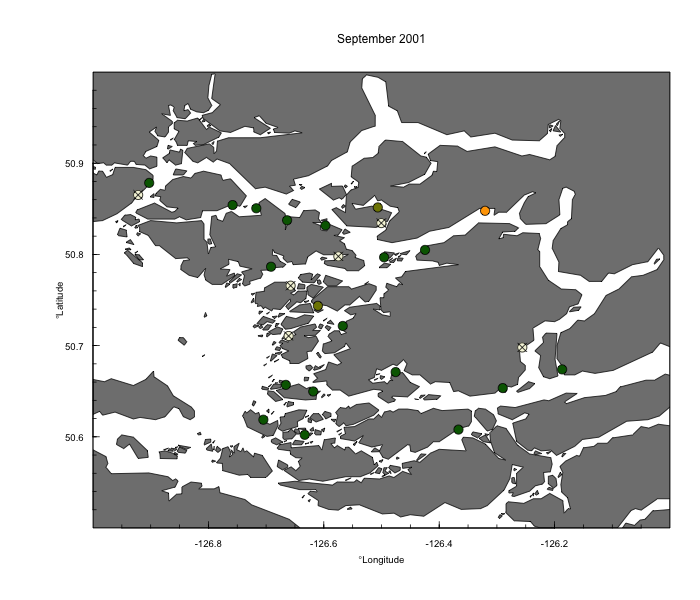

Supplement: Animation S1 — SLICE® treatments on Atlantic salmon farms in the Broughton Archipelago, British Columbia, Canada from November 1999 to December 2009 (Marty et al. 2010 PNAS). SLICE® treatment is indicated by a red circle at the salmon farm location. Untreated farms are shown by green circles, and farms fade from red to green over the four month efficacy period of SLICE® treatments (see main text). Fallowed farms (i.e., no Atlantic salmon in net pens) are indicated by beige circles with an ‘x’. Data that were used in the analysis of sea louse population dynamics before and after treatments (see main text) are circled in thick black. The period of juvenile wild salmon migration is indicated by purple arrows along approximate migration routes. (ZIP) [file pone.0060096.s001.zip › Animation S1/HTML_plot/BroughtonSLICE23.png]

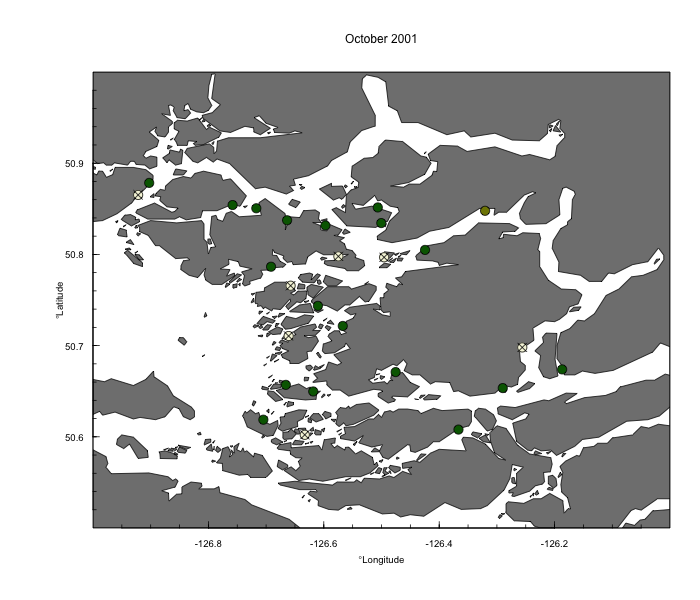

Supplement: Animation S1 — SLICE® treatments on Atlantic salmon farms in the Broughton Archipelago, British Columbia, Canada from November 1999 to December 2009 (Marty et al. 2010 PNAS). SLICE® treatment is indicated by a red circle at the salmon farm location. Untreated farms are shown by green circles, and farms fade from red to green over the four month efficacy period of SLICE® treatments (see main text). Fallowed farms (i.e., no Atlantic salmon in net pens) are indicated by beige circles with an ‘x’. Data that were used in the analysis of sea louse population dynamics before and after treatments (see main text) are circled in thick black. The period of juvenile wild salmon migration is indicated by purple arrows along approximate migration routes. (ZIP) [file pone.0060096.s001.zip › Animation S1/HTML_plot/BroughtonSLICE24.png]

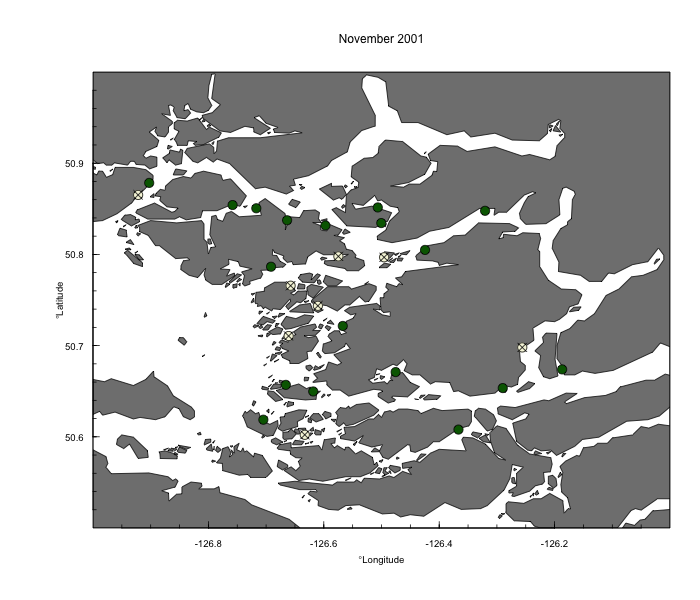

Supplement: Animation S1 — SLICE® treatments on Atlantic salmon farms in the Broughton Archipelago, British Columbia, Canada from November 1999 to December 2009 (Marty et al. 2010 PNAS). SLICE® treatment is indicated by a red circle at the salmon farm location. Untreated farms are shown by green circles, and farms fade from red to green over the four month efficacy period of SLICE® treatments (see main text). Fallowed farms (i.e., no Atlantic salmon in net pens) are indicated by beige circles with an ‘x’. Data that were used in the analysis of sea louse population dynamics before and after treatments (see main text) are circled in thick black. The period of juvenile wild salmon migration is indicated by purple arrows along approximate migration routes. (ZIP) [file pone.0060096.s001.zip › Animation S1/HTML_plot/BroughtonSLICE25.png]

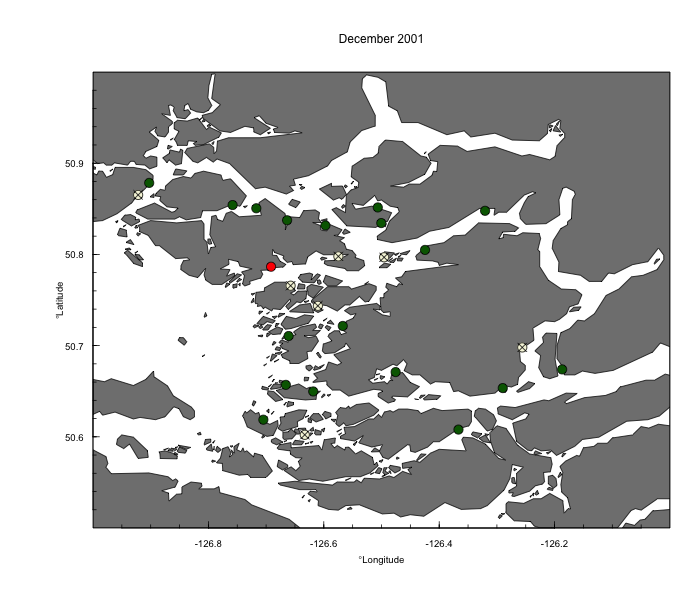

Supplement: Animation S1 — SLICE® treatments on Atlantic salmon farms in the Broughton Archipelago, British Columbia, Canada from November 1999 to December 2009 (Marty et al. 2010 PNAS). SLICE® treatment is indicated by a red circle at the salmon farm location. Untreated farms are shown by green circles, and farms fade from red to green over the four month efficacy period of SLICE® treatments (see main text). Fallowed farms (i.e., no Atlantic salmon in net pens) are indicated by beige circles with an ‘x’. Data that were used in the analysis of sea louse population dynamics before and after treatments (see main text) are circled in thick black. The period of juvenile wild salmon migration is indicated by purple arrows along approximate migration routes. (ZIP) [file pone.0060096.s001.zip › Animation S1/HTML_plot/BroughtonSLICE26.png]

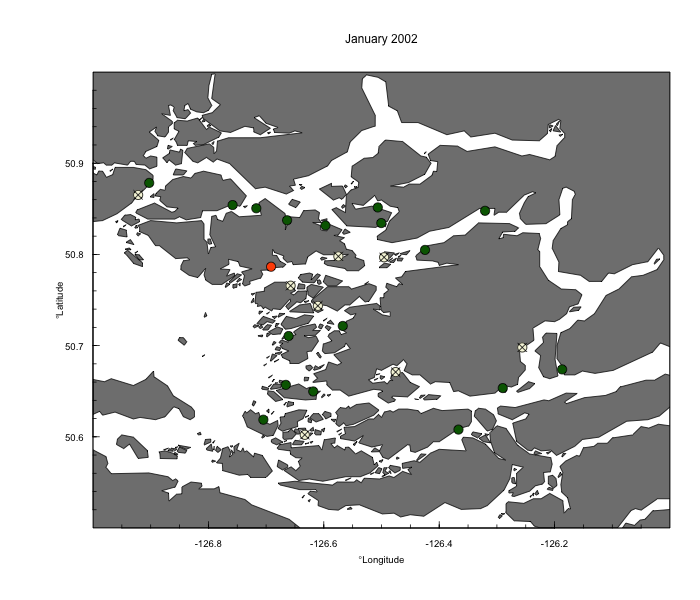

Supplement: Animation S1 — SLICE® treatments on Atlantic salmon farms in the Broughton Archipelago, British Columbia, Canada from November 1999 to December 2009 (Marty et al. 2010 PNAS). SLICE® treatment is indicated by a red circle at the salmon farm location. Untreated farms are shown by green circles, and farms fade from red to green over the four month efficacy period of SLICE® treatments (see main text). Fallowed farms (i.e., no Atlantic salmon in net pens) are indicated by beige circles with an ‘x’. Data that were used in the analysis of sea louse population dynamics before and after treatments (see main text) are circled in thick black. The period of juvenile wild salmon migration is indicated by purple arrows along approximate migration routes. (ZIP) [file pone.0060096.s001.zip › Animation S1/HTML_plot/BroughtonSLICE27.png]

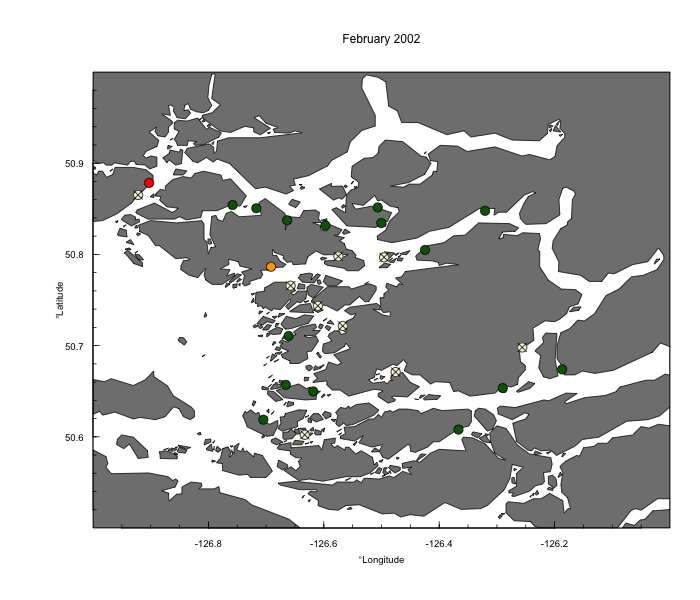

Supplement: Animation S1 — SLICE® treatments on Atlantic salmon farms in the Broughton Archipelago, British Columbia, Canada from November 1999 to December 2009 (Marty et al. 2010 PNAS). SLICE® treatment is indicated by a red circle at the salmon farm location. Untreated farms are shown by green circles, and farms fade from red to green over the four month efficacy period of SLICE® treatments (see main text). Fallowed farms (i.e., no Atlantic salmon in net pens) are indicated by beige circles with an ‘x’. Data that were used in the analysis of sea louse population dynamics before and after treatments (see main text) are circled in thick black. The period of juvenile wild salmon migration is indicated by purple arrows along approximate migration routes. (ZIP) [file pone.0060096.s001.zip › Animation S1/HTML_plot/BroughtonSLICE28.png]

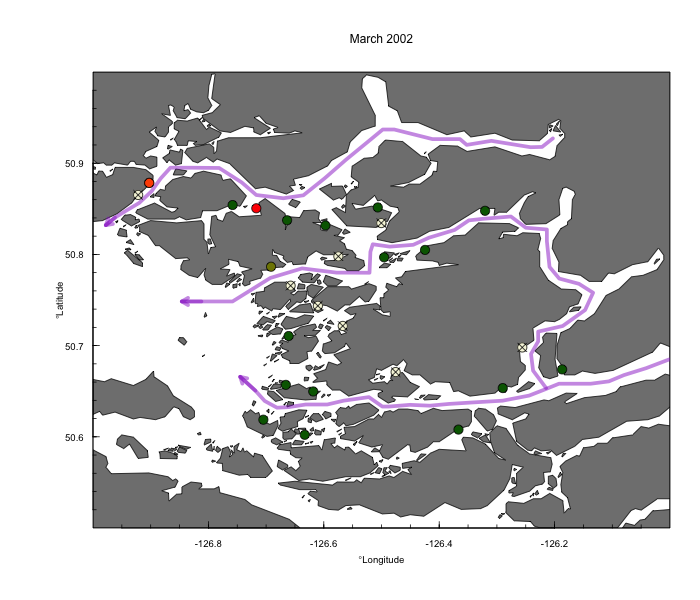

Supplement: Animation S1 — SLICE® treatments on Atlantic salmon farms in the Broughton Archipelago, British Columbia, Canada from November 1999 to December 2009 (Marty et al. 2010 PNAS). SLICE® treatment is indicated by a red circle at the salmon farm location. Untreated farms are shown by green circles, and farms fade from red to green over the four month efficacy period of SLICE® treatments (see main text). Fallowed farms (i.e., no Atlantic salmon in net pens) are indicated by beige circles with an ‘x’. Data that were used in the analysis of sea louse population dynamics before and after treatments (see main text) are circled in thick black. The period of juvenile wild salmon migration is indicated by purple arrows along approximate migration routes. (ZIP) [file pone.0060096.s001.zip › Animation S1/HTML_plot/BroughtonSLICE29.png]

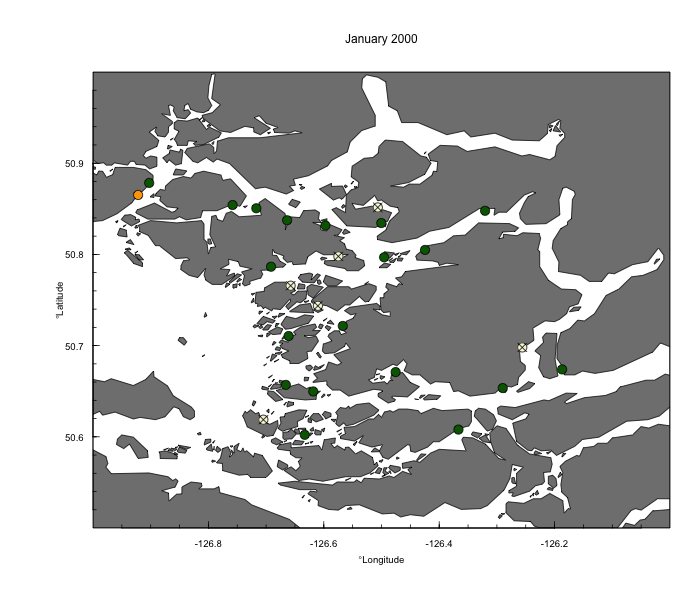

Supplement: Animation S1 — SLICE® treatments on Atlantic salmon farms in the Broughton Archipelago, British Columbia, Canada from November 1999 to December 2009 (Marty et al. 2010 PNAS). SLICE® treatment is indicated by a red circle at the salmon farm location. Untreated farms are shown by green circles, and farms fade from red to green over the four month efficacy period of SLICE® treatments (see main text). Fallowed farms (i.e., no Atlantic salmon in net pens) are indicated by beige circles with an ‘x’. Data that were used in the analysis of sea louse population dynamics before and after treatments (see main text) are circled in thick black. The period of juvenile wild salmon migration is indicated by purple arrows along approximate migration routes. (ZIP) [file pone.0060096.s001.zip › Animation S1/HTML_plot/BroughtonSLICE3.png]

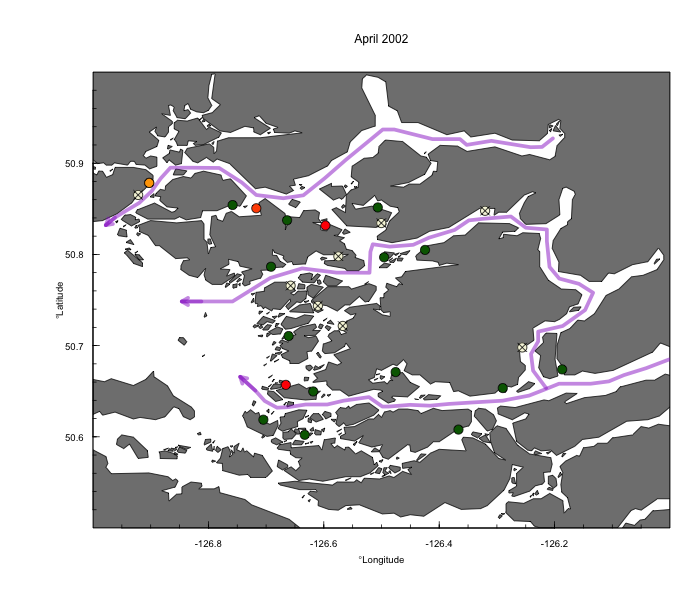

Supplement: Animation S1 — SLICE® treatments on Atlantic salmon farms in the Broughton Archipelago, British Columbia, Canada from November 1999 to December 2009 (Marty et al. 2010 PNAS). SLICE® treatment is indicated by a red circle at the salmon farm location. Untreated farms are shown by green circles, and farms fade from red to green over the four month efficacy period of SLICE® treatments (see main text). Fallowed farms (i.e., no Atlantic salmon in net pens) are indicated by beige circles with an ‘x’. Data that were used in the analysis of sea louse population dynamics before and after treatments (see main text) are circled in thick black. The period of juvenile wild salmon migration is indicated by purple arrows along approximate migration routes. (ZIP) [file pone.0060096.s001.zip › Animation S1/HTML_plot/BroughtonSLICE30.png]

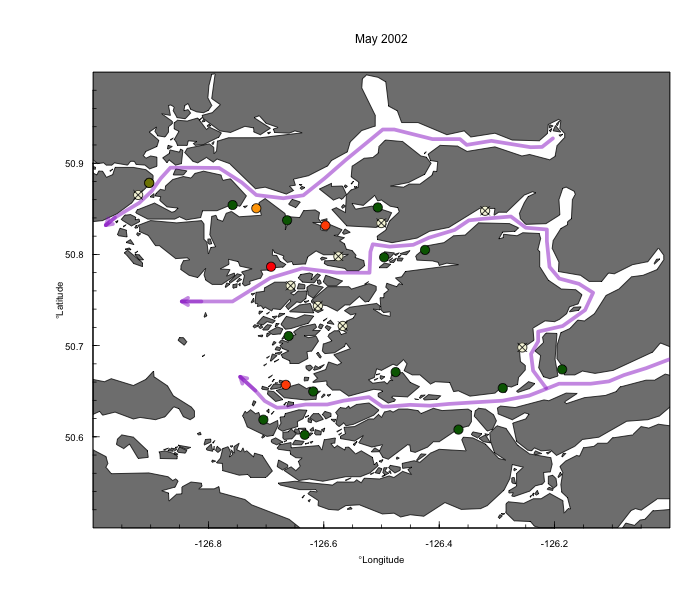

Supplement: Animation S1 — SLICE® treatments on Atlantic salmon farms in the Broughton Archipelago, British Columbia, Canada from November 1999 to December 2009 (Marty et al. 2010 PNAS). SLICE® treatment is indicated by a red circle at the salmon farm location. Untreated farms are shown by green circles, and farms fade from red to green over the four month efficacy period of SLICE® treatments (see main text). Fallowed farms (i.e., no Atlantic salmon in net pens) are indicated by beige circles with an ‘x’. Data that were used in the analysis of sea louse population dynamics before and after treatments (see main text) are circled in thick black. The period of juvenile wild salmon migration is indicated by purple arrows along approximate migration routes. (ZIP) [file pone.0060096.s001.zip › Animation S1/HTML_plot/BroughtonSLICE31.png]

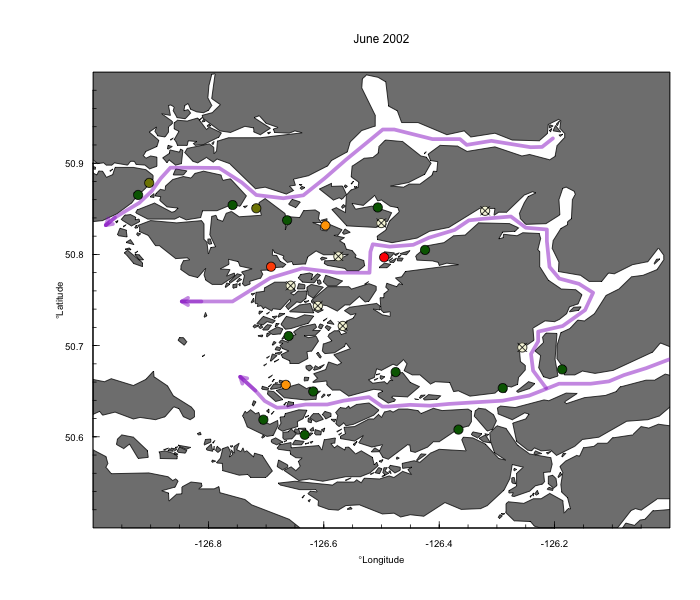

Supplement: Animation S1 — SLICE® treatments on Atlantic salmon farms in the Broughton Archipelago, British Columbia, Canada from November 1999 to December 2009 (Marty et al. 2010 PNAS). SLICE® treatment is indicated by a red circle at the salmon farm location. Untreated farms are shown by green circles, and farms fade from red to green over the four month efficacy period of SLICE® treatments (see main text). Fallowed farms (i.e., no Atlantic salmon in net pens) are indicated by beige circles with an ‘x’. Data that were used in the analysis of sea louse population dynamics before and after treatments (see main text) are circled in thick black. The period of juvenile wild salmon migration is indicated by purple arrows along approximate migration routes. (ZIP) [file pone.0060096.s001.zip › Animation S1/HTML_plot/BroughtonSLICE32.png]

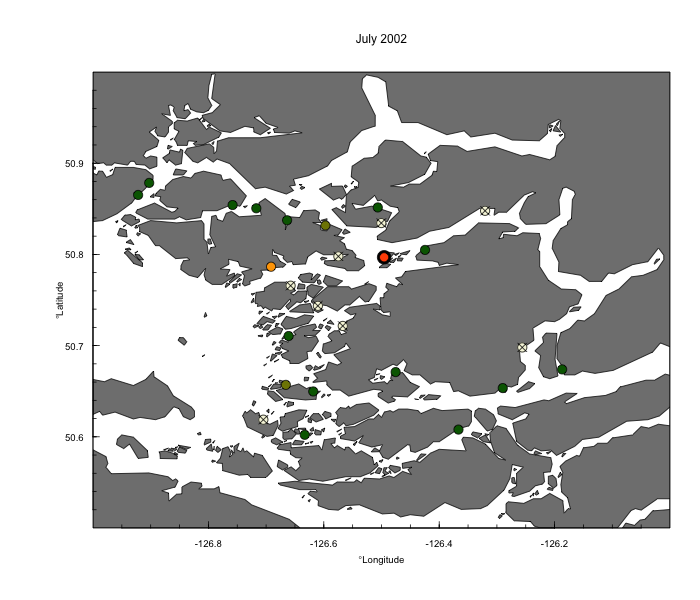

Supplement: Animation S1 — SLICE® treatments on Atlantic salmon farms in the Broughton Archipelago, British Columbia, Canada from November 1999 to December 2009 (Marty et al. 2010 PNAS). SLICE® treatment is indicated by a red circle at the salmon farm location. Untreated farms are shown by green circles, and farms fade from red to green over the four month efficacy period of SLICE® treatments (see main text). Fallowed farms (i.e., no Atlantic salmon in net pens) are indicated by beige circles with an ‘x’. Data that were used in the analysis of sea louse population dynamics before and after treatments (see main text) are circled in thick black. The period of juvenile wild salmon migration is indicated by purple arrows along approximate migration routes. (ZIP) [file pone.0060096.s001.zip › Animation S1/HTML_plot/BroughtonSLICE33.png]

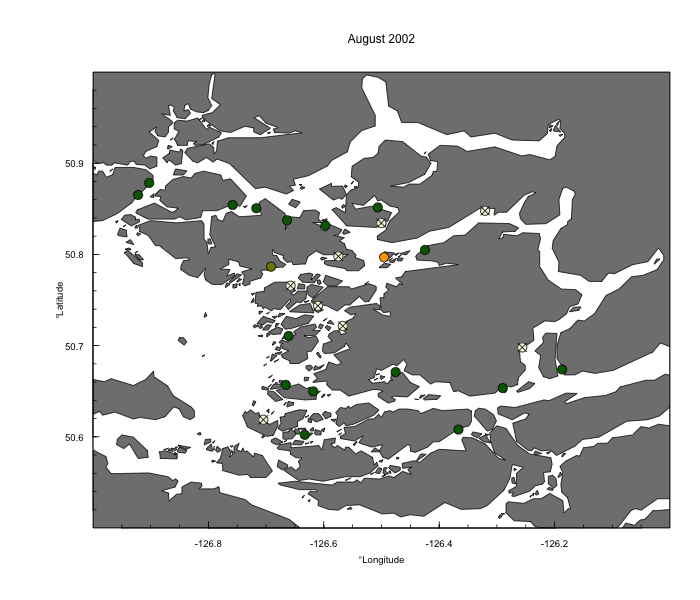

Supplement: Animation S1 — SLICE® treatments on Atlantic salmon farms in the Broughton Archipelago, British Columbia, Canada from November 1999 to December 2009 (Marty et al. 2010 PNAS). SLICE® treatment is indicated by a red circle at the salmon farm location. Untreated farms are shown by green circles, and farms fade from red to green over the four month efficacy period of SLICE® treatments (see main text). Fallowed farms (i.e., no Atlantic salmon in net pens) are indicated by beige circles with an ‘x’. Data that were used in the analysis of sea louse population dynamics before and after treatments (see main text) are circled in thick black. The period of juvenile wild salmon migration is indicated by purple arrows along approximate migration routes. (ZIP) [file pone.0060096.s001.zip › Animation S1/HTML_plot/BroughtonSLICE34.png]

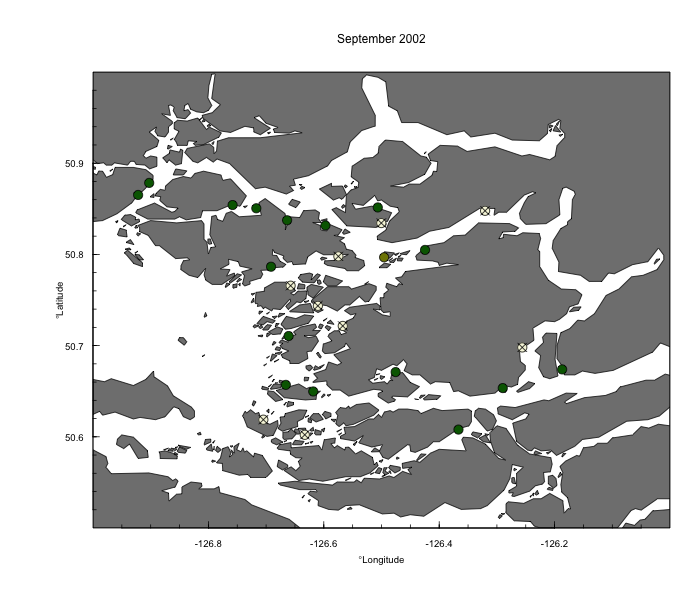

Supplement: Animation S1 — SLICE® treatments on Atlantic salmon farms in the Broughton Archipelago, British Columbia, Canada from November 1999 to December 2009 (Marty et al. 2010 PNAS). SLICE® treatment is indicated by a red circle at the salmon farm location. Untreated farms are shown by green circles, and farms fade from red to green over the four month efficacy period of SLICE® treatments (see main text). Fallowed farms (i.e., no Atlantic salmon in net pens) are indicated by beige circles with an ‘x’. Data that were used in the analysis of sea louse population dynamics before and after treatments (see main text) are circled in thick black. The period of juvenile wild salmon migration is indicated by purple arrows along approximate migration routes. (ZIP) [file pone.0060096.s001.zip › Animation S1/HTML_plot/BroughtonSLICE35.png]

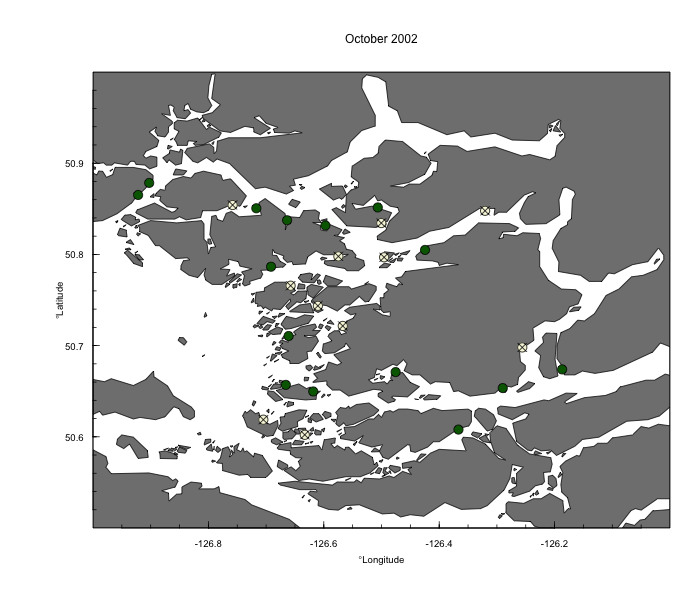

Supplement: Animation S1 — SLICE® treatments on Atlantic salmon farms in the Broughton Archipelago, British Columbia, Canada from November 1999 to December 2009 (Marty et al. 2010 PNAS). SLICE® treatment is indicated by a red circle at the salmon farm location. Untreated farms are shown by green circles, and farms fade from red to green over the four month efficacy period of SLICE® treatments (see main text). Fallowed farms (i.e., no Atlantic salmon in net pens) are indicated by beige circles with an ‘x’. Data that were used in the analysis of sea louse population dynamics before and after treatments (see main text) are circled in thick black. The period of juvenile wild salmon migration is indicated by purple arrows along approximate migration routes. (ZIP) [file pone.0060096.s001.zip › Animation S1/HTML_plot/BroughtonSLICE36.png]

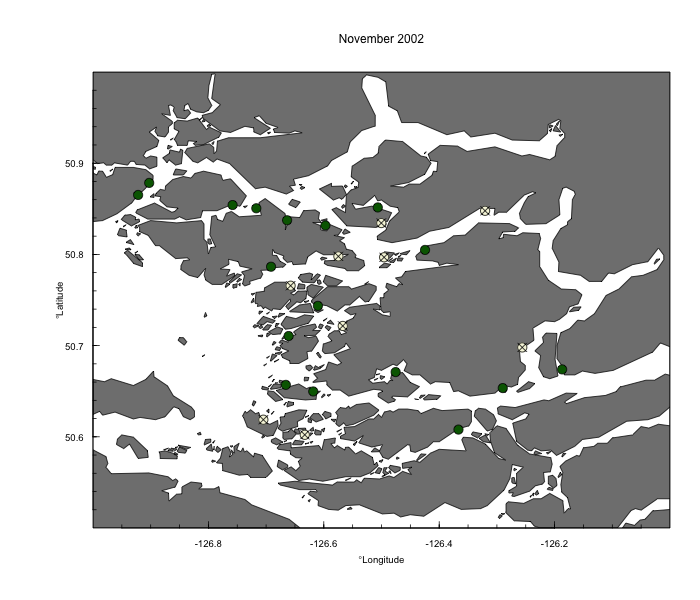

Supplement: Animation S1 — SLICE® treatments on Atlantic salmon farms in the Broughton Archipelago, British Columbia, Canada from November 1999 to December 2009 (Marty et al. 2010 PNAS). SLICE® treatment is indicated by a red circle at the salmon farm location. Untreated farms are shown by green circles, and farms fade from red to green over the four month efficacy period of SLICE® treatments (see main text). Fallowed farms (i.e., no Atlantic salmon in net pens) are indicated by beige circles with an ‘x’. Data that were used in the analysis of sea louse population dynamics before and after treatments (see main text) are circled in thick black. The period of juvenile wild salmon migration is indicated by purple arrows along approximate migration routes. (ZIP) [file pone.0060096.s001.zip › Animation S1/HTML_plot/BroughtonSLICE37.png]

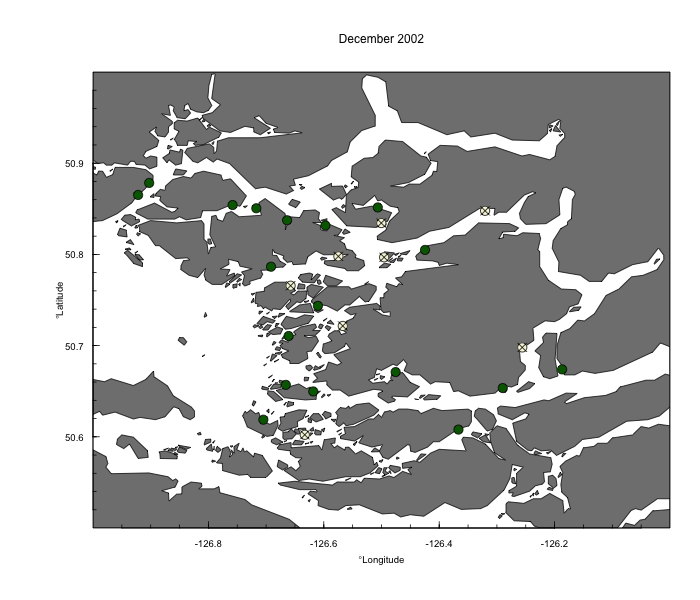

Supplement: Animation S1 — SLICE® treatments on Atlantic salmon farms in the Broughton Archipelago, British Columbia, Canada from November 1999 to December 2009 (Marty et al. 2010 PNAS). SLICE® treatment is indicated by a red circle at the salmon farm location. Untreated farms are shown by green circles, and farms fade from red to green over the four month efficacy period of SLICE® treatments (see main text). Fallowed farms (i.e., no Atlantic salmon in net pens) are indicated by beige circles with an ‘x’. Data that were used in the analysis of sea louse population dynamics before and after treatments (see main text) are circled in thick black. The period of juvenile wild salmon migration is indicated by purple arrows along approximate migration routes. (ZIP) [file pone.0060096.s001.zip › Animation S1/HTML_plot/BroughtonSLICE38.png]

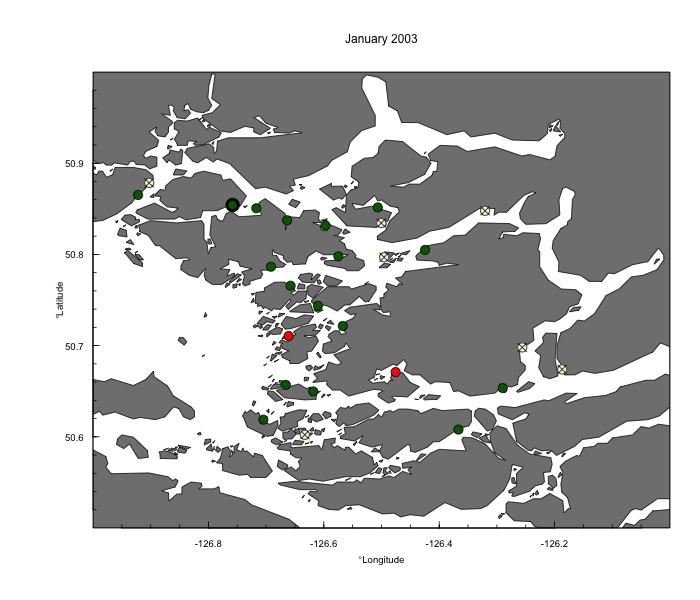

Supplement: Animation S1 — SLICE® treatments on Atlantic salmon farms in the Broughton Archipelago, British Columbia, Canada from November 1999 to December 2009 (Marty et al. 2010 PNAS). SLICE® treatment is indicated by a red circle at the salmon farm location. Untreated farms are shown by green circles, and farms fade from red to green over the four month efficacy period of SLICE® treatments (see main text). Fallowed farms (i.e., no Atlantic salmon in net pens) are indicated by beige circles with an ‘x’. Data that were used in the analysis of sea louse population dynamics before and after treatments (see main text) are circled in thick black. The period of juvenile wild salmon migration is indicated by purple arrows along approximate migration routes. (ZIP) [file pone.0060096.s001.zip › Animation S1/HTML_plot/BroughtonSLICE39.png]

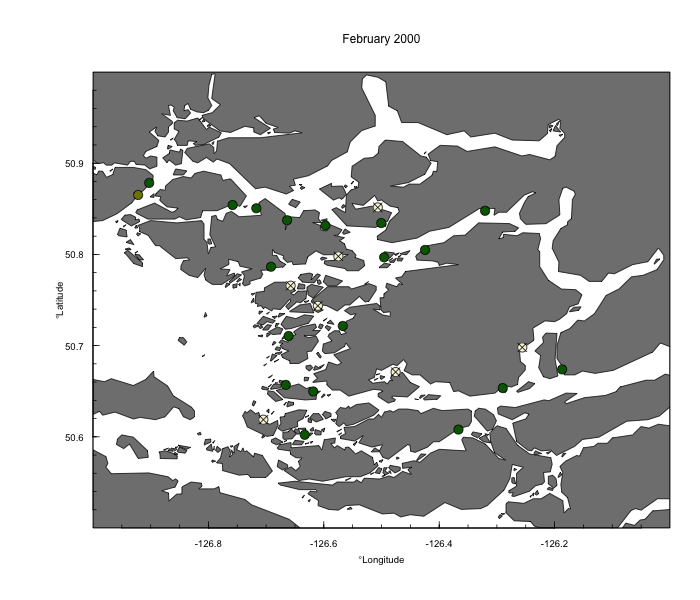

Supplement: Animation S1 — SLICE® treatments on Atlantic salmon farms in the Broughton Archipelago, British Columbia, Canada from November 1999 to December 2009 (Marty et al. 2010 PNAS). SLICE® treatment is indicated by a red circle at the salmon farm location. Untreated farms are shown by green circles, and farms fade from red to green over the four month efficacy period of SLICE® treatments (see main text). Fallowed farms (i.e., no Atlantic salmon in net pens) are indicated by beige circles with an ‘x’. Data that were used in the analysis of sea louse population dynamics before and after treatments (see main text) are circled in thick black. The period of juvenile wild salmon migration is indicated by purple arrows along approximate migration routes. (ZIP) [file pone.0060096.s001.zip › Animation S1/HTML_plot/BroughtonSLICE4.png]

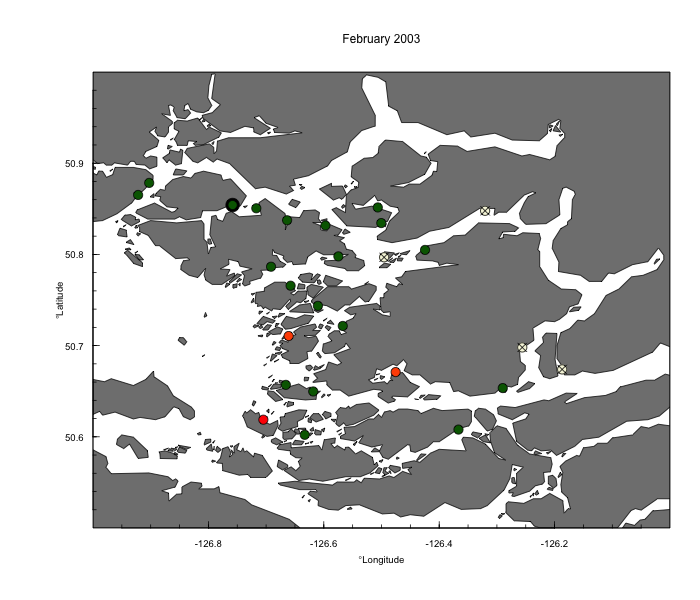

Supplement: Animation S1 — SLICE® treatments on Atlantic salmon farms in the Broughton Archipelago, British Columbia, Canada from November 1999 to December 2009 (Marty et al. 2010 PNAS). SLICE® treatment is indicated by a red circle at the salmon farm location. Untreated farms are shown by green circles, and farms fade from red to green over the four month efficacy period of SLICE® treatments (see main text). Fallowed farms (i.e., no Atlantic salmon in net pens) are indicated by beige circles with an ‘x’. Data that were used in the analysis of sea louse population dynamics before and after treatments (see main text) are circled in thick black. The period of juvenile wild salmon migration is indicated by purple arrows along approximate migration routes. (ZIP) [file pone.0060096.s001.zip › Animation S1/HTML_plot/BroughtonSLICE40.png]

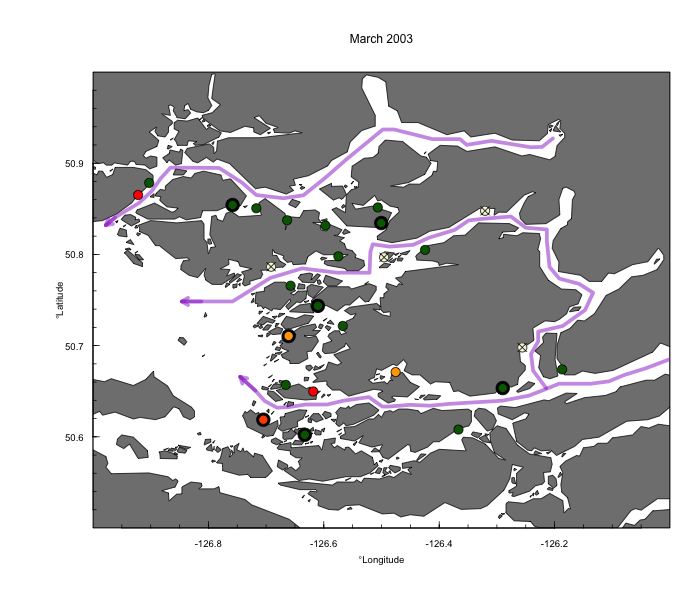

Supplement: Animation S1 — SLICE® treatments on Atlantic salmon farms in the Broughton Archipelago, British Columbia, Canada from November 1999 to December 2009 (Marty et al. 2010 PNAS). SLICE® treatment is indicated by a red circle at the salmon farm location. Untreated farms are shown by green circles, and farms fade from red to green over the four month efficacy period of SLICE® treatments (see main text). Fallowed farms (i.e., no Atlantic salmon in net pens) are indicated by beige circles with an ‘x’. Data that were used in the analysis of sea louse population dynamics before and after treatments (see main text) are circled in thick black. The period of juvenile wild salmon migration is indicated by purple arrows along approximate migration routes. (ZIP) [file pone.0060096.s001.zip › Animation S1/HTML_plot/BroughtonSLICE41.png]

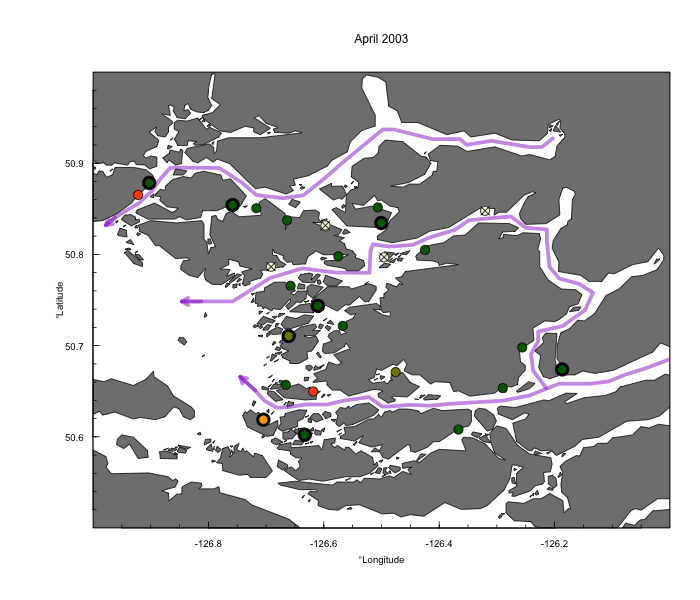

Supplement: Animation S1 — SLICE® treatments on Atlantic salmon farms in the Broughton Archipelago, British Columbia, Canada from November 1999 to December 2009 (Marty et al. 2010 PNAS). SLICE® treatment is indicated by a red circle at the salmon farm location. Untreated farms are shown by green circles, and farms fade from red to green over the four month efficacy period of SLICE® treatments (see main text). Fallowed farms (i.e., no Atlantic salmon in net pens) are indicated by beige circles with an ‘x’. Data that were used in the analysis of sea louse population dynamics before and after treatments (see main text) are circled in thick black. The period of juvenile wild salmon migration is indicated by purple arrows along approximate migration routes. (ZIP) [file pone.0060096.s001.zip › Animation S1/HTML_plot/BroughtonSLICE42.png]

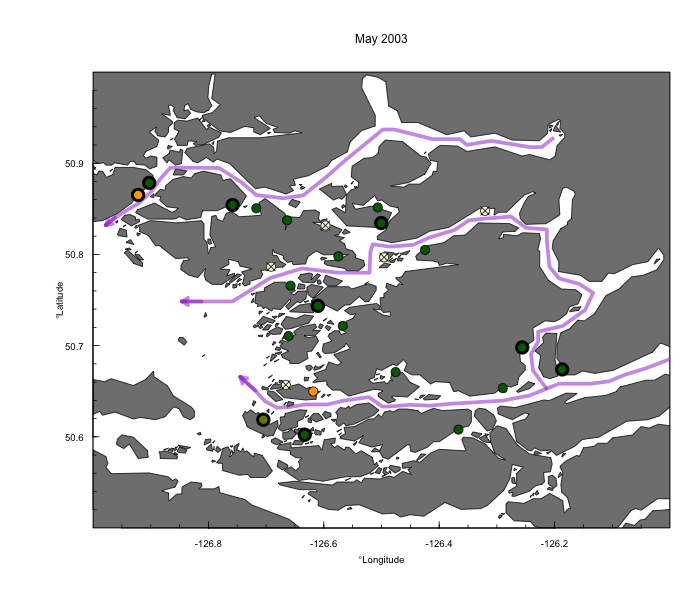

Supplement: Animation S1 — SLICE® treatments on Atlantic salmon farms in the Broughton Archipelago, British Columbia, Canada from November 1999 to December 2009 (Marty et al. 2010 PNAS). SLICE® treatment is indicated by a red circle at the salmon farm location. Untreated farms are shown by green circles, and farms fade from red to green over the four month efficacy period of SLICE® treatments (see main text). Fallowed farms (i.e., no Atlantic salmon in net pens) are indicated by beige circles with an ‘x’. Data that were used in the analysis of sea louse population dynamics before and after treatments (see main text) are circled in thick black. The period of juvenile wild salmon migration is indicated by purple arrows along approximate migration routes. (ZIP) [file pone.0060096.s001.zip › Animation S1/HTML_plot/BroughtonSLICE43.png]

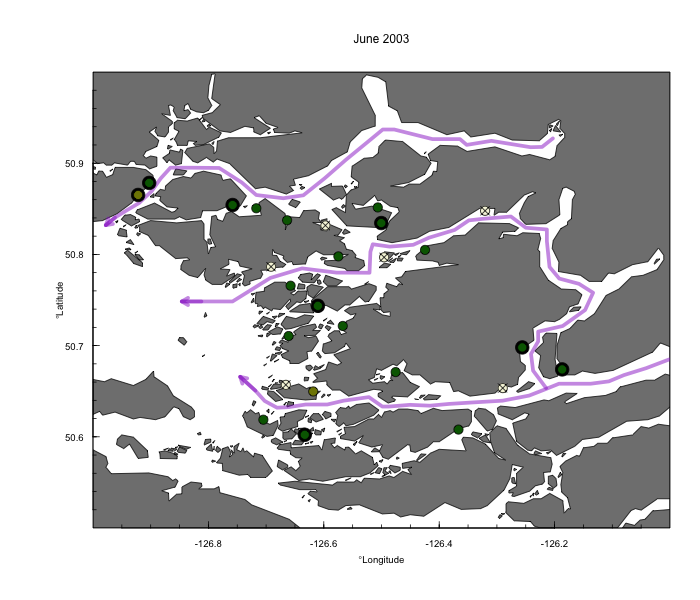

Supplement: Animation S1 — SLICE® treatments on Atlantic salmon farms in the Broughton Archipelago, British Columbia, Canada from November 1999 to December 2009 (Marty et al. 2010 PNAS). SLICE® treatment is indicated by a red circle at the salmon farm location. Untreated farms are shown by green circles, and farms fade from red to green over the four month efficacy period of SLICE® treatments (see main text). Fallowed farms (i.e., no Atlantic salmon in net pens) are indicated by beige circles with an ‘x’. Data that were used in the analysis of sea louse population dynamics before and after treatments (see main text) are circled in thick black. The period of juvenile wild salmon migration is indicated by purple arrows along approximate migration routes. (ZIP) [file pone.0060096.s001.zip › Animation S1/HTML_plot/BroughtonSLICE44.png]

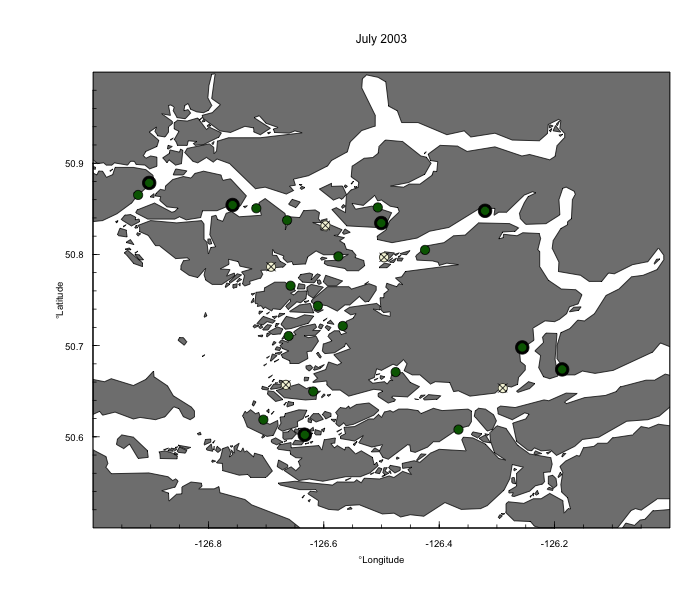

Supplement: Animation S1 — SLICE® treatments on Atlantic salmon farms in the Broughton Archipelago, British Columbia, Canada from November 1999 to December 2009 (Marty et al. 2010 PNAS). SLICE® treatment is indicated by a red circle at the salmon farm location. Untreated farms are shown by green circles, and farms fade from red to green over the four month efficacy period of SLICE® treatments (see main text). Fallowed farms (i.e., no Atlantic salmon in net pens) are indicated by beige circles with an ‘x’. Data that were used in the analysis of sea louse population dynamics before and after treatments (see main text) are circled in thick black. The period of juvenile wild salmon migration is indicated by purple arrows along approximate migration routes. (ZIP) [file pone.0060096.s001.zip › Animation S1/HTML_plot/BroughtonSLICE45.png]

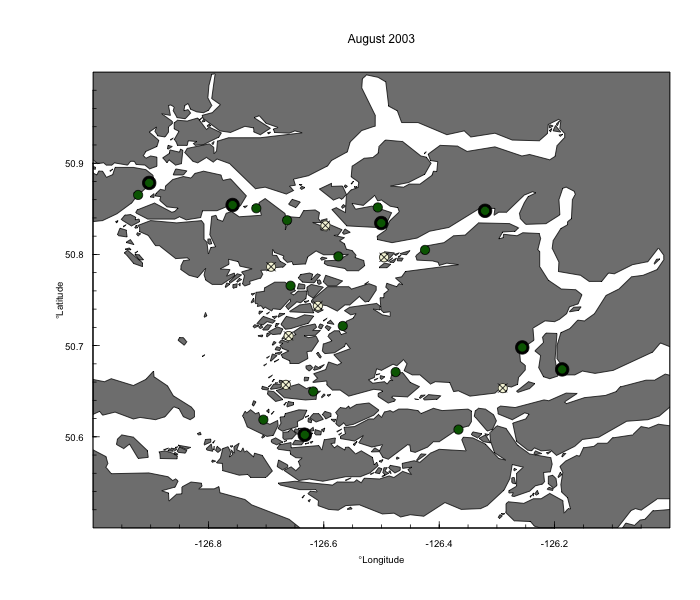

Supplement: Animation S1 — SLICE® treatments on Atlantic salmon farms in the Broughton Archipelago, British Columbia, Canada from November 1999 to December 2009 (Marty et al. 2010 PNAS). SLICE® treatment is indicated by a red circle at the salmon farm location. Untreated farms are shown by green circles, and farms fade from red to green over the four month efficacy period of SLICE® treatments (see main text). Fallowed farms (i.e., no Atlantic salmon in net pens) are indicated by beige circles with an ‘x’. Data that were used in the analysis of sea louse population dynamics before and after treatments (see main text) are circled in thick black. The period of juvenile wild salmon migration is indicated by purple arrows along approximate migration routes. (ZIP) [file pone.0060096.s001.zip › Animation S1/HTML_plot/BroughtonSLICE46.png]

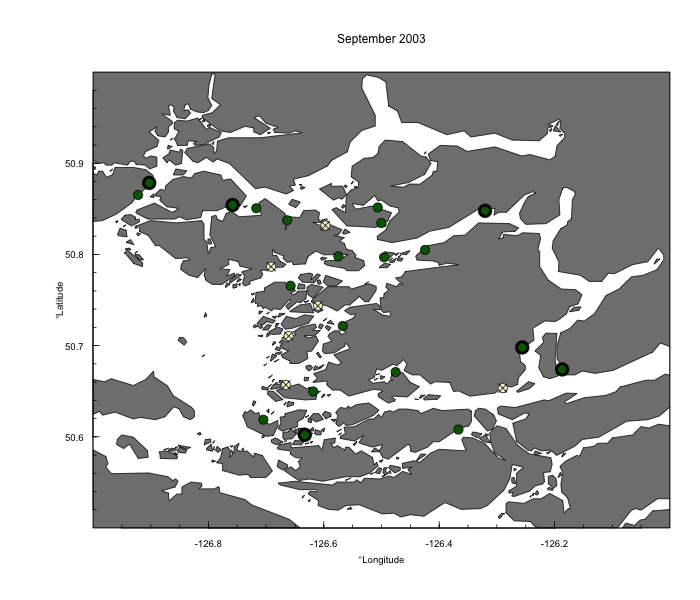

Supplement: Animation S1 — SLICE® treatments on Atlantic salmon farms in the Broughton Archipelago, British Columbia, Canada from November 1999 to December 2009 (Marty et al. 2010 PNAS). SLICE® treatment is indicated by a red circle at the salmon farm location. Untreated farms are shown by green circles, and farms fade from red to green over the four month efficacy period of SLICE® treatments (see main text). Fallowed farms (i.e., no Atlantic salmon in net pens) are indicated by beige circles with an ‘x’. Data that were used in the analysis of sea louse population dynamics before and after treatments (see main text) are circled in thick black. The period of juvenile wild salmon migration is indicated by purple arrows along approximate migration routes. (ZIP) [file pone.0060096.s001.zip › Animation S1/HTML_plot/BroughtonSLICE47.png]

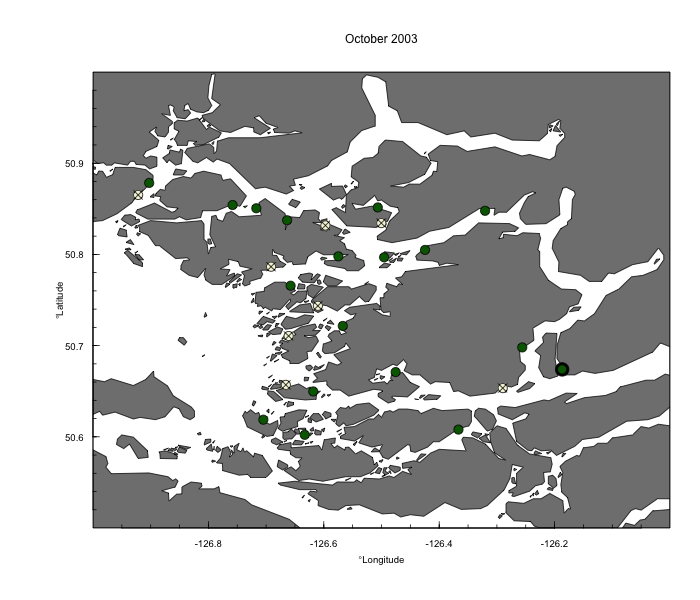

Supplement: Animation S1 — SLICE® treatments on Atlantic salmon farms in the Broughton Archipelago, British Columbia, Canada from November 1999 to December 2009 (Marty et al. 2010 PNAS). SLICE® treatment is indicated by a red circle at the salmon farm location. Untreated farms are shown by green circles, and farms fade from red to green over the four month efficacy period of SLICE® treatments (see main text). Fallowed farms (i.e., no Atlantic salmon in net pens) are indicated by beige circles with an ‘x’. Data that were used in the analysis of sea louse population dynamics before and after treatments (see main text) are circled in thick black. The period of juvenile wild salmon migration is indicated by purple arrows along approximate migration routes. (ZIP) [file pone.0060096.s001.zip › Animation S1/HTML_plot/BroughtonSLICE48.png]

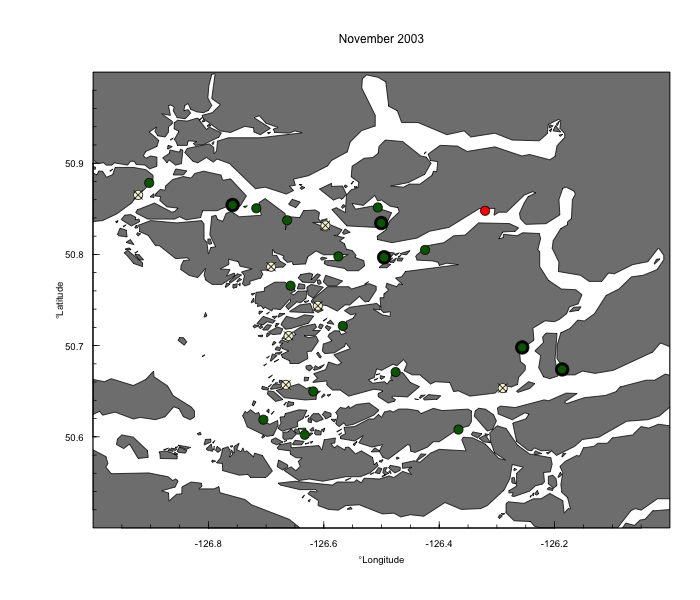

Supplement: Animation S1 — SLICE® treatments on Atlantic salmon farms in the Broughton Archipelago, British Columbia, Canada from November 1999 to December 2009 (Marty et al. 2010 PNAS). SLICE® treatment is indicated by a red circle at the salmon farm location. Untreated farms are shown by green circles, and farms fade from red to green over the four month efficacy period of SLICE® treatments (see main text). Fallowed farms (i.e., no Atlantic salmon in net pens) are indicated by beige circles with an ‘x’. Data that were used in the analysis of sea louse population dynamics before and after treatments (see main text) are circled in thick black. The period of juvenile wild salmon migration is indicated by purple arrows along approximate migration routes. (ZIP) [file pone.0060096.s001.zip › Animation S1/HTML_plot/BroughtonSLICE49.png]

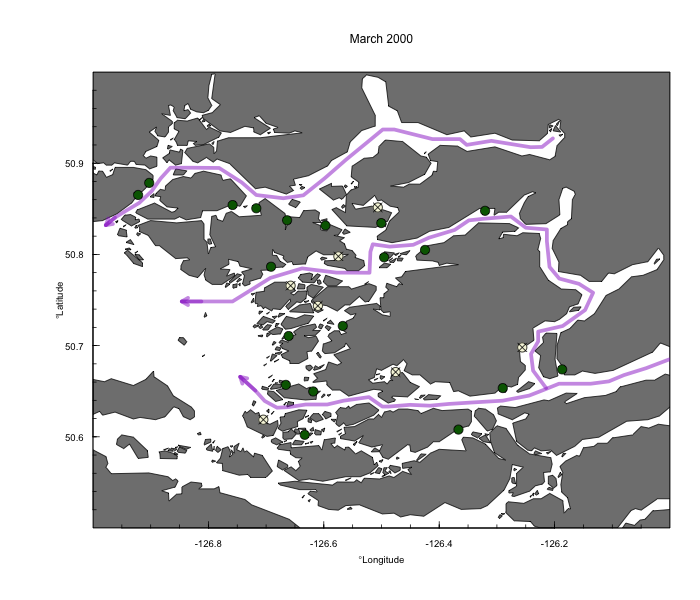

Supplement: Animation S1 — SLICE® treatments on Atlantic salmon farms in the Broughton Archipelago, British Columbia, Canada from November 1999 to December 2009 (Marty et al. 2010 PNAS). SLICE® treatment is indicated by a red circle at the salmon farm location. Untreated farms are shown by green circles, and farms fade from red to green over the four month efficacy period of SLICE® treatments (see main text). Fallowed farms (i.e., no Atlantic salmon in net pens) are indicated by beige circles with an ‘x’. Data that were used in the analysis of sea louse population dynamics before and after treatments (see main text) are circled in thick black. The period of juvenile wild salmon migration is indicated by purple arrows along approximate migration routes. (ZIP) [file pone.0060096.s001.zip › Animation S1/HTML_plot/BroughtonSLICE5.png]

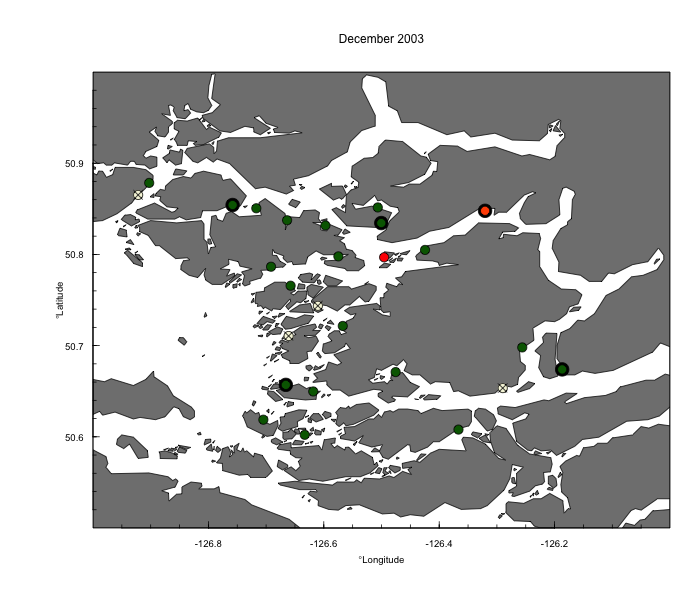

Supplement: Animation S1 — SLICE® treatments on Atlantic salmon farms in the Broughton Archipelago, British Columbia, Canada from November 1999 to December 2009 (Marty et al. 2010 PNAS). SLICE® treatment is indicated by a red circle at the salmon farm location. Untreated farms are shown by green circles, and farms fade from red to green over the four month efficacy period of SLICE® treatments (see main text). Fallowed farms (i.e., no Atlantic salmon in net pens) are indicated by beige circles with an ‘x’. Data that were used in the analysis of sea louse population dynamics before and after treatments (see main text) are circled in thick black. The period of juvenile wild salmon migration is indicated by purple arrows along approximate migration routes. (ZIP) [file pone.0060096.s001.zip › Animation S1/HTML_plot/BroughtonSLICE50.png]

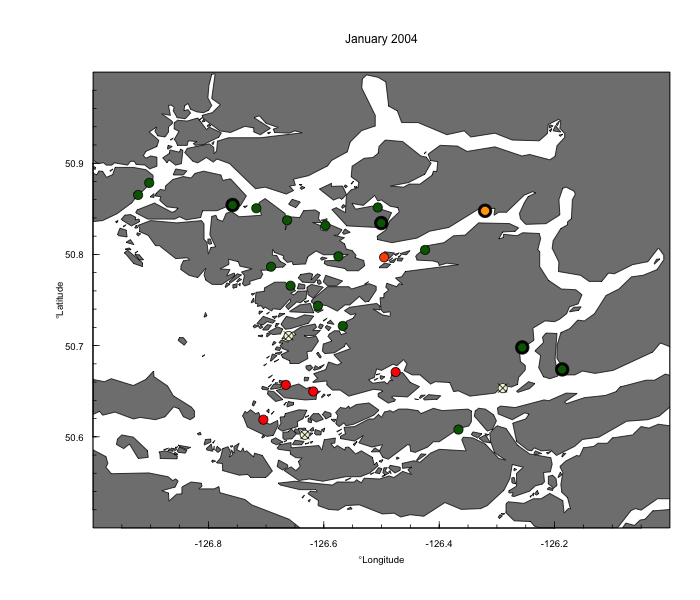

Supplement: Animation S1 — SLICE® treatments on Atlantic salmon farms in the Broughton Archipelago, British Columbia, Canada from November 1999 to December 2009 (Marty et al. 2010 PNAS). SLICE® treatment is indicated by a red circle at the salmon farm location. Untreated farms are shown by green circles, and farms fade from red to green over the four month efficacy period of SLICE® treatments (see main text). Fallowed farms (i.e., no Atlantic salmon in net pens) are indicated by beige circles with an ‘x’. Data that were used in the analysis of sea louse population dynamics before and after treatments (see main text) are circled in thick black. The period of juvenile wild salmon migration is indicated by purple arrows along approximate migration routes. (ZIP) [file pone.0060096.s001.zip › Animation S1/HTML_plot/BroughtonSLICE51.png]

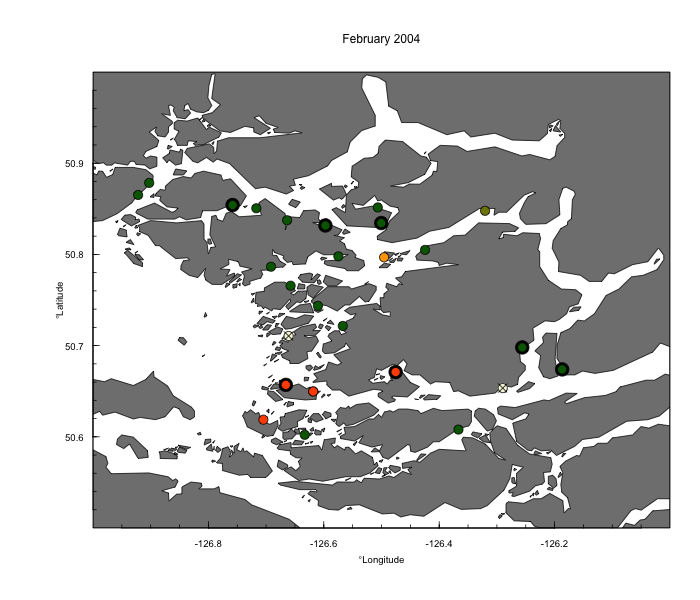

Supplement: Animation S1 — SLICE® treatments on Atlantic salmon farms in the Broughton Archipelago, British Columbia, Canada from November 1999 to December 2009 (Marty et al. 2010 PNAS). SLICE® treatment is indicated by a red circle at the salmon farm location. Untreated farms are shown by green circles, and farms fade from red to green over the four month efficacy period of SLICE® treatments (see main text). Fallowed farms (i.e., no Atlantic salmon in net pens) are indicated by beige circles with an ‘x’. Data that were used in the analysis of sea louse population dynamics before and after treatments (see main text) are circled in thick black. The period of juvenile wild salmon migration is indicated by purple arrows along approximate migration routes. (ZIP) [file pone.0060096.s001.zip › Animation S1/HTML_plot/BroughtonSLICE52.png]

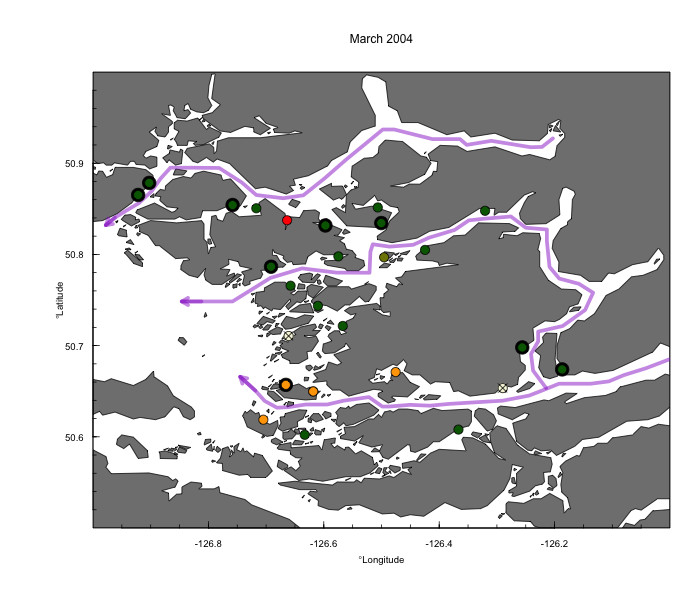

Supplement: Animation S1 — SLICE® treatments on Atlantic salmon farms in the Broughton Archipelago, British Columbia, Canada from November 1999 to December 2009 (Marty et al. 2010 PNAS). SLICE® treatment is indicated by a red circle at the salmon farm location. Untreated farms are shown by green circles, and farms fade from red to green over the four month efficacy period of SLICE® treatments (see main text). Fallowed farms (i.e., no Atlantic salmon in net pens) are indicated by beige circles with an ‘x’. Data that were used in the analysis of sea louse population dynamics before and after treatments (see main text) are circled in thick black. The period of juvenile wild salmon migration is indicated by purple arrows along approximate migration routes. (ZIP) [file pone.0060096.s001.zip › Animation S1/HTML_plot/BroughtonSLICE53.png]

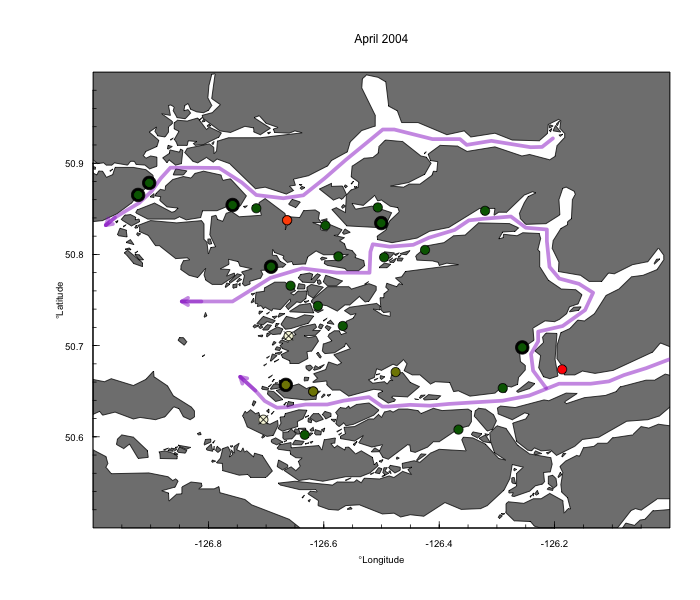

Supplement: Animation S1 — SLICE® treatments on Atlantic salmon farms in the Broughton Archipelago, British Columbia, Canada from November 1999 to December 2009 (Marty et al. 2010 PNAS). SLICE® treatment is indicated by a red circle at the salmon farm location. Untreated farms are shown by green circles, and farms fade from red to green over the four month efficacy period of SLICE® treatments (see main text). Fallowed farms (i.e., no Atlantic salmon in net pens) are indicated by beige circles with an ‘x’. Data that were used in the analysis of sea louse population dynamics before and after treatments (see main text) are circled in thick black. The period of juvenile wild salmon migration is indicated by purple arrows along approximate migration routes. (ZIP) [file pone.0060096.s001.zip › Animation S1/HTML_plot/BroughtonSLICE54.png]

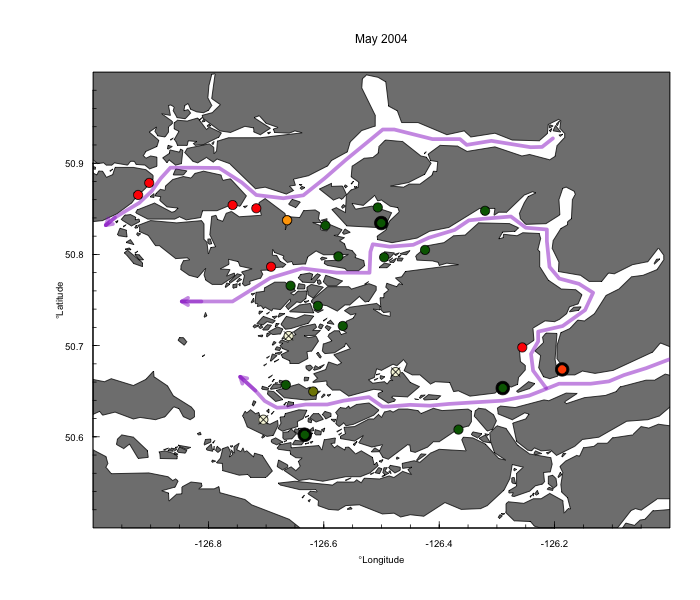

Supplement: Animation S1 — SLICE® treatments on Atlantic salmon farms in the Broughton Archipelago, British Columbia, Canada from November 1999 to December 2009 (Marty et al. 2010 PNAS). SLICE® treatment is indicated by a red circle at the salmon farm location. Untreated farms are shown by green circles, and farms fade from red to green over the four month efficacy period of SLICE® treatments (see main text). Fallowed farms (i.e., no Atlantic salmon in net pens) are indicated by beige circles with an ‘x’. Data that were used in the analysis of sea louse population dynamics before and after treatments (see main text) are circled in thick black. The period of juvenile wild salmon migration is indicated by purple arrows along approximate migration routes. (ZIP) [file pone.0060096.s001.zip › Animation S1/HTML_plot/BroughtonSLICE55.png]

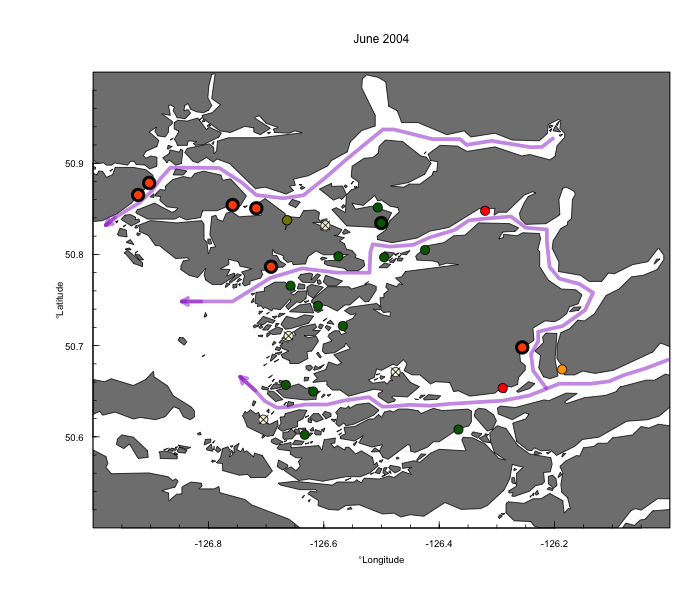

Supplement: Animation S1 — SLICE® treatments on Atlantic salmon farms in the Broughton Archipelago, British Columbia, Canada from November 1999 to December 2009 (Marty et al. 2010 PNAS). SLICE® treatment is indicated by a red circle at the salmon farm location. Untreated farms are shown by green circles, and farms fade from red to green over the four month efficacy period of SLICE® treatments (see main text). Fallowed farms (i.e., no Atlantic salmon in net pens) are indicated by beige circles with an ‘x’. Data that were used in the analysis of sea louse population dynamics before and after treatments (see main text) are circled in thick black. The period of juvenile wild salmon migration is indicated by purple arrows along approximate migration routes. (ZIP) [file pone.0060096.s001.zip › Animation S1/HTML_plot/BroughtonSLICE56.png]

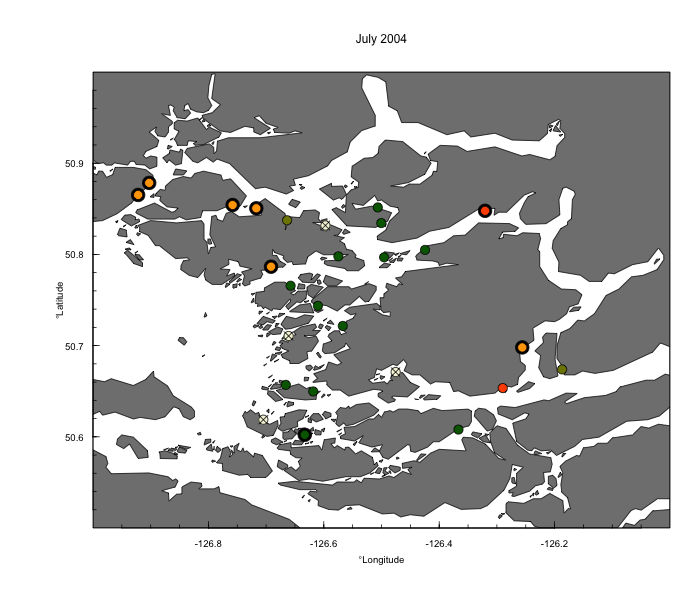

Supplement: Animation S1 — SLICE® treatments on Atlantic salmon farms in the Broughton Archipelago, British Columbia, Canada from November 1999 to December 2009 (Marty et al. 2010 PNAS). SLICE® treatment is indicated by a red circle at the salmon farm location. Untreated farms are shown by green circles, and farms fade from red to green over the four month efficacy period of SLICE® treatments (see main text). Fallowed farms (i.e., no Atlantic salmon in net pens) are indicated by beige circles with an ‘x’. Data that were used in the analysis of sea louse population dynamics before and after treatments (see main text) are circled in thick black. The period of juvenile wild salmon migration is indicated by purple arrows along approximate migration routes. (ZIP) [file pone.0060096.s001.zip › Animation S1/HTML_plot/BroughtonSLICE57.png]

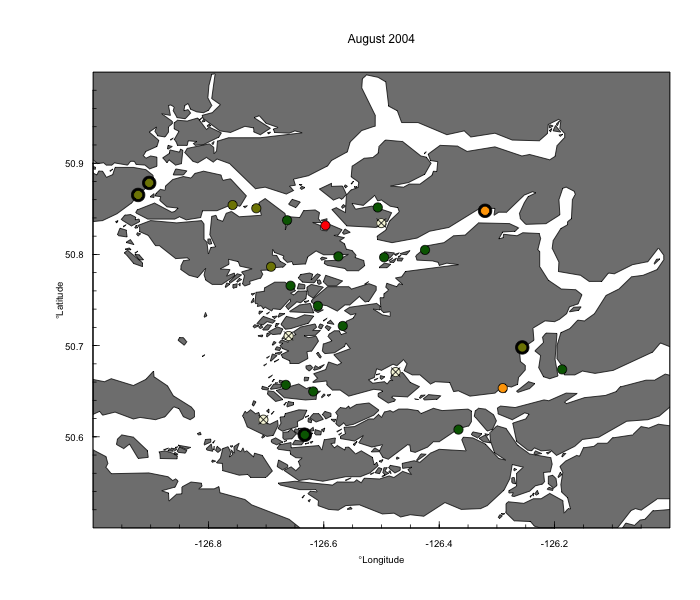

Supplement: Animation S1 — SLICE® treatments on Atlantic salmon farms in the Broughton Archipelago, British Columbia, Canada from November 1999 to December 2009 (Marty et al. 2010 PNAS). SLICE® treatment is indicated by a red circle at the salmon farm location. Untreated farms are shown by green circles, and farms fade from red to green over the four month efficacy period of SLICE® treatments (see main text). Fallowed farms (i.e., no Atlantic salmon in net pens) are indicated by beige circles with an ‘x’. Data that were used in the analysis of sea louse population dynamics before and after treatments (see main text) are circled in thick black. The period of juvenile wild salmon migration is indicated by purple arrows along approximate migration routes. (ZIP) [file pone.0060096.s001.zip › Animation S1/HTML_plot/BroughtonSLICE58.png]

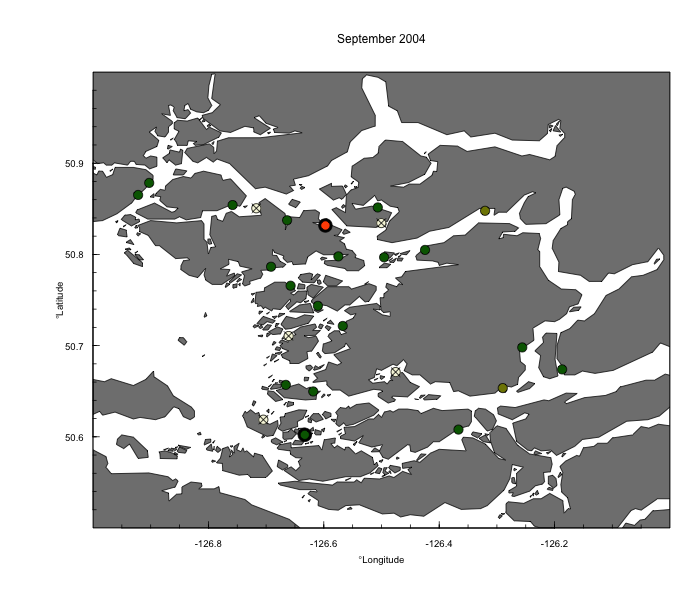

Supplement: Animation S1 — SLICE® treatments on Atlantic salmon farms in the Broughton Archipelago, British Columbia, Canada from November 1999 to December 2009 (Marty et al. 2010 PNAS). SLICE® treatment is indicated by a red circle at the salmon farm location. Untreated farms are shown by green circles, and farms fade from red to green over the four month efficacy period of SLICE® treatments (see main text). Fallowed farms (i.e., no Atlantic salmon in net pens) are indicated by beige circles with an ‘x’. Data that were used in the analysis of sea louse population dynamics before and after treatments (see main text) are circled in thick black. The period of juvenile wild salmon migration is indicated by purple arrows along approximate migration routes. (ZIP) [file pone.0060096.s001.zip › Animation S1/HTML_plot/BroughtonSLICE59.png]

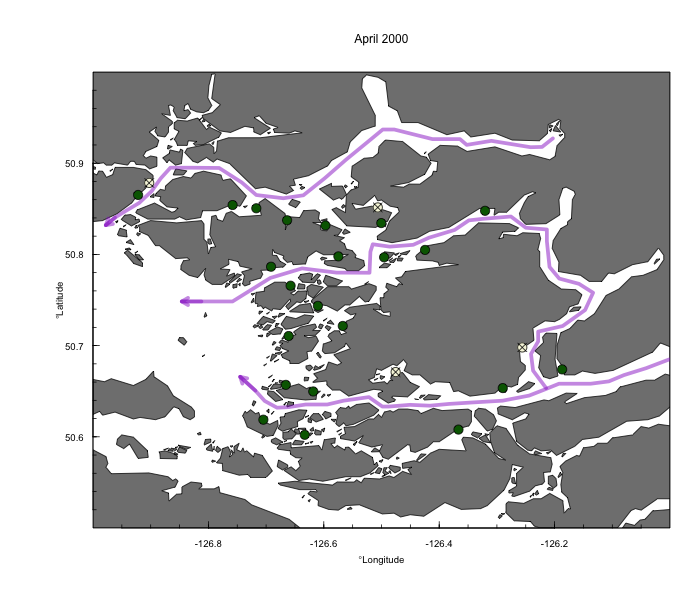

Supplement: Animation S1 — SLICE® treatments on Atlantic salmon farms in the Broughton Archipelago, British Columbia, Canada from November 1999 to December 2009 (Marty et al. 2010 PNAS). SLICE® treatment is indicated by a red circle at the salmon farm location. Untreated farms are shown by green circles, and farms fade from red to green over the four month efficacy period of SLICE® treatments (see main text). Fallowed farms (i.e., no Atlantic salmon in net pens) are indicated by beige circles with an ‘x’. Data that were used in the analysis of sea louse population dynamics before and after treatments (see main text) are circled in thick black. The period of juvenile wild salmon migration is indicated by purple arrows along approximate migration routes. (ZIP) [file pone.0060096.s001.zip › Animation S1/HTML_plot/BroughtonSLICE6.png]

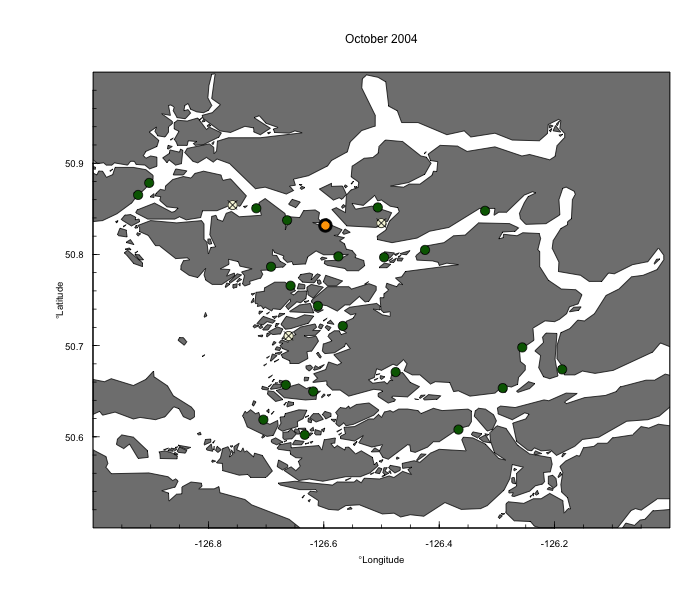

Supplement: Animation S1 — SLICE® treatments on Atlantic salmon farms in the Broughton Archipelago, British Columbia, Canada from November 1999 to December 2009 (Marty et al. 2010 PNAS). SLICE® treatment is indicated by a red circle at the salmon farm location. Untreated farms are shown by green circles, and farms fade from red to green over the four month efficacy period of SLICE® treatments (see main text). Fallowed farms (i.e., no Atlantic salmon in net pens) are indicated by beige circles with an ‘x’. Data that were used in the analysis of sea louse population dynamics before and after treatments (see main text) are circled in thick black. The period of juvenile wild salmon migration is indicated by purple arrows along approximate migration routes. (ZIP) [file pone.0060096.s001.zip › Animation S1/HTML_plot/BroughtonSLICE60.png]

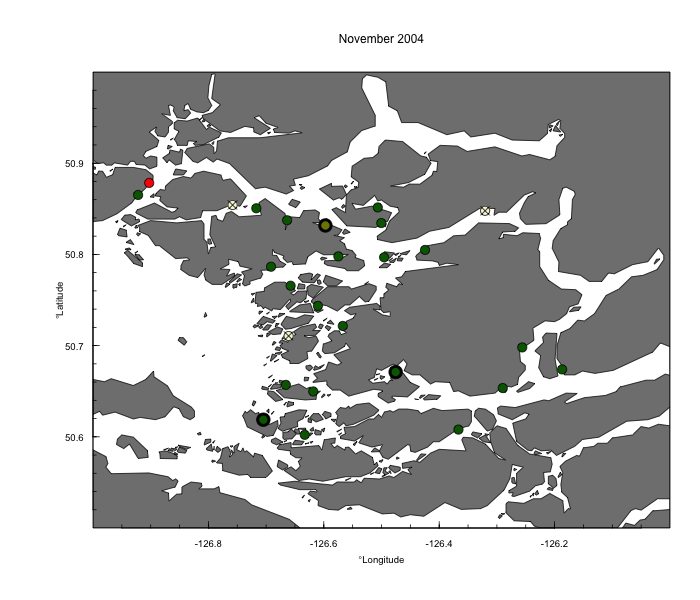

Supplement: Animation S1 — SLICE® treatments on Atlantic salmon farms in the Broughton Archipelago, British Columbia, Canada from November 1999 to December 2009 (Marty et al. 2010 PNAS). SLICE® treatment is indicated by a red circle at the salmon farm location. Untreated farms are shown by green circles, and farms fade from red to green over the four month efficacy period of SLICE® treatments (see main text). Fallowed farms (i.e., no Atlantic salmon in net pens) are indicated by beige circles with an ‘x’. Data that were used in the analysis of sea louse population dynamics before and after treatments (see main text) are circled in thick black. The period of juvenile wild salmon migration is indicated by purple arrows along approximate migration routes. (ZIP) [file pone.0060096.s001.zip › Animation S1/HTML_plot/BroughtonSLICE61.png]

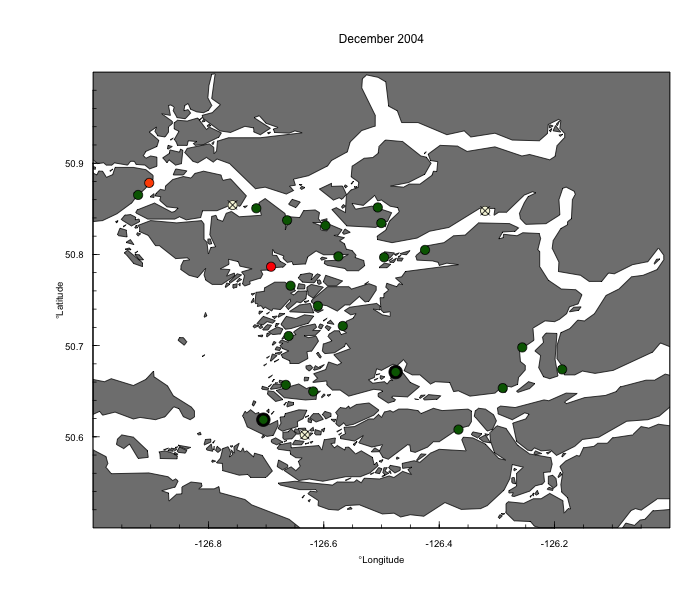

Supplement: Animation S1 — SLICE® treatments on Atlantic salmon farms in the Broughton Archipelago, British Columbia, Canada from November 1999 to December 2009 (Marty et al. 2010 PNAS). SLICE® treatment is indicated by a red circle at the salmon farm location. Untreated farms are shown by green circles, and farms fade from red to green over the four month efficacy period of SLICE® treatments (see main text). Fallowed farms (i.e., no Atlantic salmon in net pens) are indicated by beige circles with an ‘x’. Data that were used in the analysis of sea louse population dynamics before and after treatments (see main text) are circled in thick black. The period of juvenile wild salmon migration is indicated by purple arrows along approximate migration routes. (ZIP) [file pone.0060096.s001.zip › Animation S1/HTML_plot/BroughtonSLICE62.png]

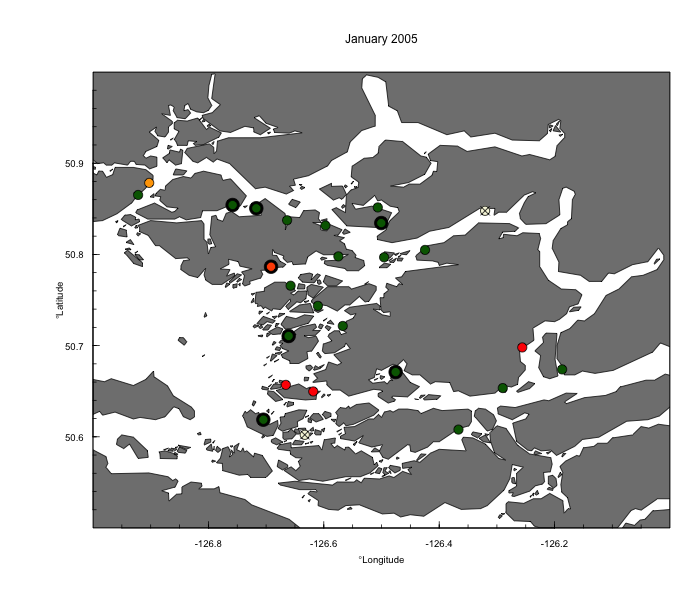

Supplement: Animation S1 — SLICE® treatments on Atlantic salmon farms in the Broughton Archipelago, British Columbia, Canada from November 1999 to December 2009 (Marty et al. 2010 PNAS). SLICE® treatment is indicated by a red circle at the salmon farm location. Untreated farms are shown by green circles, and farms fade from red to green over the four month efficacy period of SLICE® treatments (see main text). Fallowed farms (i.e., no Atlantic salmon in net pens) are indicated by beige circles with an ‘x’. Data that were used in the analysis of sea louse population dynamics before and after treatments (see main text) are circled in thick black. The period of juvenile wild salmon migration is indicated by purple arrows along approximate migration routes. (ZIP) [file pone.0060096.s001.zip › Animation S1/HTML_plot/BroughtonSLICE63.png]

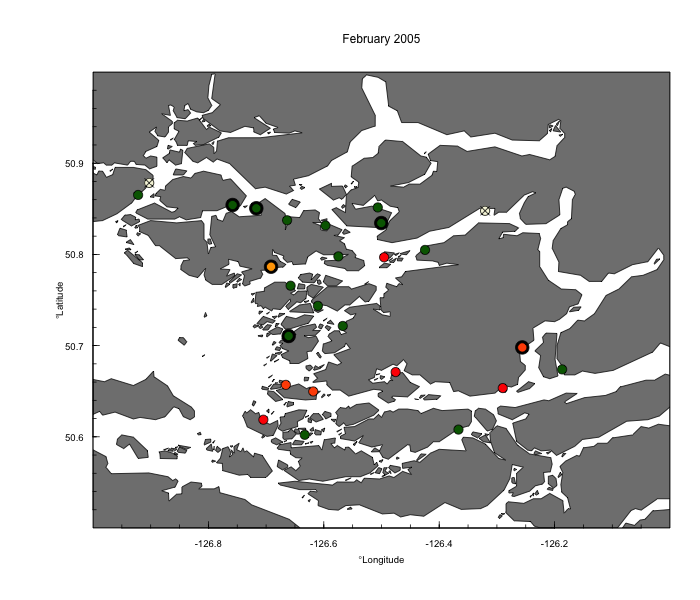

Supplement: Animation S1 — SLICE® treatments on Atlantic salmon farms in the Broughton Archipelago, British Columbia, Canada from November 1999 to December 2009 (Marty et al. 2010 PNAS). SLICE® treatment is indicated by a red circle at the salmon farm location. Untreated farms are shown by green circles, and farms fade from red to green over the four month efficacy period of SLICE® treatments (see main text). Fallowed farms (i.e., no Atlantic salmon in net pens) are indicated by beige circles with an ‘x’. Data that were used in the analysis of sea louse population dynamics before and after treatments (see main text) are circled in thick black. The period of juvenile wild salmon migration is indicated by purple arrows along approximate migration routes. (ZIP) [file pone.0060096.s001.zip › Animation S1/HTML_plot/BroughtonSLICE64.png]

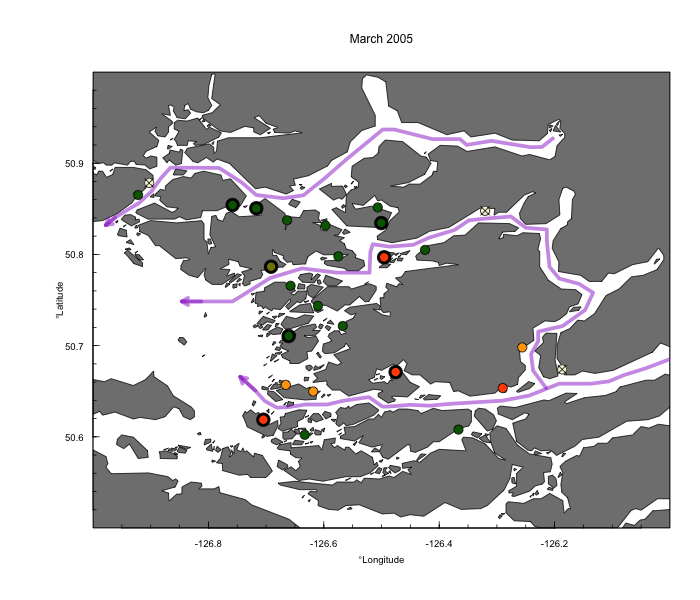

Supplement: Animation S1 — SLICE® treatments on Atlantic salmon farms in the Broughton Archipelago, British Columbia, Canada from November 1999 to December 2009 (Marty et al. 2010 PNAS). SLICE® treatment is indicated by a red circle at the salmon farm location. Untreated farms are shown by green circles, and farms fade from red to green over the four month efficacy period of SLICE® treatments (see main text). Fallowed farms (i.e., no Atlantic salmon in net pens) are indicated by beige circles with an ‘x’. Data that were used in the analysis of sea louse population dynamics before and after treatments (see main text) are circled in thick black. The period of juvenile wild salmon migration is indicated by purple arrows along approximate migration routes. (ZIP) [file pone.0060096.s001.zip › Animation S1/HTML_plot/BroughtonSLICE65.png]

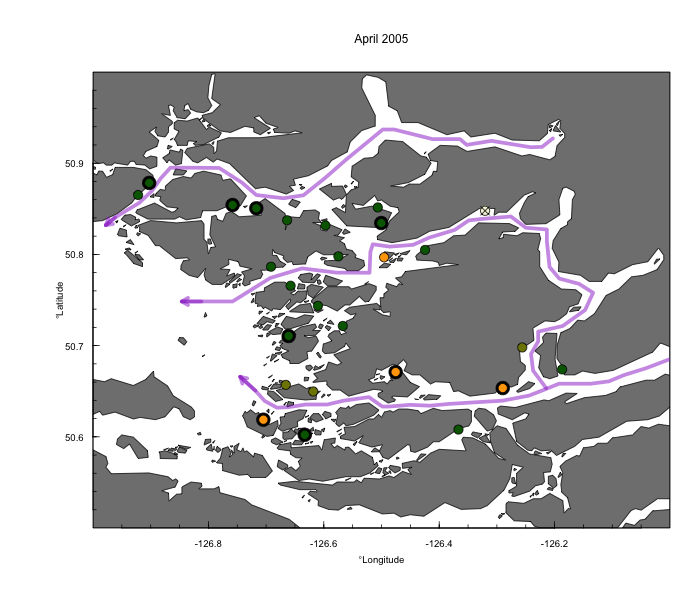

Supplement: Animation S1 — SLICE® treatments on Atlantic salmon farms in the Broughton Archipelago, British Columbia, Canada from November 1999 to December 2009 (Marty et al. 2010 PNAS). SLICE® treatment is indicated by a red circle at the salmon farm location. Untreated farms are shown by green circles, and farms fade from red to green over the four month efficacy period of SLICE® treatments (see main text). Fallowed farms (i.e., no Atlantic salmon in net pens) are indicated by beige circles with an ‘x’. Data that were used in the analysis of sea louse population dynamics before and after treatments (see main text) are circled in thick black. The period of juvenile wild salmon migration is indicated by purple arrows along approximate migration routes. (ZIP) [file pone.0060096.s001.zip › Animation S1/HTML_plot/BroughtonSLICE66.png]

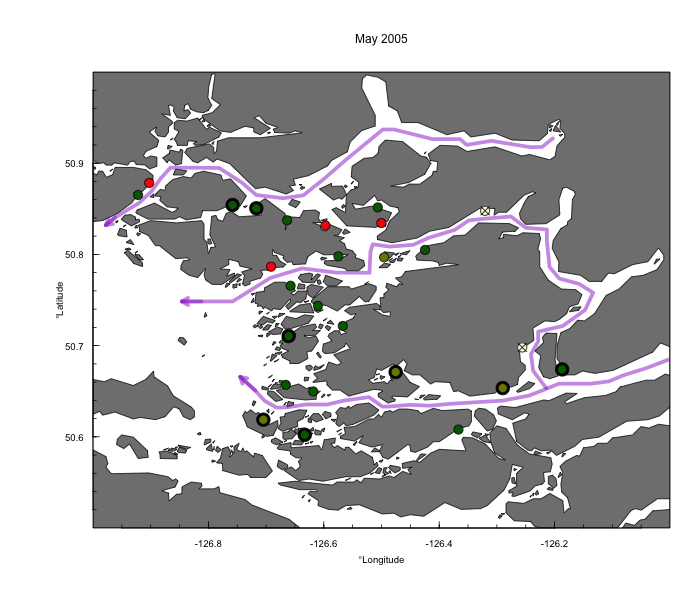

Supplement: Animation S1 — SLICE® treatments on Atlantic salmon farms in the Broughton Archipelago, British Columbia, Canada from November 1999 to December 2009 (Marty et al. 2010 PNAS). SLICE® treatment is indicated by a red circle at the salmon farm location. Untreated farms are shown by green circles, and farms fade from red to green over the four month efficacy period of SLICE® treatments (see main text). Fallowed farms (i.e., no Atlantic salmon in net pens) are indicated by beige circles with an ‘x’. Data that were used in the analysis of sea louse population dynamics before and after treatments (see main text) are circled in thick black. The period of juvenile wild salmon migration is indicated by purple arrows along approximate migration routes. (ZIP) [file pone.0060096.s001.zip › Animation S1/HTML_plot/BroughtonSLICE67.png]

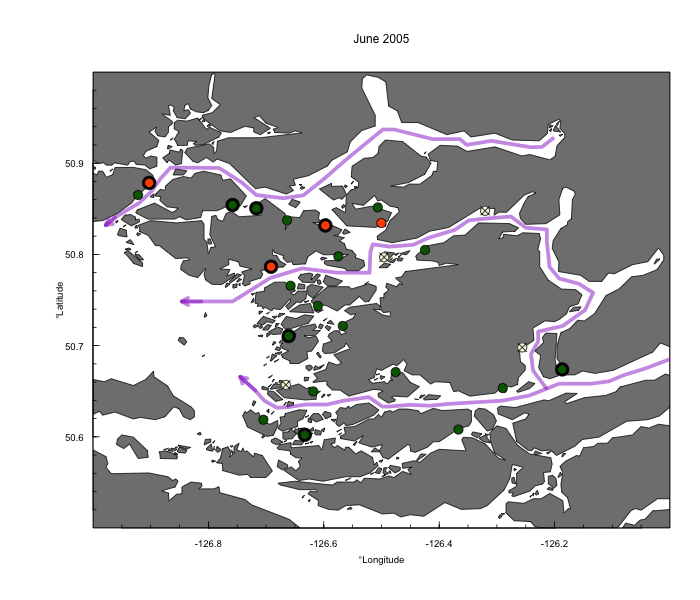

Supplement: Animation S1 — SLICE® treatments on Atlantic salmon farms in the Broughton Archipelago, British Columbia, Canada from November 1999 to December 2009 (Marty et al. 2010 PNAS). SLICE® treatment is indicated by a red circle at the salmon farm location. Untreated farms are shown by green circles, and farms fade from red to green over the four month efficacy period of SLICE® treatments (see main text). Fallowed farms (i.e., no Atlantic salmon in net pens) are indicated by beige circles with an ‘x’. Data that were used in the analysis of sea louse population dynamics before and after treatments (see main text) are circled in thick black. The period of juvenile wild salmon migration is indicated by purple arrows along approximate migration routes. (ZIP) [file pone.0060096.s001.zip › Animation S1/HTML_plot/BroughtonSLICE68.png]

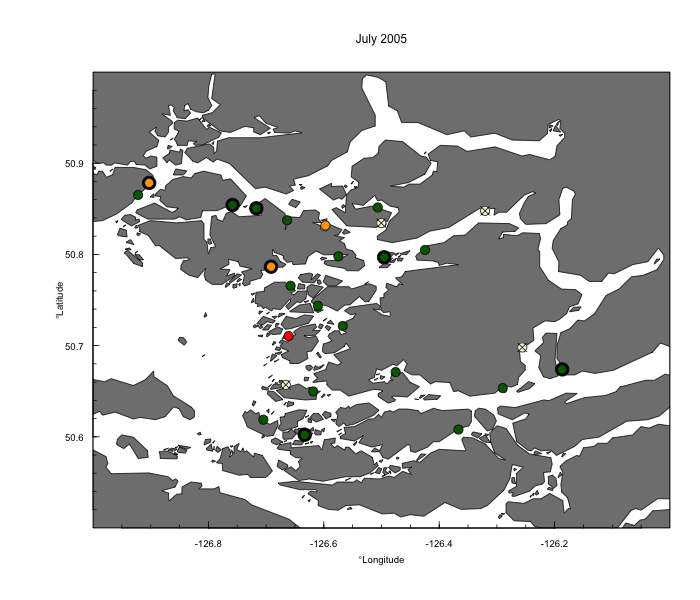

Supplement: Animation S1 — SLICE® treatments on Atlantic salmon farms in the Broughton Archipelago, British Columbia, Canada from November 1999 to December 2009 (Marty et al. 2010 PNAS). SLICE® treatment is indicated by a red circle at the salmon farm location. Untreated farms are shown by green circles, and farms fade from red to green over the four month efficacy period of SLICE® treatments (see main text). Fallowed farms (i.e., no Atlantic salmon in net pens) are indicated by beige circles with an ‘x’. Data that were used in the analysis of sea louse population dynamics before and after treatments (see main text) are circled in thick black. The period of juvenile wild salmon migration is indicated by purple arrows along approximate migration routes. (ZIP) [file pone.0060096.s001.zip › Animation S1/HTML_plot/BroughtonSLICE69.png]

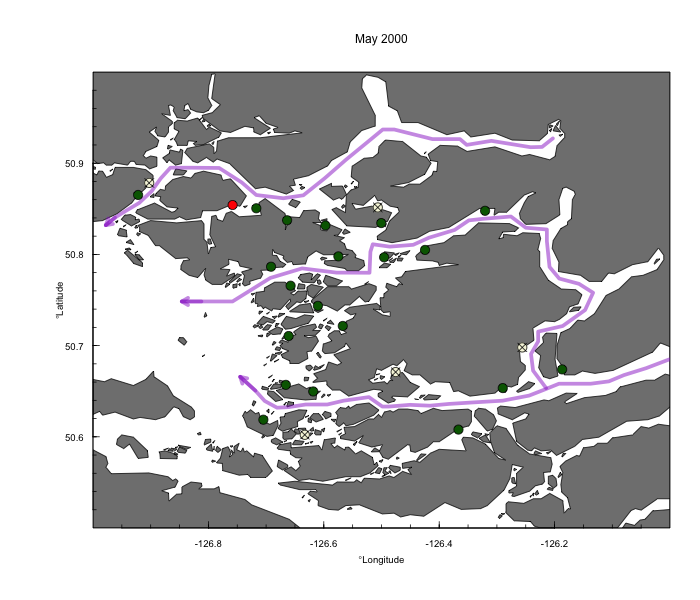

Supplement: Animation S1 — SLICE® treatments on Atlantic salmon farms in the Broughton Archipelago, British Columbia, Canada from November 1999 to December 2009 (Marty et al. 2010 PNAS). SLICE® treatment is indicated by a red circle at the salmon farm location. Untreated farms are shown by green circles, and farms fade from red to green over the four month efficacy period of SLICE® treatments (see main text). Fallowed farms (i.e., no Atlantic salmon in net pens) are indicated by beige circles with an ‘x’. Data that were used in the analysis of sea louse population dynamics before and after treatments (see main text) are circled in thick black. The period of juvenile wild salmon migration is indicated by purple arrows along approximate migration routes. (ZIP) [file pone.0060096.s001.zip › Animation S1/HTML_plot/BroughtonSLICE7.png]

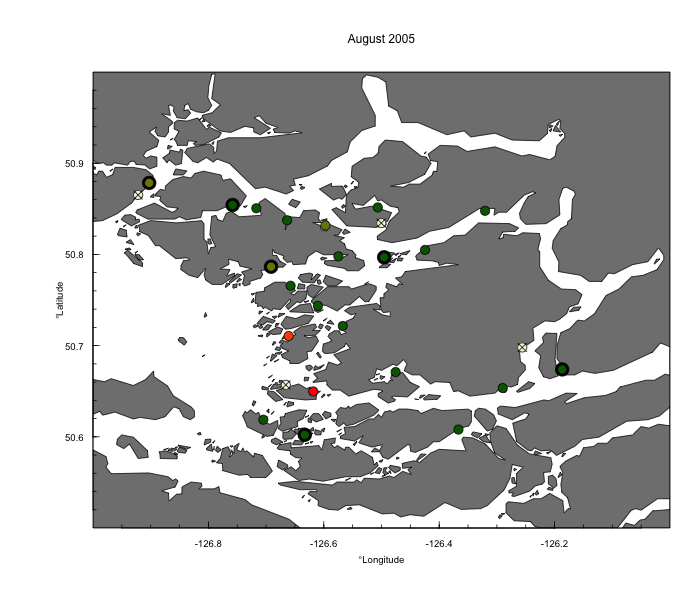

Supplement: Animation S1 — SLICE® treatments on Atlantic salmon farms in the Broughton Archipelago, British Columbia, Canada from November 1999 to December 2009 (Marty et al. 2010 PNAS). SLICE® treatment is indicated by a red circle at the salmon farm location. Untreated farms are shown by green circles, and farms fade from red to green over the four month efficacy period of SLICE® treatments (see main text). Fallowed farms (i.e., no Atlantic salmon in net pens) are indicated by beige circles with an ‘x’. Data that were used in the analysis of sea louse population dynamics before and after treatments (see main text) are circled in thick black. The period of juvenile wild salmon migration is indicated by purple arrows along approximate migration routes. (ZIP) [file pone.0060096.s001.zip › Animation S1/HTML_plot/BroughtonSLICE70.png]

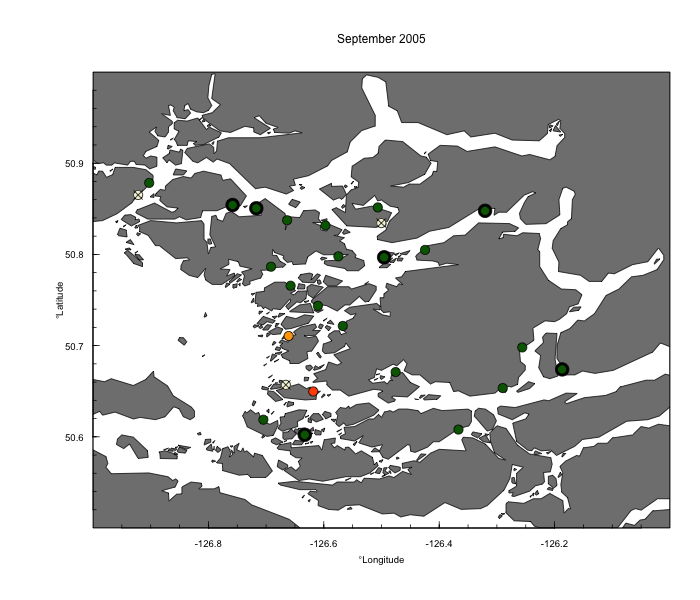

Supplement: Animation S1 — SLICE® treatments on Atlantic salmon farms in the Broughton Archipelago, British Columbia, Canada from November 1999 to December 2009 (Marty et al. 2010 PNAS). SLICE® treatment is indicated by a red circle at the salmon farm location. Untreated farms are shown by green circles, and farms fade from red to green over the four month efficacy period of SLICE® treatments (see main text). Fallowed farms (i.e., no Atlantic salmon in net pens) are indicated by beige circles with an ‘x’. Data that were used in the analysis of sea louse population dynamics before and after treatments (see main text) are circled in thick black. The period of juvenile wild salmon migration is indicated by purple arrows along approximate migration routes. (ZIP) [file pone.0060096.s001.zip › Animation S1/HTML_plot/BroughtonSLICE71.png]

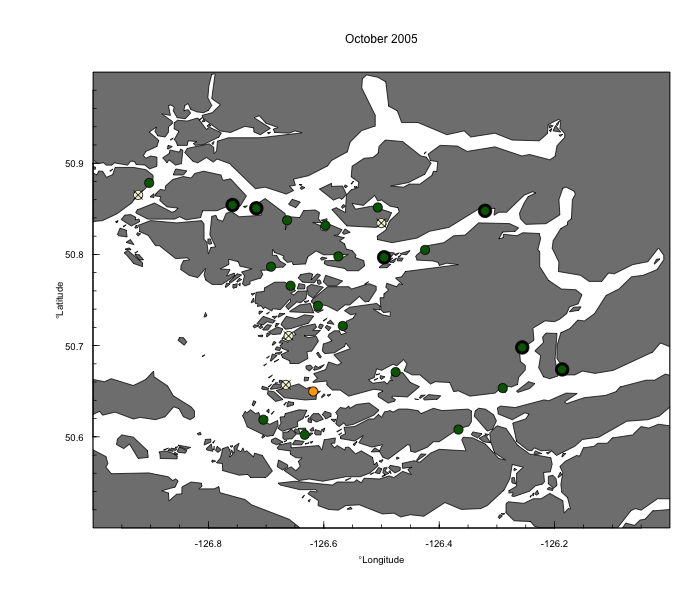

Supplement: Animation S1 — SLICE® treatments on Atlantic salmon farms in the Broughton Archipelago, British Columbia, Canada from November 1999 to December 2009 (Marty et al. 2010 PNAS). SLICE® treatment is indicated by a red circle at the salmon farm location. Untreated farms are shown by green circles, and farms fade from red to green over the four month efficacy period of SLICE® treatments (see main text). Fallowed farms (i.e., no Atlantic salmon in net pens) are indicated by beige circles with an ‘x’. Data that were used in the analysis of sea louse population dynamics before and after treatments (see main text) are circled in thick black. The period of juvenile wild salmon migration is indicated by purple arrows along approximate migration routes. (ZIP) [file pone.0060096.s001.zip › Animation S1/HTML_plot/BroughtonSLICE72.png]

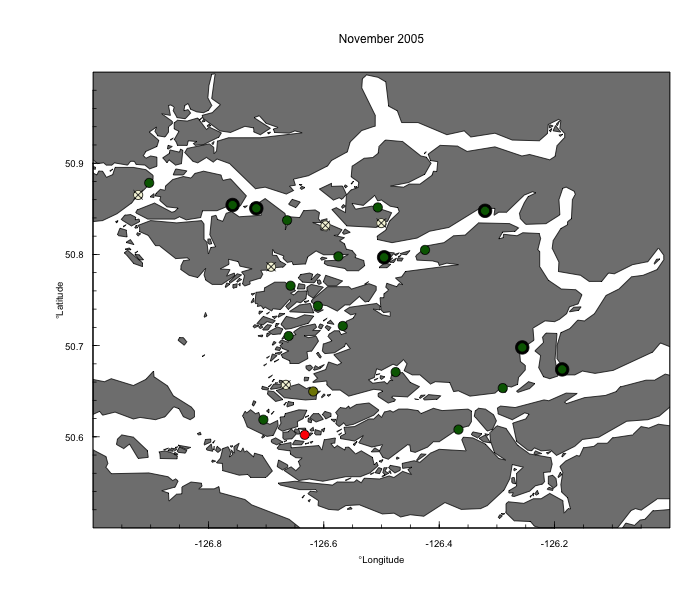

Supplement: Animation S1 — SLICE® treatments on Atlantic salmon farms in the Broughton Archipelago, British Columbia, Canada from November 1999 to December 2009 (Marty et al. 2010 PNAS). SLICE® treatment is indicated by a red circle at the salmon farm location. Untreated farms are shown by green circles, and farms fade from red to green over the four month efficacy period of SLICE® treatments (see main text). Fallowed farms (i.e., no Atlantic salmon in net pens) are indicated by beige circles with an ‘x’. Data that were used in the analysis of sea louse population dynamics before and after treatments (see main text) are circled in thick black. The period of juvenile wild salmon migration is indicated by purple arrows along approximate migration routes. (ZIP) [file pone.0060096.s001.zip › Animation S1/HTML_plot/BroughtonSLICE73.png]

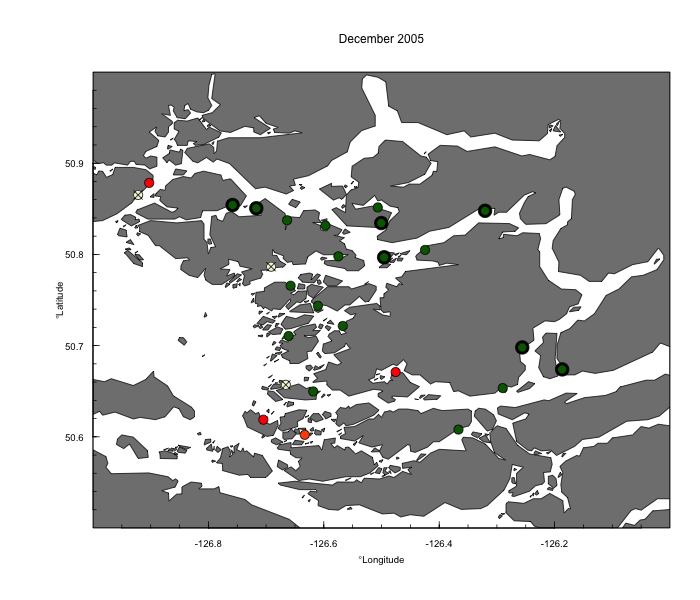

Supplement: Animation S1 — SLICE® treatments on Atlantic salmon farms in the Broughton Archipelago, British Columbia, Canada from November 1999 to December 2009 (Marty et al. 2010 PNAS). SLICE® treatment is indicated by a red circle at the salmon farm location. Untreated farms are shown by green circles, and farms fade from red to green over the four month efficacy period of SLICE® treatments (see main text). Fallowed farms (i.e., no Atlantic salmon in net pens) are indicated by beige circles with an ‘x’. Data that were used in the analysis of sea louse population dynamics before and after treatments (see main text) are circled in thick black. The period of juvenile wild salmon migration is indicated by purple arrows along approximate migration routes. (ZIP) [file pone.0060096.s001.zip › Animation S1/HTML_plot/BroughtonSLICE74.png]

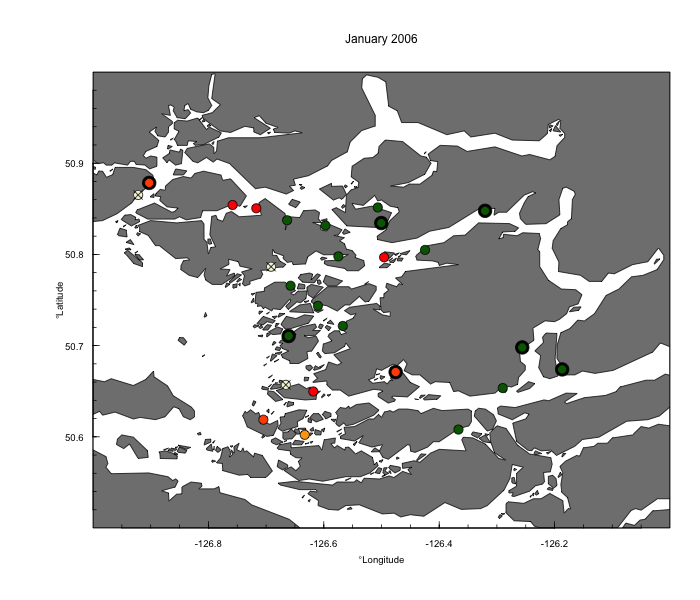

Supplement: Animation S1 — SLICE® treatments on Atlantic salmon farms in the Broughton Archipelago, British Columbia, Canada from November 1999 to December 2009 (Marty et al. 2010 PNAS). SLICE® treatment is indicated by a red circle at the salmon farm location. Untreated farms are shown by green circles, and farms fade from red to green over the four month efficacy period of SLICE® treatments (see main text). Fallowed farms (i.e., no Atlantic salmon in net pens) are indicated by beige circles with an ‘x’. Data that were used in the analysis of sea louse population dynamics before and after treatments (see main text) are circled in thick black. The period of juvenile wild salmon migration is indicated by purple arrows along approximate migration routes. (ZIP) [file pone.0060096.s001.zip › Animation S1/HTML_plot/BroughtonSLICE75.png]

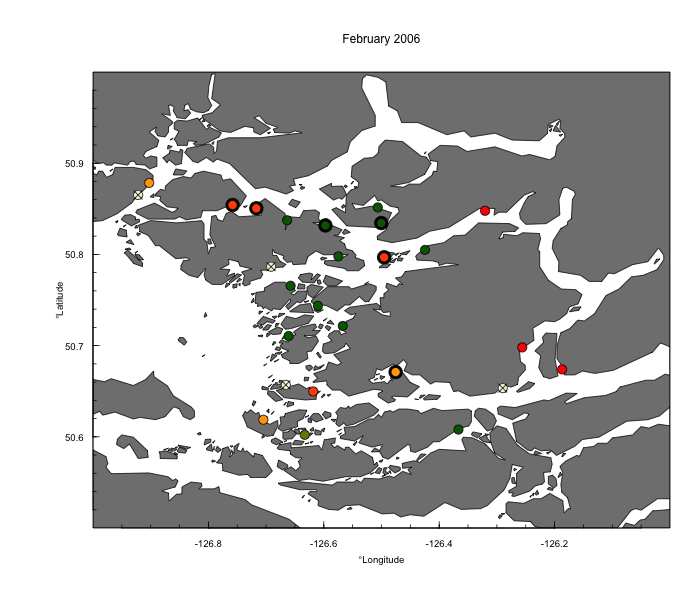

Supplement: Animation S1 — SLICE® treatments on Atlantic salmon farms in the Broughton Archipelago, British Columbia, Canada from November 1999 to December 2009 (Marty et al. 2010 PNAS). SLICE® treatment is indicated by a red circle at the salmon farm location. Untreated farms are shown by green circles, and farms fade from red to green over the four month efficacy period of SLICE® treatments (see main text). Fallowed farms (i.e., no Atlantic salmon in net pens) are indicated by beige circles with an ‘x’. Data that were used in the analysis of sea louse population dynamics before and after treatments (see main text) are circled in thick black. The period of juvenile wild salmon migration is indicated by purple arrows along approximate migration routes. (ZIP) [file pone.0060096.s001.zip › Animation S1/HTML_plot/BroughtonSLICE76.png]

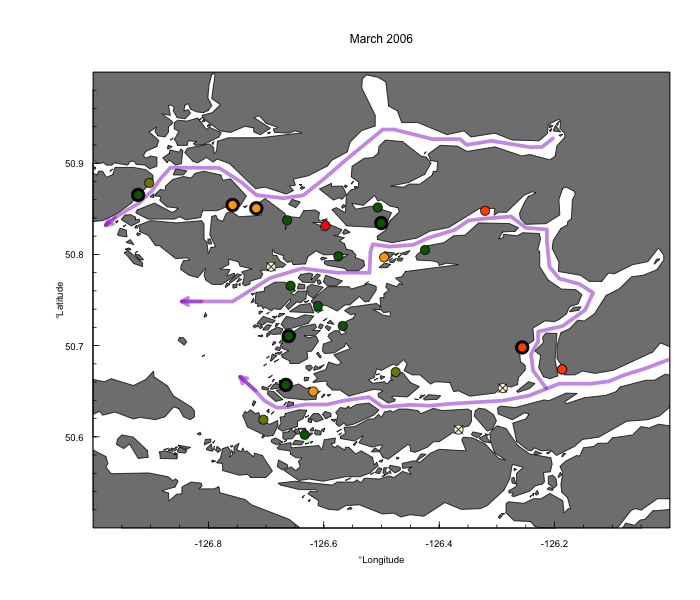

Supplement: Animation S1 — SLICE® treatments on Atlantic salmon farms in the Broughton Archipelago, British Columbia, Canada from November 1999 to December 2009 (Marty et al. 2010 PNAS). SLICE® treatment is indicated by a red circle at the salmon farm location. Untreated farms are shown by green circles, and farms fade from red to green over the four month efficacy period of SLICE® treatments (see main text). Fallowed farms (i.e., no Atlantic salmon in net pens) are indicated by beige circles with an ‘x’. Data that were used in the analysis of sea louse population dynamics before and after treatments (see main text) are circled in thick black. The period of juvenile wild salmon migration is indicated by purple arrows along approximate migration routes. (ZIP) [file pone.0060096.s001.zip › Animation S1/HTML_plot/BroughtonSLICE77.png]

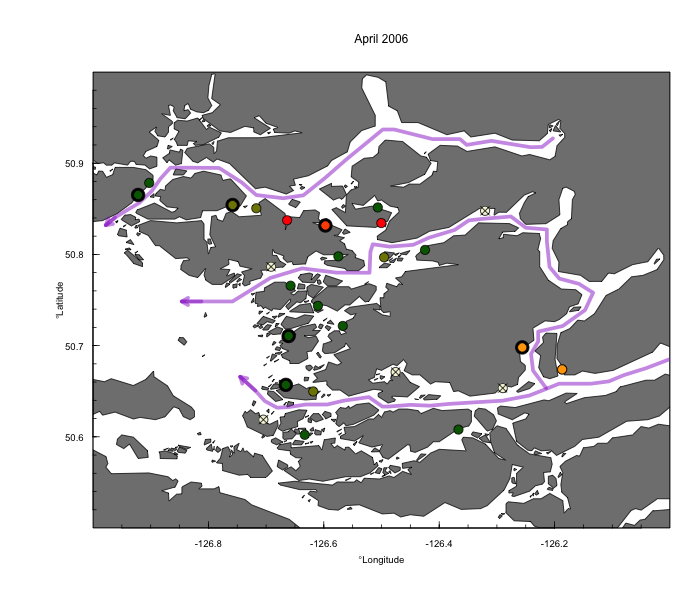

Supplement: Animation S1 — SLICE® treatments on Atlantic salmon farms in the Broughton Archipelago, British Columbia, Canada from November 1999 to December 2009 (Marty et al. 2010 PNAS). SLICE® treatment is indicated by a red circle at the salmon farm location. Untreated farms are shown by green circles, and farms fade from red to green over the four month efficacy period of SLICE® treatments (see main text). Fallowed farms (i.e., no Atlantic salmon in net pens) are indicated by beige circles with an ‘x’. Data that were used in the analysis of sea louse population dynamics before and after treatments (see main text) are circled in thick black. The period of juvenile wild salmon migration is indicated by purple arrows along approximate migration routes. (ZIP) [file pone.0060096.s001.zip › Animation S1/HTML_plot/BroughtonSLICE78.png]

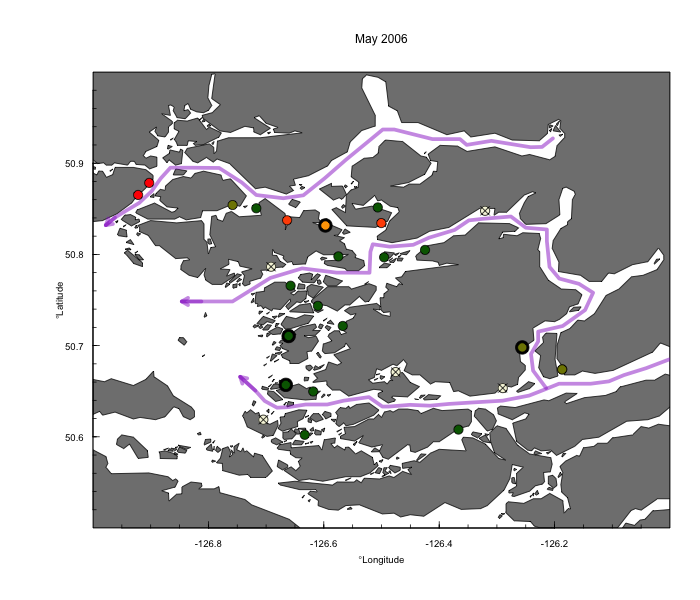

Supplement: Animation S1 — SLICE® treatments on Atlantic salmon farms in the Broughton Archipelago, British Columbia, Canada from November 1999 to December 2009 (Marty et al. 2010 PNAS). SLICE® treatment is indicated by a red circle at the salmon farm location. Untreated farms are shown by green circles, and farms fade from red to green over the four month efficacy period of SLICE® treatments (see main text). Fallowed farms (i.e., no Atlantic salmon in net pens) are indicated by beige circles with an ‘x’. Data that were used in the analysis of sea louse population dynamics before and after treatments (see main text) are circled in thick black. The period of juvenile wild salmon migration is indicated by purple arrows along approximate migration routes. (ZIP) [file pone.0060096.s001.zip › Animation S1/HTML_plot/BroughtonSLICE79.png]
